# Aerobic oxidative condensation for the synthesis of phenoxazinone and phenazine derivatives catalyzed by an iron-porphyrin complex with a pendant imidazole ligand

**DOI:** 10.1039/d6ra02674e

**Published:** 2026-05-05

**Authors:** Cheng Wang, Shuai-Chen Zhang, Jing Zhang, Ke Guo, Peng Sun

**Affiliations:** a State Key Laboratory for Quality Ensurance and Sustainable Use of Dao-di Herbs, Artemisinin Research Center and Institute of Chinese Materia Medica, China Academy of Chinese Medical Sciences Beijing 100700 People's Republic of China psun@icmm.ac.cn

## Abstract

Phenoxazinone and phenazine represent privileged *N*-heteroaromatic scaffolds that are ubiquitously prevalent in natural products, bioactive molecules, and advanced functional materials. Conventional synthetic methodologies for constructing these complex heterocyclic frameworks have relied heavily on stoichiometric amounts of strong oxidants. However, these classical approaches are inevitably accompanied by severe environmental and efficiency issues, including poor functional group tolerance, harsh reaction conditions. Inspired by the active site architecture of natural heme-containing metalloenzymes like cytochrome c oxidase, we designed and synthesized a novel biomimetic iron-porphyrin complex featuring a covalently tethered pendant imidazole. This imidazole acts as an axial ligand, perfectly mimicking the natural enzyme's confined microenvironment. This catalyst exhibits outstanding activity for the aerobic oxidative cyclocondensation of *o*-aminophenols and *o*-phenylenediamines. The system demonstrates broad functional group tolerance, affording diverse phenoxazinone and phenazine derivatives in excellent yields using air as the terminal oxidant. This study offers a highly efficient, sustainable paradigm for biomimetic oxidation catalysis.

## Abstract 2

A biomimetic iron-porphyrin catalyst with a imidazole axial ligand has been developed to catalyze oxidative condensation of *o*-aminophenols and *o*-phenylenediamines to synthesize phenoxazinone and phenazine derivatives using air as oxidant.

## Graphical abstract

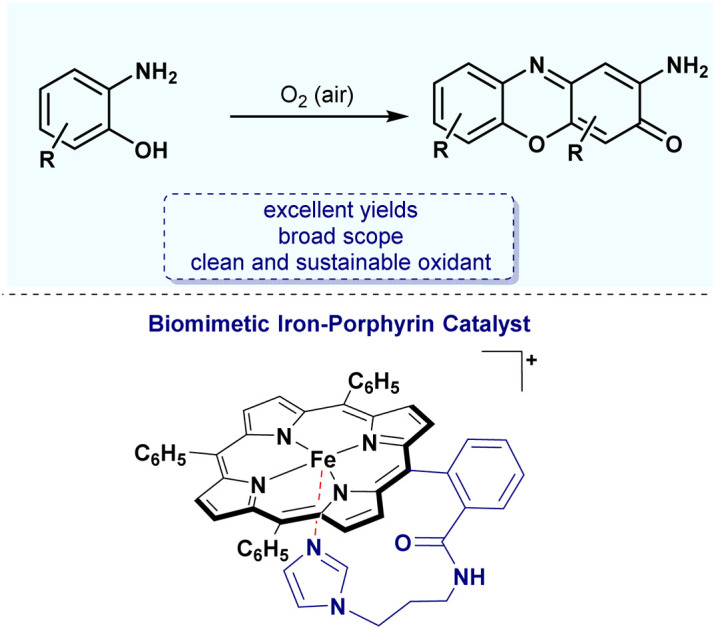

## Introduction

In the broad fields of organic synthesis and medicinal chemistry, phenoxazinone and its related phenazine derivatives are widely recognized as highly valuable privileged *N*-heteroaromatic scaffolds.^1,2^ These compounds have established an irreplaceable core status in various interdisciplinary fields, such as chemical biology, drug development, and materials science. Specifically, the 2-aminophenoxazin-3-one scaffold is the core structural motif of numerous natural products and pharmaceutical agents, such as actinomycin D, questiomycin A.^3–6^ Concurrently, the structurally analogous phenazine scaffold is renowned for its highly stable and redox-active π-conjugated system, making them highly sought after in cutting-edge technological fields such as organic electronics, organic light-emitting diodes (OLEDs), and chemical sensing materials.^7,8^ Ultimately, the profound multifunctionality and broad applicability of these distinct core frameworks continuously drive synthetic chemists to explore and develop more efficient synthetic methodologies to meet the growing demands for materials and pharmaceuticals.^9,10^

**Scheme 1:**
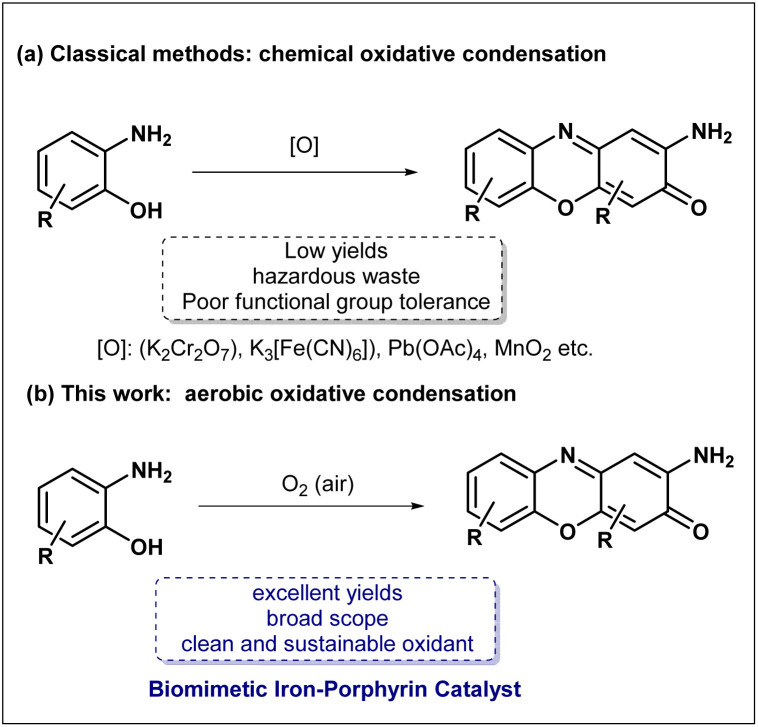
Oxidative cyclocondensation of *o*-aminophenols for the constuction of phenoxazinone.

The construction of these complex scaffolds typically proceeds *via* the oxidative cyclocondensation of *o*-aminophenols or *o*-phenylenediamines.^11^ However, conventional synthetic strategies for this specific chemical transformation have long relied on stoichiometric quantities of potent, and often highly toxic, inorganic oxidants. Over the past few decades, reagents such as potassium dichromate (K_2_Cr_2_O_7_), potassium ferricyanide (K_3_[Fe(CN)_6_]), lead tetraacetate (Pb(OAc)_4_), manganese dioxide (MnO_2_) have been employed (Scheme 1a).^12,13^ However, these approaches are fundamentally limited by poor functional group tolerance, low yields, and the generation of significant hazardous waste.^12,13^ To address these persistent deficiencies and align the synthetic processes closely with the concept of sustainable green chemistry, many leading research groups worldwide have recently dedicated substantial efforts to developing catalytic aerobic oxidation methods.^14–19^ The central goal of these strategies is to utilize molecular oxygen (O_2_)—the most abundant, cheap, and environmentally benign oxidant—as the terminal oxidant, thereby generating only water as the sole byproduct. Nevertheless, achieving efficient activation of ground-state triplet dioxygen under mild conditions remains a formidable challenge in synthetic chemistry, primarily due to the inherent spin-forbidden nature of direct reactions between triplet O_2_ and singlet organic substrates. In highly complex biological systems, the fundamental activation of ground-state triplet dioxygen is elegantly and efficiently orchestrated by a series of highly specialized heme-containing metalloenzymes. Prominent representative among these remarkable biocatalysts is the ubiquitous cytochrome P450 superfamily.^20,21^ Inspired by the catalytic mode of these enzymes, researchers have extensively designed and investigated numerous synthetic iron porphyrins and other related metal macrocycles as biomimetic oxidation catalysts.^22–26^ High-resolution crystallographic analyses of natural enzyme structures have revealed a crucial architectural feature: an imidazole group from a proximal histidine residue serves as an axial ligand, coordinating directly to the central heme iron ion with fixed spatial orientation.^27^ Pioneering studies by Collman^28^ and Dey^29^ have demonstrated that this specific axial imidazole ligand exerts a profound electronic influence on the central iron center-commonly termed the “push effect”-which dramatically facilitates the activation of O_2_ and the subsequent generation of highly reactive iron-oxo intermediates. Building upon these profound structural features and complex mechanistic insights, we proposed that if a functional imidazole ligand could be covalently tethered and anchored to a rigid porphyrin macrocycle *via* a conformationally flexible linker, it would be possible to successfully mimic the compact, confined microenvironment and highly optimized coordination geometry of the natural enzyme's active site at the molecular level. Compared to common untethered iron porphyrins, this engineered biomimetic system would possess a permanently pre-organized axial coordination environment, theoretically enabling more efficient and stable catalytic aerobic oxidation.^30^ Herein, we report the rational biomimetic design, target-oriented synthesis, and comprehensive application of this novel iron-porphyrin complex bearing a pendant imidazole ligand (Scheme 1b). Under mild reaction conditions, utilizing ambient air as the sole and terminal oxidant, this bio-inspired catalytic system highly efficiently drives the aerobic oxidative condensation of various *o*-aminophenols and *o*-phenylenediamines, delivering the corresponding phenoxazinone and phenazine derivatives in excellent yields.

## Result and discussion

**Scheme 2:**
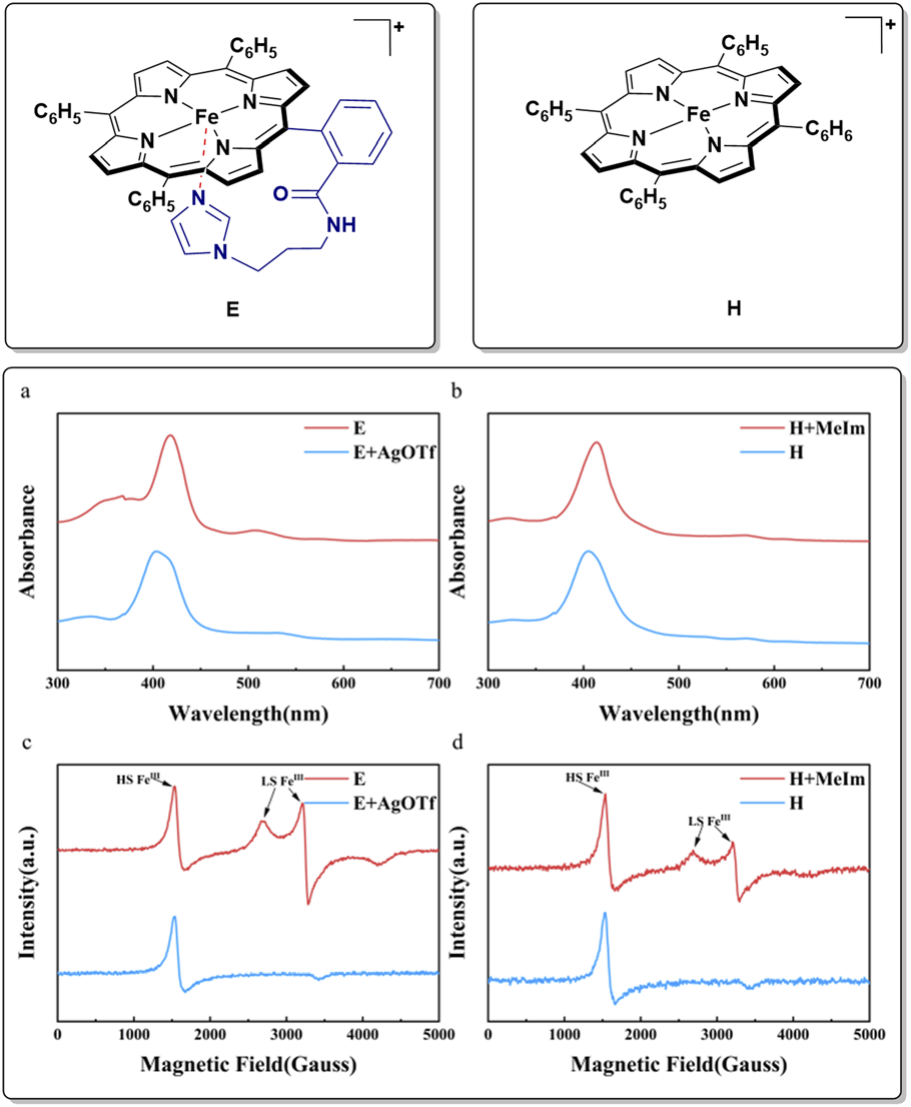
The chemical structure of the synthesized catalyst and the UV/vis, EPR spectra of comparative examples. (a) and (b) are the UV/vis spectra of E and H, (c) and (d) are the EPR spectrum of E and H.

We successfully designed and synthesized the target iron-porphyrin complex E (Scheme 2), which features a highly conformationally flexible, covalently tethered imidazole axial ligand. Simultaneously, we synthesized a conventional iron-porphyrin complex H (Scheme 2), which completely lacks this pendant structural feature, as a strict experimental control group. The identities and purities of the catalyst were confirmed by high-resolution mass spectrometry and supercritical fluid chromatography. Extensive ultraviolet-visible (UV/vis) absorption spectroscopy and electron paramagnetic resonance (EPR) spectroscopy studies were conducted to validate the axial coordination of the tethered imidazole ligand to the central iron ion through reported method (Scheme 2).^31^ As shown in (Scheme 2a and b), the UV/vis spectra of E and H exhibit distinct Soret bands, reflecting different coordination environments. Notably, addition of one equivalent of *N*-methylimidazole (NMI) to the solution of H resulted in a red-shifted of the maximum absorption wavelength, which was similar to E. To further probe the stability and the intramolecular coordination in complex E, we designed competitive chemical experiments. When excess AgOTf was added to the solution of E, its UV/vis spectrum rapidly underwent a significant blue-shift. The optical features degraded almost entirely to a state identical to that of the uncoordinated complex H. This spectral transition is fundamentally attributed to the highly competitive and preferential binding of Ag^+^ ions with the ligand. In contrast, the addition of sodium trifluoromethanesulfonate (NaOTf) to complex E induced no obvious spectral shifts. Because Na^+^ lacks the strong affinity for nitrogen-containing organic ligands exhibited by Ag^+^. Through electron paramagnetic resonance (EPR) spectroscopy, we further deciphered the subtle electronic structure of the metal center. As shown in (Scheme 2c and d), the EPR spectrum of E displayed signals corresponding to both high-spin (HS) and low-spin (LS) Fe^III^ states, whereas H exhibited only HS Fe^III^ features. This difference likely arises from dynamic coordination and dissociation of the triflate anion in E, establishing an equilibrium between six-coordinate LS and five-coordinate HS species. Consistent with the UV/vis observations, addition of one equivalent of *N*-methylimidazole to H generated an EPR spectrum similar to that of E, indicating imidazole coordination to the iron center. When excess AgOTf was added to E, the original LS signals disappeared, confirming imidazole dissociation, whereas excess NaOTf caused no appreciable change in the EPR spectrum. Collectively, these experiments confirm that the tethered imidazole ligand in E coordinates axially to the iron center.

Having rigorously established the structural integrity and biomimetic nature of catalyst E, we turned to evaluate the catalytic performance of the complex. We selected the aerobic oxidative condensation of *o*-aminophenol (1a) to 2-aminophenoxazin-3-one (2a) as the model reaction. Initial parameter screening commenced with a 1 mol% catalyst E loading under ambient air conditions. First, the solvent media for the oxidative coupling was screened systematically. Halogenated hydrocarbons DCM and DCE delivered the product in the yield of about 20% (entries 1 and 2). Aprotic polar solvents including acetonitrile, DMF, and DMSO provided yields in the range of 25–31% (entries 3–5). Polar protic aliphatic alcohol solvents, such as methanol (MeOH) and ethanol (EtOH), provided moderate product yields (35–42%, entries 6 and 7). The highly fluorinated specialty solvents 2,2,2-trifluoroethanol (TFE) demonstrated absolutely outstanding performance, delivering the target phenoxazinone product in a yield of 53% (entry 8). Next, we conducted meticulous temperature gradient optimization studies in the TFE solvent (entries 9–12). An exceptionally high isolated yield of 86% was obtained at the temperature of 60 °C. Astonishingly, the porphyrin complex H, which completely lacks the axial imidazole ligand, exhibited poor catalytic performance under this condition, yielding a yield of 24% (entry 13). The combination of exogenous (NMI) with the unmodified catalyst H failed to recover the efficacy of E (entry 14). Moreover, benchmark tests conducted with other non-specific iron catalysts revealed similarly poor catalytic performances, affording target product yields of only 49%, 33%, and 58%, significantly inferior to the performance of E (entries 15–17). Furthermore, scaling the reaction to 10 mmol (1.09 g) of substrate with only 0.1 mol% of catalyst E at 60 °C under air for 36 hours achieved a remarkable 88% yield. These results collectively demonstrate the robust catalytic activity of E (Table 1).

**Table 1:** Optimization of the reaction condition for the oxidative condensation of *o*-aminophenol

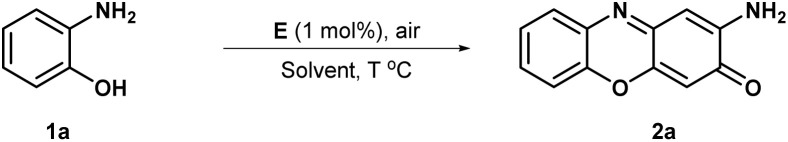				
Entry^a^	Catalyst	Solvent	Temp. (°C)	Yield^b^ %
1	E	DCM	25	21
2	E	DCE	25	20
3	E	MeCN	25	30
4	E	DMF	25	31
5	E	DMSO	25	25
6	E	MeOH	25	39
7	E	EtOH	25	38
8	E	TFE	40	68
9	E	TFE	60	86
10	E	TFE	80	62
11	E	TFE	100	39
12	E	TFE	60	86
13	H	TFE	60	24
14	H+NMI^c^	TFE	60	15
15	Cat.1	TFE	60	49
16	Cat.2	TFE	60	33
17	Cat.3	TFE	60	58
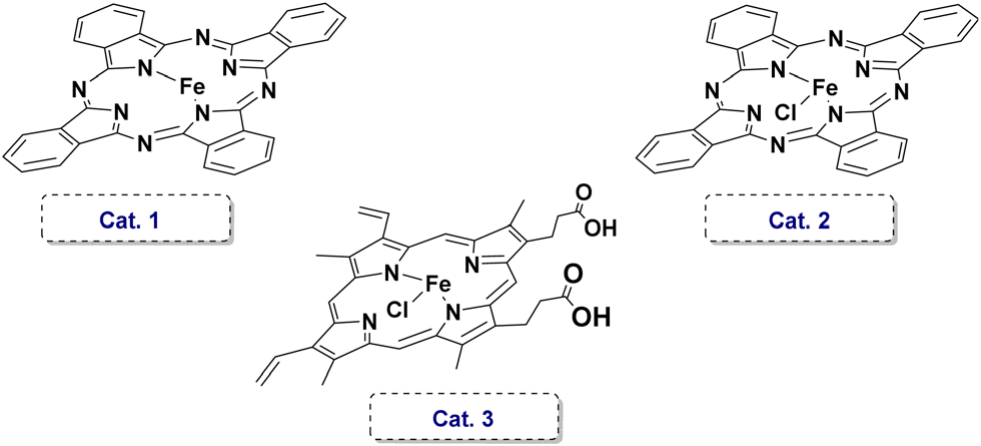				

a Reaction conditions: 1a (1.0 mmol, 1.0 equiv.), catalyst (0.01 mmol), solvent (10 mL), air condition.

b Isolated yields.

c 0.01 mmol of NMI was added.

**Scheme 3:**
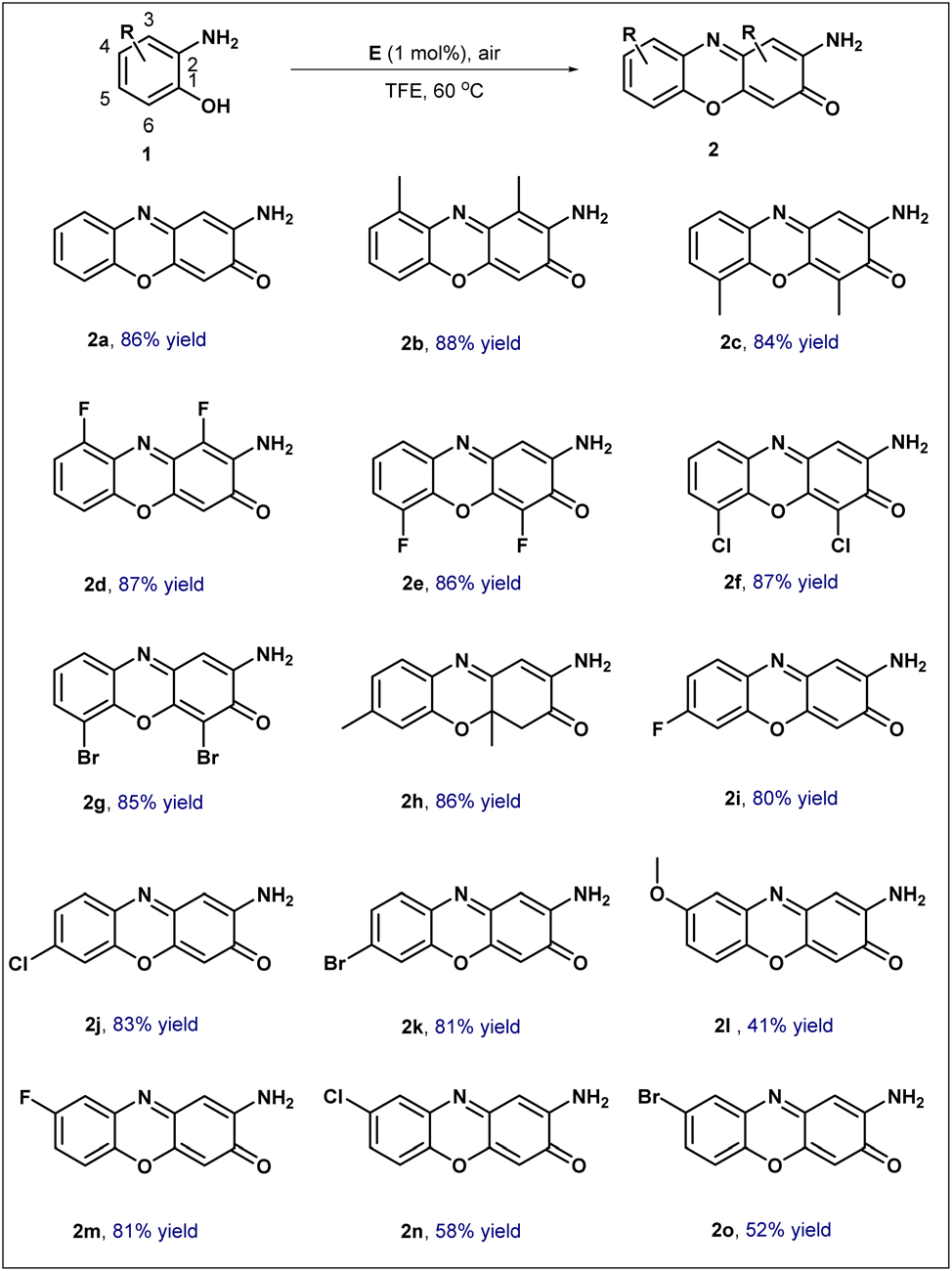
The substrate scope for the oxidation of various substituted *o*-aminophenols.

Under the optimized reaction conditions, the substrate scope of this transformation was further investigated using a series of substituted *o*-aminophenols (Scheme 3). Initially, substrates bearing a methyl group at the C3 or C6 position of the benzene ring underwent smooth conversion, affording the corresponding products 2b and 2c in approximately 85% yield.

The influence of halogen substituents at these positions was subsequently examined. Gratifyingly, even with electron-withdrawing halogen atoms at the C3 or C6 position, the desired products 2d, 2e, 2f, and 2g were consistently obtained in 85% yield or higher. Attention was then turned to substitution at the C5 position. When a methyl group was introduced at this site, the reaction furnished the dihydro derivative 2h, which retained both methyl groups, in a commendable 86% yield. Notably, substrates with electron-withdrawing halogen substituents at the C5 position also performed well, delivering the corresponding products 2i, 2j, and 2k in yields exceeding 80%; this result compares favorably with previously reported electrochemical methods.^15^ Finally, the impact of substituents at the C4 position was tested. The presence of an electron-donating methoxy group at this position resulted in a modest 41% yield of product 2l. A large amount of black tarry material was formed, which might be attributed to the strong electron-donating effect of the methoxy group that likely promotes over-oxidation and subsequent polymerization under the catalytic oxidative conditions. While, electron-withdrawing halogen substituents exhibited varying effects: the fluoro-substituted substrate afforded product 2m in 81% yield, whereas its chloro- and bromo-substituted counterparts furnished products 2n and 2o in approximately 50% yield.

**Scheme 4:**
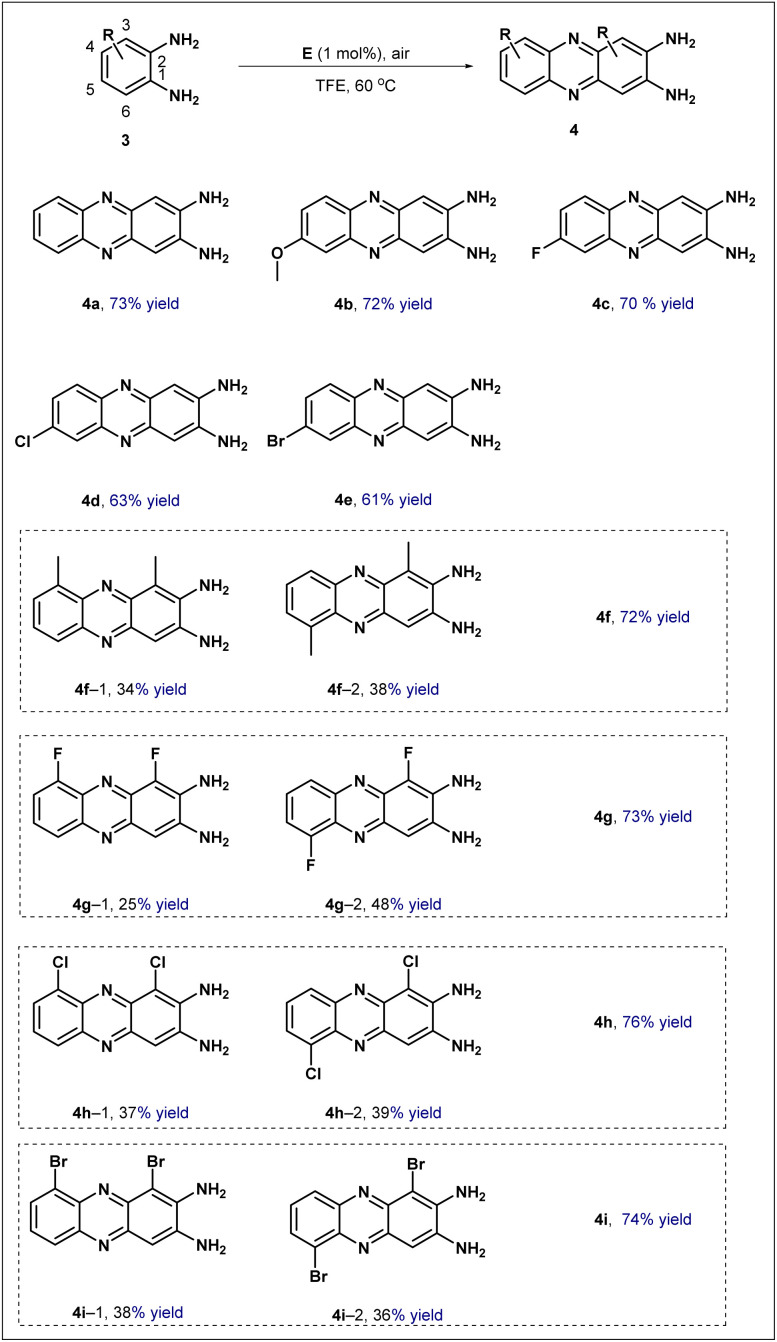
The substrate scope for the oxidation of various substituted *o*-phenylenediamines.

To evaluate the generality of this catalytic system, the optimized reaction conditions were extended to the oxidative condensation of *o*-phenylenediamines (Scheme 4). Initially, *o*-phenylenediamine(3a) underwent efficient conversion, affording the corresponding 2,3-diaminophenazine 4a in 73% yield. The influence of substituents was then examined. Introduction of an electron-donating methoxy group at the C4 position delivered product 4b in 72% yield, while substrates bearing electron-withdrawing halogen substituents at the same position furnished the desired products 4c, 4d, and 4e in 70%, 63%, and 61% yield, respectively. Finally, the reaction behavior of substrates with substituents at the C3 position was investigated. In these cases, the transformation consistently generated a pair of isomers; regardless of whether the substituent was electron-donating or electron-withdrawing, the corresponding isomeric pairs 4f, 4g, 4h, and 4i were all obtained in good combined yields exceeding 70%. Collectively, these results convincingly demonstrate that the synthesized catalyst exhibits a truly excellent substrate scope, successfully accommodating a remarkably wide range of substituents possessing varying electronic as well as steric demands.

**Scheme 5:**
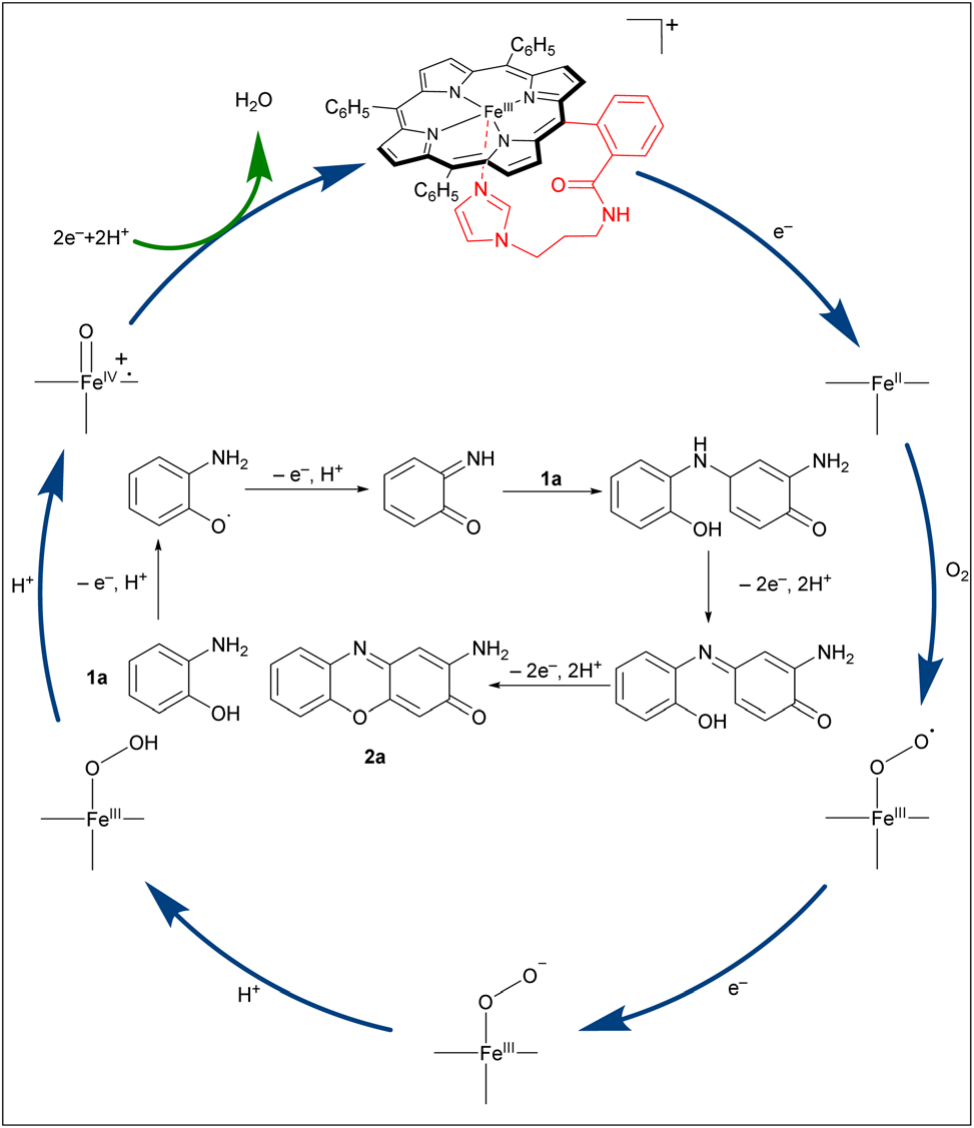
Proposed reaction mechanism.

Based on the above research^15,26^ and literature reports,^32,33^ a plausible mechanism for the oxidation process is proposed in Scheme 5. Initially, *o*-aminophenol donates an electron to reduce Fe(iii) to Fe(ii), enabling oxygen binding, while itself being converted to a radical that rapidly undergoes coupling. A second electron, usually from another *o*-aminophenol molecule (or a dimer intermediate), is then required to form the Fe(iii)-O_2_˙. Using protons released upon substrate oxidation, high-valent oxo-iron species, Por^+^˙Fe(iv)=O is subsequently generated. The imidazole coordination is crucial for both stabilizing this powerful oxidant and tuning its reactivity. This species then mediates a single electron oxidation of *o*-aminophenol, generating a 2-aminophenoxyl radical. Subsequent loss of an electron and a proton from this radical affords the key intermediate, *o*-benzoquinone monoimine. Another molecule of *o*-aminophenol undergoes 1,4-conjugate addition to this intermediate, followed by oxidative dehydrogenation, yielding a *p*-quinone imine derivative. Finally, dehydrogenative cyclization furnishes the corresponding 2-aminophenoxazin-3-one product. This proposed pathway highlights how the engineered imidazole axial ligand not only stabilizes the catalytically active iron-oxo species but also facilitates the electron-transfer steps essential for efficient O_2_ activation and substrate oxidation.

## Conclusions

In summary, we have developed a well-defined biomimetic iron-porphyrin catalyst featuring a covalently tethered axial imidazole ligand. This complex efficiently drives the aerobic oxidative cyclocondensation of *o*-aminophenols and *o*-phenylenediamines, yielding 2-aminophenoxazin-3-ones and 2,3-diaminophenazines with excellent selectivity and high yields. Operating under ambient air with a remarkably low catalyst loading of 0.1 mol%, the system demonstrates exceptional practical utility, highlighted by its successful application in gram-scale synthesis. Ultimately, this work establishes that replicating an enzyme-inspired, stabilized axial coordination environment is critical for designing robust aerobic oxidation catalysts. This biomimetic strategy offers a highly tunable and sustainable platform for advancing green chemical synthesis.

## Author contributions

Cheng Wang: conceptualization, methodology, investigation, formal analysis, writing – original draft; Shuai-Chen Zhang: investigation, data curation, writing – review & editing; Jing Zhang: methodology, formal analysis, writing-review & editing; Ke Guo: validation, writing – review & editing; Peng Sun: conceptualization, methodology, resources, supervision, project administration, funding acquisition, writing-review & editing. All authors participated in drafting the manuscript and have read and consented to the final published version.

## Conflicts of interest

There are no conflicts to declare.

## Supplementary Material

RA-016-D6RA02674E-s001

## Data Availability

The data that support the findings of this study are available within the main manuscript and its supplementary information (SI). Additional data related to this work are available from the corresponding author upon reasonable request. Supplementary information: experimental procedures and characterization data for all synthesized compounds. Catalyst synthesis and spectroscopic characterization (HRMS, HPLC, UV/vis, EPR). Reaction optimization tables and substrate scope data. Copies of ^1^H and ^13^C NMR spectra. See DOI: https://doi.org/10.1039/d6ra02674e.
