# Supplementary material for: Aerobic oxidative condensation for the synthesis of phenoxazinone and phenazine derivatives catalyzed by an iron-porphyrin complex with a pendant imidazole ligand
Source: RSC Adv. 2026 May 5;16(25):23095–100. doi: 10.1039/d6ra02674e (PMC13139906; doi:10.1039/d6ra02674e)
Supplement: RA-016-D6RA02674E-s001 [file RA-016-D6RA02674E-s001.pdf]

Supplementary Information (SI) for RSC Advances.
This journal is © The Royal Society of Chemistry 2026

Supporting Information

Aerobic Oxidative Condensation for the Synthesis of Phenoxazinone and Phenazine Derivatives Catalyzed by an Iron-Porphyrin Complex with a Pendant Imidazole Ligand

Cheng Wang,[a] Shuai-Chen Zhang,[a] Jing Zhang,[a] Ke Guo,[a] Peng Sun[a]*

[a] State Key Laboratory for Quality Ensurance and Sustainable Use of Dao-di Herbs; Artemisinin Research Center and Institute of Chinese Materia Medica, China Academy of Chinese Medical Sciences, Beijing 100700, People's Republic of China.

* Corresponding author

Email: psun@icmm.ac.cn

Contents

1. General Information 3
2. Synthesis of Fe porphyrins 4
3. Characterization of Fe Porphyrins...9
4. General Procedure 11
5. References 19
6. ^{1}H and ^{13}C NMR spectra 20

1. General Information

Unless otherwise noted, reagents were obtained from commercial sources and utilized without further purification. All characterizations were performed using the following instruments: Nuclear magnetic resonance spectroscopy was conducted on a Bruker AV600 MHz spectrometer from Switzerland to acquire ^{1}H and ^{13}C NMR spectra. High-resolution mass spectrometry was performed using a Waters ACQUITY I-class ultra-performance liquid chromatography system coupled to a Waters Xevo G2-XS QToF mass spectrometer, both sourced from the United States, in order to obtain precise molecular ion information. UV/Vis absorption spectra were measured using a UV1810 spectrophotometer manufactured by Youyi Technology in China. Electron paramagnetic resonance spectra were acquired on a Bruker A300-10/12 spectrometer from Germany; the system was equipped with a liquid nitrogen cooling unit and operated at a microwave frequency of 9.46 GHz. Catalyst purity analysis was performed on a supercritical fluid chromatography–mass spectrometry system, model SFC-30ADMS. Finally, sample separation and purification were accomplished using a rapid preparative liquid chromatography system, specifically the SepaBean machine, supplied by Changzhou Santai Technology Co., Ltd in China. Specific experimental parameters for each measurement are detailed in the corresponding figure captions or sections below.

2. Synthesis of Fe porphyrins

Fe porphyrin **E** was synthesized using synthetic route depicted in Scheme S1.

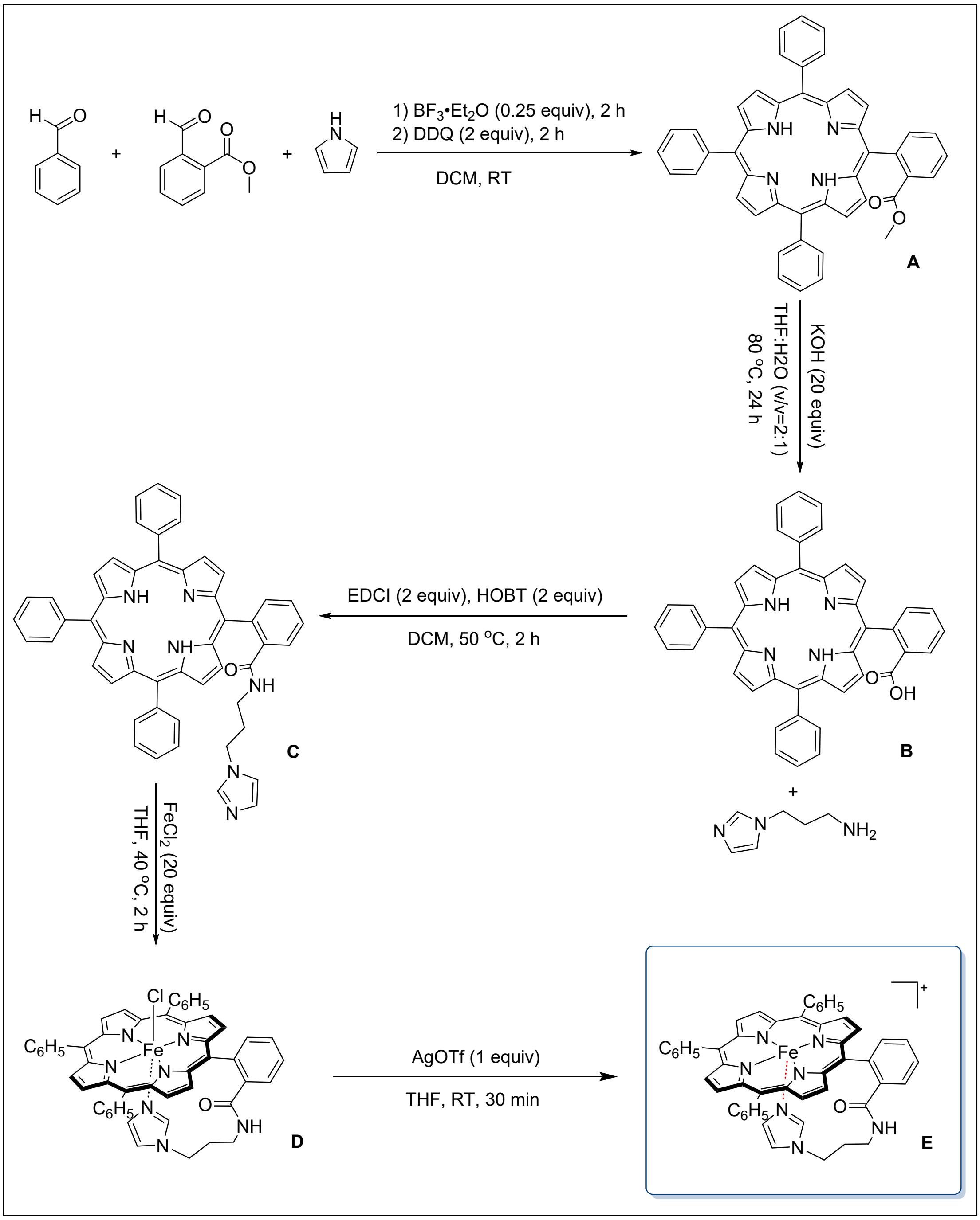

Scheme S1. Synthetic route of Fe porphyrin **E**.

In the experimental procedure, methyl 2-formylbenzoate (4 mmol, 0.56 mL), benzaldehyde (12 mmol, 1.2 mL), and pyrrole (16 mmol, 1.1 mL) were dissolved in dichloromethane (200 mL) under stirring. Boron trifluoride diethyl etherate (1 mmol, 0.26 mL) was then added to the mixture. After stirring for 2 hours, 2,3-dichloro-5,6-dicyano-1,4-benzoquinone (DDQ, 8 mmol, 1.8 g) was added, and the reaction was continued for an additional 2 hours[1–3]. The reaction was quenched by

the addition of a small amount of triethylamine. The crude product was purified by column chromatography using a 1:1 (v/v) mixture of dichloromethane and petroleum ether as the eluent to afford compound **A** in 15% yield.

Compound **A** (1 mmol, 0.67 g) was dissolved in tetrahydrofuran (60 mL). To this solution was added an aqueous solution of potassium hydroxide (KOH, 20 mmol, 1.12 g in 30 mL of water), followed by a few drops of methanol. The mixture was heated to reflux at 80 ^{o}C for 24 hours. After cooling to room temperature, the reaction mixture was neutralized with 1 M hydrochloric acid (HCl) solution. The resulting mixture was then extracted three times with saturated sodium bicarbonate ($NaHCO_3$) solution and three times with water. The combined organic layers were dried over anhydrous sodium sulfate (Na_2SO_4). The crude product was purified by column chromatography using a 20:1 (v/v) mixture of dichloromethane and methanol as the eluent, affording compound **B** with a yield of 88%. The product was used directly in the subsequent step.

Compound **B** (1 mmol, 0.65 g) was dissolved in dichloromethane (30 mL). After complete dissolution, EDCI (2 mmol, 0.38 g) and HOBt (2 mmol, 0.27 g) were added sequentially. The reaction mixture was heated to reflux at 50 ^{o}C for 30 min. Subsequently, 1-(3-aminopropyl) imidazole (2 mmol, 0.23 mL) was added, and the reaction was continued for an additional 2 hours. Upon completion, the mixture was extracted three times with saturated brine and three times with water. The combined organic layers were dried over anhydrous sodium sulfate. The crude product was purified by column chromatography using a 10:1 (v/v) mixture of dichloromethane and methanol as the eluent, affording the target compound **C** in 85% yield.

Compound **C** (0.3 mmol, 0.23 g) and iron (II) chloride ($FeCl_2$, 6 mmol, 0.76 g) were dissolved in tetrahydrofuran (18 mL) under a nitrogen atmosphere[4]. The mixture was heated to reflux at 40 ^{o}C for 2 hours. After completion of the reaction, the crude product was purified by column chromatography using a 10:1 (v/v) mixture of dichloromethane and methanol as the eluent, affording the chloride-containing compound **D** : **(E)Cl$^-$** in 89% yield. The chloride ion in compound **D** was then substituted by a triflate group via reaction with an equimolar amount of silver trifluoromethanesulfonate (AgOTf) to give compound **E**.

methyl (R)-2-(10,15,20-triphenylporphyrin-5-yl) benzoate (A)

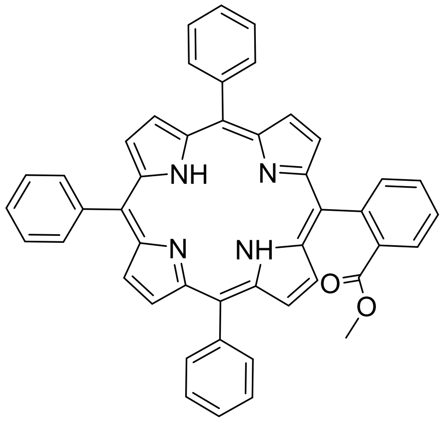

Purple solid, yield = 15%; **^{1}H NMR** (600 MHz, $CDCl_3$) δ 8.90 – 8.87 (m, 6H), 8.72 (d, *J* = 4.7 Hz, 2H), 8.44 (dd, *J* = 8.0, 1.5 Hz, 1H), 8.32 – 8.19 (m, 7H), 7.91 – 7.75 (m, 11H), 2.83 (s, 3H), –2.60 (s, 2H); **^{13}C NMR** (151 MHz, $CDCl_3$) δ 168.05, 142.76, 142.40, 142.31, 136.18, 134.75, 134.68,

134.67, 134.26, 129.87, 129.82, 128.50, 127.84, 127.83, 126.81, 120.28, 120.26, 119.18, 51.71; **HRMS (ESI):** Calcd. for $C_{46}H_{32}N_4O_2$ $[M–H]^-$ *m/z* 671.2447, Found *m/z* 671.2444.

(R)-N-(3-(1H-imidazol-1-yl) propyl)-2-(10,15,20-triphenylporphyrin-5-yl) benzamid (C)

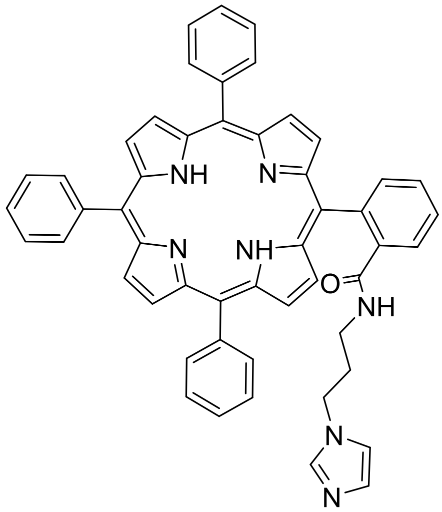

Purple solid, yield = 85%; **^{1}H NMR** (600 MHz, $CDCl_3$) δ 8.94 – 8.82 (m, 8H), 8.30 – 8.26 (m, 2H), 8.22 – 8.16 (m, 4H), 8.13 (d, *J* = 7.1 Hz, 2H), 7.91 (t, *J* = 7.8 Hz, 1H), 7.82 – 7.74 (m, 10H), 5.98 (s, 1H), 5.78 (s, 1H), 4.91 (br, *J* = 6.3 Hz, 1H), 4.37 (s, 1H), 2.15 (q, *J* = 6.2 Hz, 2H), 1.00 (t, *J* = 7.0 Hz, 2H), -0.29 (p, *J* = 6.5 Hz, 2H), -2.68 (s, 2H); **^{13}C NMR** (151 MHz, $CDCl_3$) δ 168.79, 141.93, 141.69, 139.29, 138.94, 135.45, 135.33, 134.72, 134.67, 134.65, 134.58, 129.14, 128.84, 128.79, 128.11, 128.08, 127.26, 127.05, 126.97, 126.93, 126.89, 121.28, 120.96, 117.36, 116.71, 41.79, 35.37, 29.28; **HRMS (ESI):** Calcd. for $C_{51}H_{39}N_7O$ $[M+H]^+$ *m/z* 766.3289, Found *m/z* 766.3290.

Fe porphyrin E

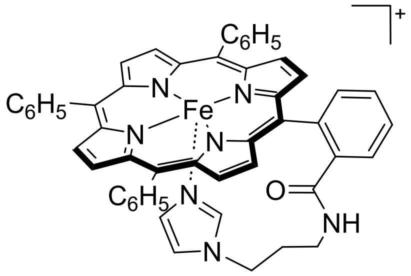

Purple solid, yield = 89%; **HRMS (ESI):** Calcd. for $C_{51}H_{37}N_7OFe$ $[M+H]^+$ *m/z* 819.2404, Found *m/z* 819.2413. The purity of iron porphyrin **E** was determined to be 98.72% by Supercritical Fluid Chromatography (SFC).

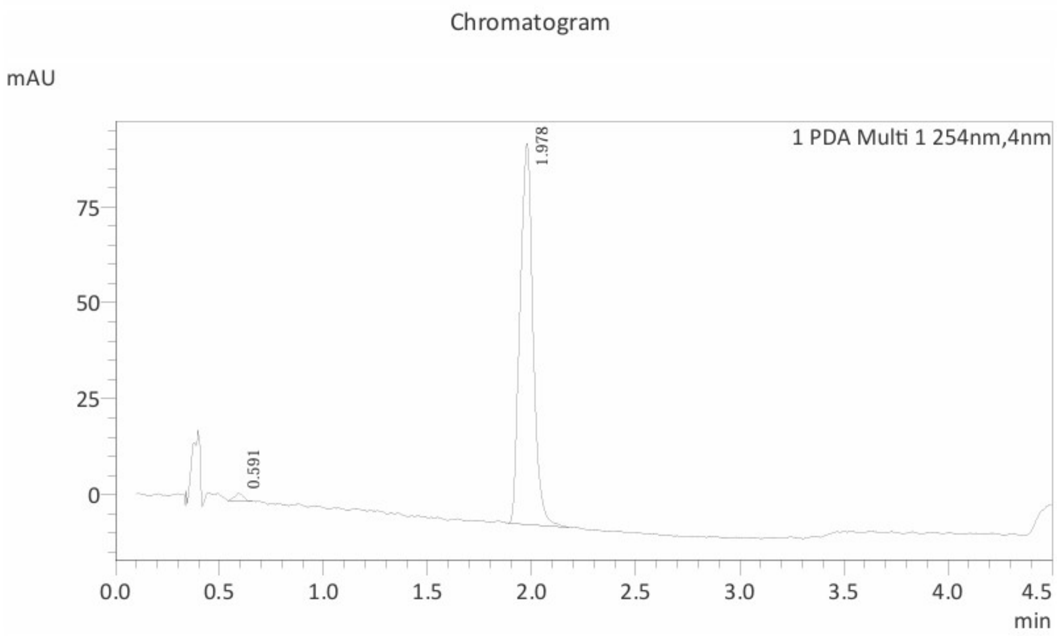

Peak Table

PDA Ch1 254nm

Peak#	Ret. Time	Height	Area	Area%	Resolution(USP)	Tailing Factor	USP Width
1	0.591	1966	5627	1.285	--	0.988	0.084
2	1.978	99206	432426	98.715	13.588	1.096	0.120
Total		101171	438053	100.000			

Co-Solvent: MeOH (0.1% TFA)

Column Name and Size: Torus DIOL 130A 4.6*100 mm, 5 μm

Oven Temperature: 35°C

Total Flow: 4.0000 mL/min

Start Conc. of Pump B: 10.0%

BPR Pressure: 10.00 MPa

PDA Model: SPD-M20A

Lamp: D2

Start Wavelength: 190 nm

End Wavelength: 400 nm

LC Time Program Table:

Time	Module	Command	Value
3.00	Pumps	Pump B Conc.	50
4.00	Pumps	Pump B Conc.	50
4.50	Pumps	Pump B Conc.	10
4.50	Controller	Stop	

Fe porphyrin H

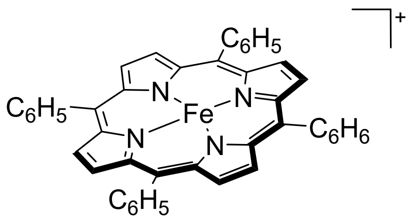

The commercially available iron tetraphenylporphyrin chloride, denoted as **(H)Cl⁻**, was treated with one equivalent of AgOTf to abstract the chloride ion, yielding the product designated as **H**.

3. Characterization of Fe Porphyrins

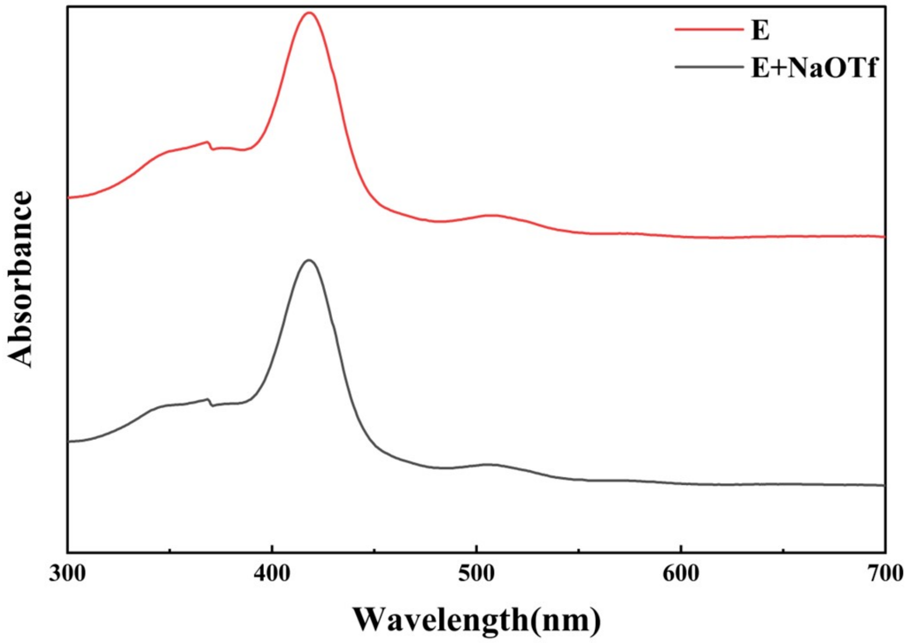

Figure S1. UV/Vis spectra of **E** and **E** added with 10 equivalents of NaOTf in THF.

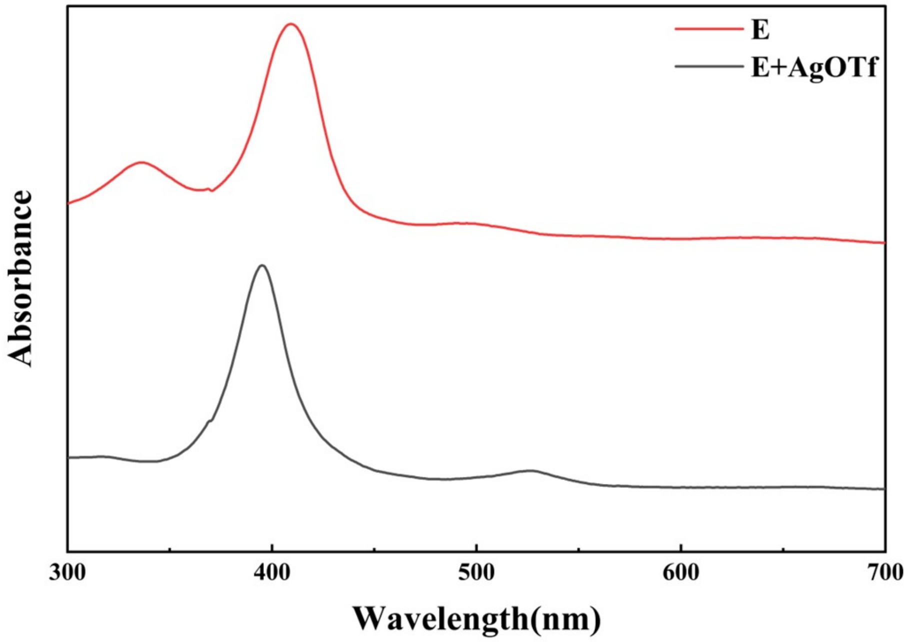

Figure S2. UV/Vis spectra of **E** and **E** added with 30 equivalents of AgOTf in TFE.

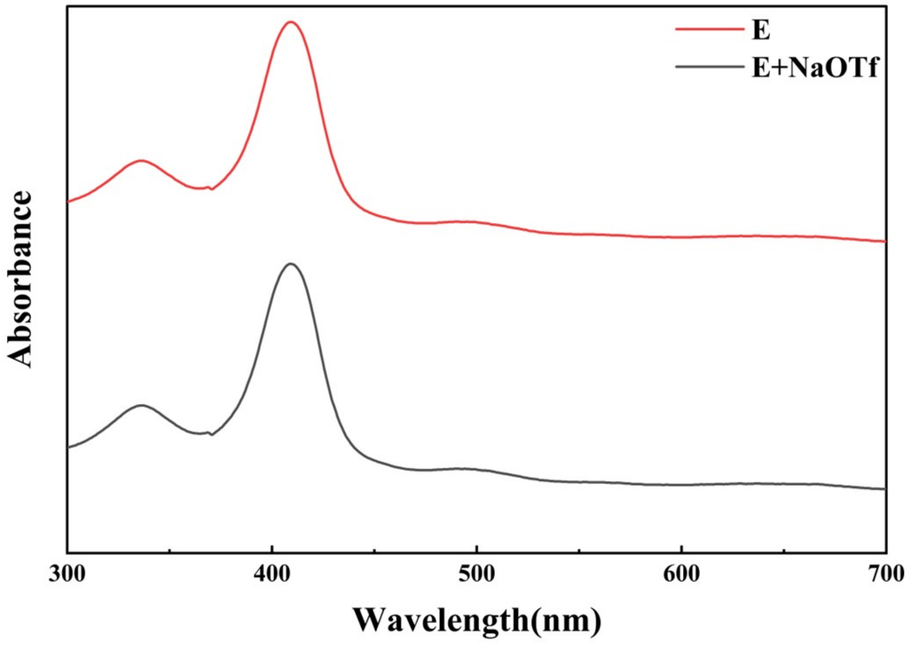

Figure S3. UV/Vis spectra of **E** and **E** added with 30 equivalents of NaOTf in TFE.

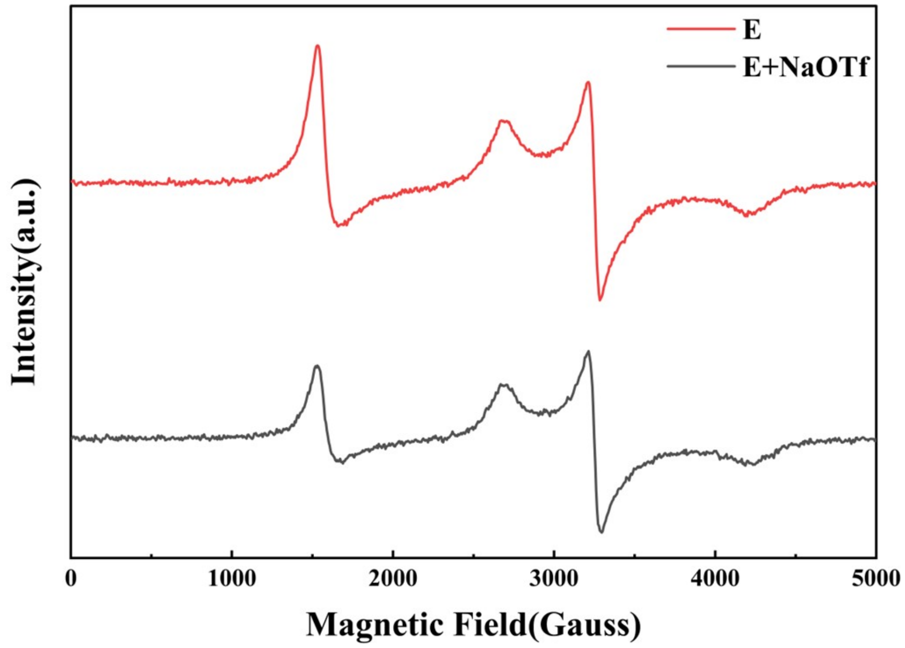

Figure S4. EPR spectra of **E** and **E** added with 10 equivalents of NaOTf in THF measured at 100 K.

4. General Procedure

General procedure for the iron-porphyrin-catalyzed oxidation of *o*-aminophenols and *o*-phenylenediamines[5,6]: A mixture of the substrate to be oxidized (1 mmol) and iron-porphyrin catalyst **E** (1 mol%) in 2,2,2-trifluoroethanol (TFE, 10 mL) was stirred at 60 °C under an air atmosphere. After complete consumption of the starting material (monitored by TLC), the solvent was removed under reduced pressure. The residue was purified by column chromatography on silica gel (eluent: dichloromethane/methanol = 95:5, v/v) to afford the desired product.

The substrate scope of the oxidation using various substituted *o*-aminophenols

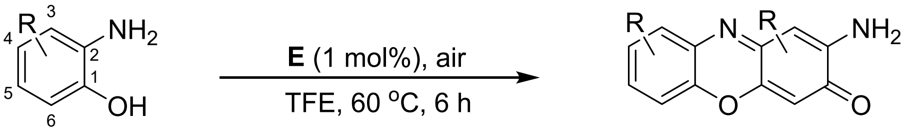

1 **2**

2-amino-*3H*-phenoxazin-3-one (2a)

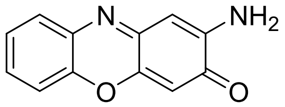

Dark red solid, 6h, yield = 86%; **^{1}H NMR** (600 MHz, DMSO-*d6*) δ 7.70 (dd, *J* = 8.1, 1.3 Hz, 1H), 7.50 – 7.44 (m, 2H), 7.40 – 7.37 (m, 1H), 6.81 (br, 2H), 6.36 (d, *J* = 2.9 Hz, 2H); **^{13}C NMR** (151 MHz, DMSO-*d6*) δ 180.22, 148.88, 148.25, 147.38, 141.93, 133.74, 128.80, 127.98, 125.28, 115.94, 103.43, 98.37; **HRMS (ESI):** Calcd. for $C_{12}H_8N_2O_2$ $[M+H]^+$ *m/z* 213.0659, Found *m/z* 213.0660.

2-amino-1,9-dimethyl-*3H*-phenoxazin-3-one (2b)

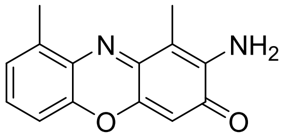

Dark red solid, 6h, yield = 88%; **^{1}H NMR** (600 MHz, DMSO-*d6*) δ 7.35 (t, *J* = 7.8 Hz, 1H), 7.30 – 7.25 (m, 2H), 6.39 (br, 2H), 6.25 (s, 1H), 2.60 (s, 3H), 2.23 (s, 3H); **^{13}C NMR** (151 MHz, DMSO-*d6*) δ 179.65, 148.82, 145.91, 143.75, 141.86, 136.75, 131.69, 128.40, 125.88, 113.36, 105.68, 102.06, 16.42, 9.75; **HRMS (ESI):** Calcd. for $C_{14}H_{12}N_2O_2$ $[M+H]^+$ *m/z* 241.0972, Found *m/z* 241.0973.

2-amino-4,6-dimethyl-*3H*-phenoxazin-3-one (2c)

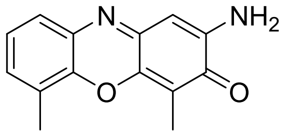

Dark red solid, 6h, yield = 84%; ^{1}**H NMR** (600 MHz, DMSO-*d6*) δ 7.51 (d, *J* = 7.9 Hz, 1H), 7.32 (d, *J* = 7.3 Hz, 1H), 7.26 (t, *J* = 7.7 Hz, 1H), 6.71 (br, 2H), 6.30 (s, 1H), 2.45 (s, 3H), 2.09 (s, 3H); ^{13}**C NMR** (151 MHz, DMSO-*d6*) δ 179.94, 147.78, 146.73, 144.72, 140.53, 133.28, 129.75, 125.59, 124.96, 124.33, 111.23, 97.53, 14.44, 7.57; **HRMS (ESI):** Calcd. for $C_{14}H_{12}N_2O_2$ $[M+H]^+$ *m/z* 241.0972, Found *m/z* 241.0973.

2-amino-1,9-difluoro-*3H*-phenoxazin-3-one (2d)

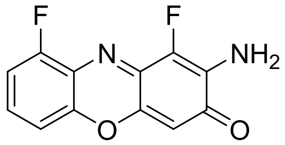

Black solid, 8h, yield = 87%; ^{1}**H NMR** (600 MHz, DMSO-*d6*) δ 7.53 – 7.49 (m, 1H), 7.38 – 7.30 (m, 2H), 6.89 (br, 2H), 6.34 (s, 1H); ^{13}**C NMR** (151 MHz, DMSO-*d6*) δ 179.44 (d, *J* = 9.8 Hz), 156.76 (d, *J* = 255.6 Hz), 146.47 (d, *J* = 6.6 Hz), 143.10 (d, *J* = 3.4 Hz), 139.47 (d, *J* = 15.1 Hz), 137.47 (d, *J* = 243.8 Hz), 133.19 (d, *J* = 4.9 Hz), 129.32 (d, *J* = 9.2 Hz), 123.10 (d, *J* = 14.9 Hz), 112.07 (d, *J* = 3.6 Hz), 111.47 (d, *J* = 18.7 Hz), 101.89; **HRMS (ESI):** Calcd. for $C_{12}H_6F_2N_2O_2$ $[M+H]^+$ *m/z* 249.0471, Found *m/z* 249.0473.

2-amino-4,6-difluoro-*3H*-phenoxazin-3-one (2e)

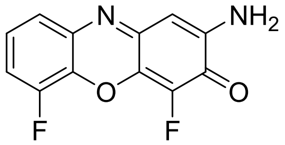

Black solid, 8h, yield = 86%; ^{1}**H NMR** (600 MHz, DMSO-*d6*) δ 7.54 (d, *J* = 8.1 Hz, 1H), 7.47 – 7.43 (m, 1H), 7.39 – 7.35 (m, 1H), 7.09 (br, 2H), 6.31 (s, 1H); ^{13}**C NMR** (151 MHz, DMSO-*d6*) δ 172.36 (d, *J* = 16.1 Hz), 149.33 (d, *J* = 249.2 Hz), 147.39, 146.75 (d, *J* = 4.3 Hz), 138.83 (d, *J* = 253.2 Hz), 135.33, 132.12 (d, *J* = 6.0 Hz), 129.60 (d, *J* = 11.2 Hz), 124.65 (d, *J* = 7.7 Hz), 123.80 (d, *J* = 3.0 Hz), 115.28 (d, *J* = 17.0 Hz), 96.44; **HRMS (ESI):** Calcd. for $C_{12}H_6F_2N_2O_2$ $[M+H]^+$ *m/z* 249.0471, Found *m/z* 249.0473.

2-amino-4,6-dichloro-*3H*-phenoxazin-3-one (2f)

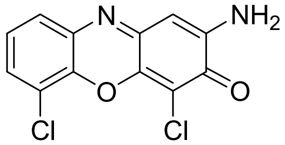

Black solid, 10h, yield = 87%; ^{1}**H NMR** (600 MHz, DMSO-*d6*) δ 7.70 (d, *J* = 8.0 Hz, 1H), 7.64 (d, *J* = 7.8 Hz, 1H), 7.42 (t, *J* = 8.0 Hz, 1H), 7.12 (br, 2H), 6.38 (s, 1H); ^{13}**C NMR** (151 MHz, DMSO-*d6*) δ 174.32, 147.41, 147.23, 143.94, 137.75, 134.97, 128.88, 126.90, 125.73, 119.85, 109.57, 97.54; **HRMS (ESI):** Calcd. for $C_{12}H_6Cl_2N_2O_2$ $[M+H]^+$ *m/z* 280.9880, Found *m/z* 280.9883.

2-amino-4,6-dibromo-3H-phenoxazin-3-one (2g)

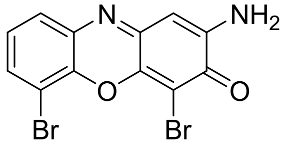

Black solid, 10h, yield = 85%; **^{1}H NMR** (600 MHz, DMSO-*d6*) δ 7.78 (dd, *J* = 7.9, 1.4 Hz, 1H), 7.73 (dd, *J* = 8.0, 1.4 Hz, 1H), 7.37 (t, *J* = 8.0 Hz, 1H), 7.12 (br, 2H), 6.39 (s, 1H); **^{13}C NMR** (151 MHz, DMSO-*d6*) δ 174.50, 147.64, 147.14, 146.12, 139.06, 135.09, 131.85, 127.40, 126.41, 108.84, 101.23, 97.69; **HRMS (ESI):** Calcd. for $C_{12}H_6Br_2N_2O_2$ $[M+H]^+$ *m/z* 370.8849, Found *m/z* 370.8849.

2-amino-4a,7-dimethyl-4,4a-dihydro-3H-phenoxazin-3-one (2h)

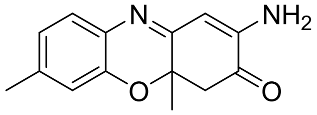

Yellow solid, 6h, yield = 86%; **^{1}H NMR** (600 MHz, DMSO-*d6*) δ 7.09 (d, *J* = 7.9 Hz, 1H), 6.80 (dd, *J* = 8.3, 1.6 Hz, 1H), 6.72 (d, *J* = 1.8 Hz, 1H), 6.38 (br, 2H), 6.04 (s, 1H), 3.20 (d, *J* = 15.8 Hz, 1H), 2.98 (d, *J* = 15.8 Hz, 1H), 2.26 (s, 3H), 1.09 (s, 3H); **^{13}C NMR** (151 MHz, DMSO-*d6*) δ 191.58, 160.87, 146.44, 143.82, 136.58, 132.68, 125.47, 123.03, 116.54, 105.88, 70.94, 49.20, 22.02, 20.92; **HRMS (ESI):** Calcd. for $C_{14}H_{14}N_2O_2$ $[M+H]^+$ *m/z* 243.1129, Found *m/z* 243.1130.

2-amino-7-fluoro-*3H*-phenoxazin-3-one (2i)

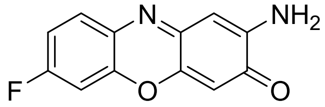

Black solid, 8h, yield = 80%; **^{1}H NMR** (600 MHz, DMSO-*d6*) δ 7.75 (dd, *J* = 8.9, 6.1 Hz, 1H), 7.49 (dd, *J* = 9.2, 2.8 Hz, 1H), 7.29 – 7.26 (m, 1H), 6.78 (br, 2H), 6.37 (s, 1H), 6.34 (s, 1H); **^{13}C NMR** (151 MHz, DMSO-*d6*) δ 180.19, 160.97 (d, *J* = 247.9 Hz), 148.42, 147.59 (d, *J* = 3.0 Hz), 147.22, 142.54 (d, *J* = 13.7 Hz), 130.84 (d, *J* = 2.7 Hz), 129.46 (d, *J* = 10.0 Hz), 113.00 (d, *J* = 23.5 Hz), 103.77, 103.44 (d, *J* = 27.5 Hz), 98.37; **HRMS (ESI):** Calcd. for $C_{12}H_7FN_2O_2$ $[M+H]^+$ *m/z* 231.0565, Found *m/z* 231.0565.

2-amino-7-chloro-*3H*-phenoxazin-3-one (2j)

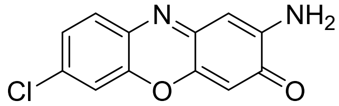

Black solid, 10h, yield = 83%; **^{1}H NMR** (600 MHz, DMSO-*d6*) δ 7.69 (d, *J* = 8.6 Hz, 1H), 7.65 (d, *J* = 2.2 Hz, 1H), 7.42 (dd, *J* = 8.6, 2.3 Hz, 1H), 6.89 (br, 2H), 6.35 (s, 1H), 6.34 (s, 1H); **^{13}C NMR** (151 MHz, DMSO-*d6*) δ 180.27, 148.43, 148.37, 147.54, 142.25, 132.77, 131.98, 129.06, 125.45, 115.98, 103.76, 98.30; **HRMS (ESI):** Calcd. for $C_{12}H_7ClN_2O_2$ $[M+H]^+$ *m/z* 247.0269, Found *m/z* 247.0268.

2-amino-7-bromo-3H-phenoxazin-3-one (2k)

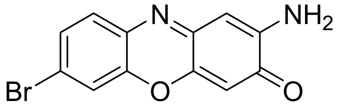

Dark red solid, 10h, yield = 81%; **^{1}H NMR** (600 MHz, DMSO-*d6*) δ 7.76 (d, *J* = 2.1 Hz, 1H), 7.61 (d, *J* = 8.6 Hz, 1H), 7.53 (dd, *J* = 8.5, 2.1 Hz, 1H), 6.90 (br, 2H), 6.34 (s, 1H), 6.33 (s, 1H); **^{13}C NMR** (151 MHz, DMSO-*d6*) δ 180.28, 148.42, 148.38, 147.57, 142.32, 133.02, 129.25, 128.26, 120.16, 118.78, 103.74, 98.32; **HRMS (ESI):** Calcd. for $C_{12}H_7BrN_2O_2$ $[M+H]^+$ *m/z* 290.9764, Found *m/z* 290.9762.

2-amino-8-methoxy-*3H*-phenoxazin-3-one (2l)

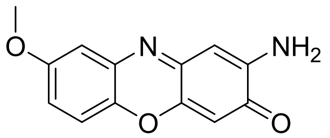

Dark red solid, 4h, yield = 41%; **^{1}H NMR** (600 MHz, DMSO-*d6*) δ 7.44 (d, *J* = 9.0 Hz, 1H), 7.22 (d, *J* = 3.0 Hz, 1H), 7.06 (dd, *J* = 9.1, 3.0 Hz, 1H), 6.83 (br, 2H), 6.35 (s, 1H), 6.32 (s, 1H), 3.83 (s, 3H); **^{13}C NMR** (151 MHz, DMSO-*d6*) δ 179.85, 156.46, 149.01, 148.29, 147.57, 136.37, 134.39, 116.79, 116.60, 109.87, 102.93, 98.12, 55.74; **HRMS (ESI):** Calcd. for $C_{13}H_{10}N_2O_3$ $[M+H]^+$ *m/z* 243.0765, Found *m/z* 243.0764.

2-amino-8-fluoro-3H-phenoxazin-3-one (2m)

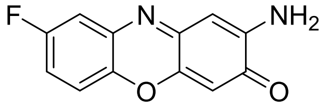

Black solid, 8h, yield = 81%; **^{1}H NMR** (600 MHz, DMSO-*d6*) δ 7.56 – 7.52 (m, 2H), 7.34 – 7.30 (m, 1H), 6.98 (br, 2H), 6.36 (s, 1H), 6.34 (s, 1H); **^{13}C NMR** (151 MHz, DMSO-*d6*) δ 180.16, 158.68 (d, *J* = 240.8 Hz), 148.98, 148.95, 147.88, 138.51, 134.48 (d, *J* = 12.3 Hz), 117.29 (d, *J* = 9.6 Hz), 115.68 (d, *J* = 24.9 Hz), 112.96 (d, *J* = 23.5 Hz), 103.41, 97.92; **HRMS (ESI):** Calcd. for $C_{12}H_7FN_2O_2$ $[M+H]^+$ *m/z* 231.0565, Found *m/z* 231.0564.

2-amino-8-chloro-*3H*-phenoxazin-3-one (2n)

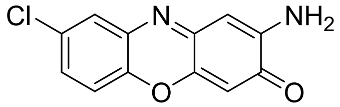

Black solid, 10h, yield = 58%; **^{1}H NMR** (600 MHz, DMSO-*d6*) δ 7.73 (d, *J* = 2.5 Hz, 1H), 7.52 (d, *J* = 8.8 Hz, 1H), 7.46 (dd, *J* = 8.8, 2.5 Hz, 1H), 6.98 (br, 2H), 6.37 (s, 1H), 6.34 (s, 1H); **^{13}C NMR** (151 MHz, DMSO-*d6*) δ 180.24, 149.08, 148.80, 147.87, 140.78, 134.68, 128.70, 127.99, 126.68, 117.59, 103.67, 98.04; **HRMS (ESI):** Calcd. for $C_{12}H_7ClN_2O_2$ $[M+H]^+$ *m/z* 247.0269, Found *m/z* 247.0269.

2-amino-8-bromo-3H-phenoxazin-3-one (2o)

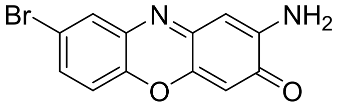

Dark red solid, 10h, yield = 52%; **^{1}H NMR** (600 MHz, DMSO-*d6*) δ 7.86 (d, *J* = 2.4 Hz, 1H), 7.58 (dd, *J* = 8.7, 2.4 Hz, 1H), 7.46 (d, *J* = 8.7 Hz, 1H), 6.98 (br, 2H), 6.37 (s, 1H), 6.34 (s, 1H); **^{13}C NMR** (151 MHz, DMSO-*d6*) δ 180.23, 149.03, 148.76, 147.85, 141.20, 135.07, 130.76, 129.65, 117.91, 116.48, 103.70, 98.07; **HRMS (ESI):** Calcd. for $C_{12}H_7BrN_2O_2$ $[M+H]^+$ *m/z* 290.9764, Found *m/z* 290.9761.

phenazine-2,3-diamine (4a)

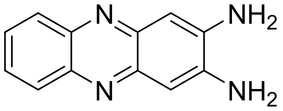

Brown solid, 8h, yield = 73%; **^{1}H NMR** (600 MHz, DMSO-*d6*) δ 7.90 (dd, *J* = 6.5, 3.4 Hz, 2H), 7.54 (dd, *J* = 6.6, 3.4 Hz, 2H), 6.92 (s, 2H), 6.25 (br, 4H); **^{13}C NMR** (151 MHz, DMSO-*d6*) δ 144.10, 142.07, 140.29, 127.88, 126.46, 102.18; **HRMS (ESI):** Calcd. for $C_{12}H_{10}N_4$ $[M+H]^+$ *m/z* 211.0979, Found *m/z* 211.0979.

7-methoxyphenazine-2,3-diamine (4b)

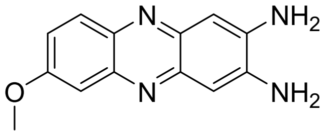

Brown solid, 8h, yield = 72%; **^{1}H NMR** (600 MHz, DMSO-*d6*) δ 7.79 (d, *J* = 9.1 Hz, 1H), 7.25 – 7.21 (m, 2H), 6.89 (d, *J* = 9.8 Hz, 2H), 6.15 (br, 2H), 5.99 (br, 2H), 3.90 (s, 3H); **^{13}C NMR** (151 MHz, DMSO-*d6*) δ 158.00, 143.92, 142.56, 141.76, 141.56, 140.24, 136.58, 129.05, 120.29, 104.96, 103.00, 102.22, 55.44; **HRMS (ESI):** Calcd. for $C_{13}H_{12}N_4O$ $[M+H]^+$ *m/z* 241.1084, Found *m/z* 241.1085.

7-fluorophenazine-2,3-diamine (4c)

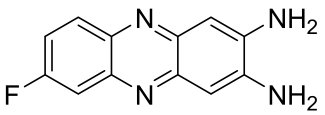

Brown solid, 8h, yield = 70%; **^{1}H NMR** (600 MHz, DMSO-*d6*) δ 7.95 (dd, *J* = 9.3, 6.2 Hz, 1H), 7.61 – 7.59 (m, 1H), 7.48 – 7.44 (m, 1H), 6.89 (d, *J* = 9.9 Hz, 2H), 6.38 (br, 2H), 6.25 (br, 2H); **^{13}C NMR** (151 MHz, DMSO-*d6*) δ 160.25 (d, *J* = 245.3 Hz), 144.81, 143.85, 142.41, 141.67, 140.41 (d, *J* = 13.1 Hz), 137.52, 130.14 (d, *J* = 10.3 Hz), 116.82 (d, *J* = 26.7 Hz), 110.29 (d, *J* = 20.5 Hz), 102.24, 101.62; **HRMS (ESI):** Calcd. for $C_{12}H_9FN_4$ $[M+H]^+$ *m/z* 229.0884, Found *m/z* 229.0883.

7-chlorophenazine-2,3-diamine (4d)

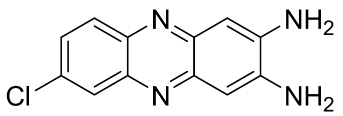

Brown solid, 12h, yield = 63%; ^{1}**H NMR** (600 MHz, DMSO-*d6*) δ 7.92 (d, *J* = 2.4 Hz, 1H), 7.90 (d, *J* = 9.0 Hz, 1H), 7.52 (dd, *J* = 9.0, 2.4 Hz, 1H), 6.89 (d, *J* = 5.6 Hz, 2H), 6.41 (br, 2H), 6.36 (br, 2H); ^{13}**C NMR** (151 MHz, DMSO-*d6*) δ 144.85, 144.43, 142.49, 142.25, 140.35, 138.72, 130.43, 129.66, 126.80, 126.15, 102.02, 101.75; **HRMS (ESI):** Calcd. for $C_{12}H_9ClN_4$ $[M+H]^+$ *m/z* 245.0589, Found *m/z* 245.0590.

7-bromophenazine-2,3-diamine (4e)

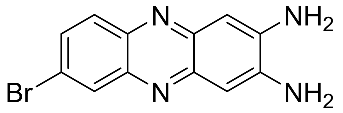

Brown solid, 12h, yield = 61%; ^{1}**H NMR** (600 MHz, DMSO-*d6*) δ 8.08 (d, *J* = 2.2 Hz, 1H), 7.83 (d, *J* = 9.0 Hz, 1H), 7.62 (dd, *J* = 9.0, 2.3 Hz, 1H), 6.89 (d, *J* = 3.7 Hz, 2H), 6.41 (br, 2H), 6.37 (br, 2H); ^{13}**C NMR** (151 MHz, DMSO-*d6*) δ 144.88, 144.52, 142.43, 142.32, 140.81, 138.90, 129.76, 129.47, 129.24, 118.94, 102.02, 101.79; **HRMS (ESI):** Calcd. for $C_{12}H_9BrN_4$ $[M+H]^+$ *m/z* 289.0084, Found *m/z* 289.0084.

1,9-dimethylphenazine-2,3-diamine (4f-1)

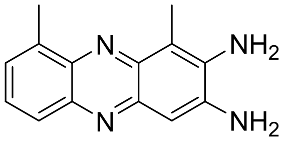

Brown solid, 8h, yield = 34%; ^{1}**H NMR** (600 MHz, DMSO-*d6*) δ 7.79 (d, *J* = 8.3 Hz, 1H), 7.45 – 7.39 (m, 2H), 6.92 (s, 1H), 6.16 (br, 2H), 5.81 (br, 2H), 2.72 (s, 3H), 2.53 (s, 3H); ^{13}**C NMR** (151 MHz, DMSO-*d6*) δ 143.52, 140.99, 140.11, 139.96, 139.56, 139.25, 135.17, 126.40, 126.19, 125.94, 107.97, 101.23, 17.48, 10.55; **HRMS (ESI):** Calcd. for $C_{14}H_{14}N_4[M+H]^+$ *m/z* 239.1292, Found *m/z* 239.1287.

1,6-dimethylphenazine-2,3-diamine (4f-2)

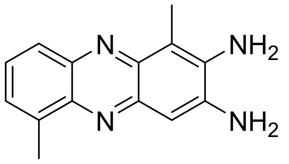

Brown solid, 8h, yield = 38%; ^{1}**H NMR** (600 MHz, DMSO-*d6*) δ 7.74 (s, 1H), 7.46 – 7.39 (m, 2H), 6.86 (s, 1H), 6.18 (br, 2H), 5.81 (br, 2H), 2.78 (s, 3H), 2.57 (s, 3H); ^{13}**C NMR** (151 MHz, DMSO-*d6*) δ 143.64, 141.56, 139.93, 139.91, 139.37, 138.68, 135.73, 126.28, 125.84, 125.66, 108.42, 100.52, 17.21, 10.45; **HRMS (ESI):** Calcd. for $C_{14}H_{14}N_4[M+H]^+$ *m/z* 239.1292, Found *m/z* 239.1288.

1,9-difluorophenazine-2,3-diamine (4g-1)

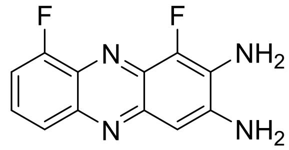

Brown solid, 12h, yield = 25%; **^{1}H NMR** (600 MHz, DMSO-*d6*) δ 7.83 (d, *J* = 8.5 Hz, 1H), 7.57 – 7.54 (m, 1H), 7.43 – 7.40 (m, 1H), 6.86 (s, 1H), 6.64 (br, 2H), 6.36 (br, 2H); **^{13}C NMR** (151 MHz, DMSO-*d6*) δ 156.28 (d, *J* = 256.3 Hz), 144.68 (d, *J* = 7.3 Hz), 140.43, 139.48, 138.33 (d, *J* = 238.5 Hz), 131.93 (d, *J* = 9.8 Hz), 131.14 (d, *J* = 12.1 Hz), 130.44 (d, *J* = 12.3 Hz), 125.74 (d, *J* = 8.6 Hz), 124.27 (d, *J* = 4.0 Hz), 110.66 (d, *J* = 18.2 Hz), 98.11; **HRMS (ESI):** Calcd. for $C_{12}H_8F_2N_4$ $[M+H]^+$ *m/z* 247.0790, Found *m/z* 247.0789.

1,6-difluorophenazine-2,3-diamine (4g-2)

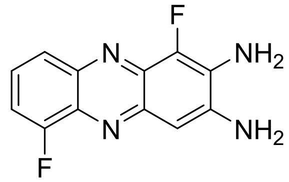

Brown solid, 12h, yield = 48%; **^{1}H NMR** (600 MHz, DMSO-*d6*) δ 7.76 (d, *J* = 8.6 Hz, 1H), 7.56 (m, 1H), 7.41 (m, 1H), 6.83 (s, 1H), 6.66 (br, 2H), 6.35 (br, 2H); **^{13}C NMR** (151 MHz, DMSO-*d6*) δ 156.60 (d, *J* = 256.8 Hz), 144.86 (d, *J* = 7.4 Hz), 141.52, 140.23, 138.63 (d, *J* = 238.9 Hz), 131.21 (d, *J* = 9.8 Hz), 130.33 (d, *J* = 12.5 Hz), 130.08 (d, *J* = 12.2 Hz), 126.27 (d, *J* = 8.5 Hz), 123.98 (d, *J* = 4.0 Hz), 110.29 (d, *J* = 18.4 Hz), 97.82; **HRMS (ESI):** Calcd. for $C_{12}H_8F_2N_4$ $[M+H]^+$ *m/z* 247.0790, Found *m/z* 247.0790.

1,9-dichlorophenazine-2,3-diamine (4h-1)

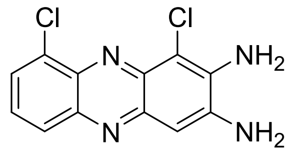

Brown solid, 12h, yield = 37%; **^{1}H NMR** (600 MHz, DMSO-*d6*) δ 7.98 (dd, *J* = 8.6, 1.3 Hz, 1H), 7.79 (dd, *J* = 7.4, 1.2 Hz, 1H), 7.57 (dd, *J* = 8.5, 7.4 Hz, 1H), 6.99 (s, 1H), 6.71 – 7.70 (br, 4H); **^{13}C NMR** (151 MHz, DMSO-*d6*) δ 144.13, 141.37, 140.55, 140.33, 137.73, 136.63, 130.36, 127.61, 127.05, 126.51, 104.56, 100.82; **HRMS (ESI):** Calcd. for $C_{12}H_8Cl_2N_4$$[M+H]^+$ *m/z* 279.0199, Found *m/z* 279.0200.

1,6-dichlorophenazine-2,3-diamine (4h-2)

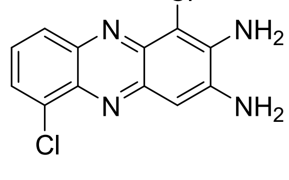

Brown solid, 12h, yield = 39%; **^{1}H NMR** (600 MHz, DMSO-*d6*) δ 7.92 (dd, *J* = 8.5, 1.3 Hz, 1H), 7.78 (dd, *J* = 7.4, 1.3 Hz, 1H), 7.58 (dd, *J* = 8.5, 7.4 Hz, 1H), 6.95 (s, 1H), 6.73 (br, 2H), 6.68 (br, 2H); **^{13}C NMR** (151 MHz, DMSO-*d6*) δ 144.09, 141.65, 140.94, 140.60, 137.38, 136.03, 130.90,

127.11, 126.91, 126.69, 104.93, 100.41; **HRMS (ESI):** Calcd. for $C_{12}H_8Cl_2N_4[M+H]^+$ *m/z* 279.0199, Found *m/z* 279.0202.

1,9-dibromophenazine-2,3-diamine (4i-1)

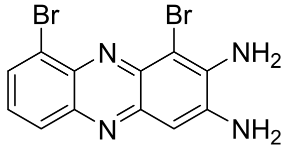

Brown solid, 12, yield = 38%; **^{1}H NMR** (600 MHz, DMSO-*d6*) δ 8.00 (m, 2H), 7.51 (dd, *J* = 8.4, 7.4 Hz, 1H), 7.01 (s, 1H), 6.72 – 6.69 (br, 4H); **^{13}C NMR** (151 MHz, DMSO-*d6*) δ 144.11, 141.99, 141.85, 140.44, 138.57, 137.50, 130.57, 128.22, 127.21, 121.78, 101.32, 97.03; **HRMS (ESI):** Calcd. for $C_{12}H_8Br_2N_4[M+H]^+$ *m/z* 368.9168, Found *m/z* 368.9168.

1,6-dibromophenazine-2,3-diamine (4i-2)

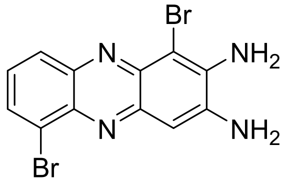

Brown solid, 12h, yield = 36%; **^{1}H NMR** (600 MHz, DMSO-*d6*) δ 7.99 – 7.95 (m, 2H), 7.52 (dd, *J* = 8.5, 7.3 Hz, 1H), 6.97 (s, 1H), 6.70 (br, 4H); **^{13}C NMR** (151 MHz, DMSO-*d6*) δ 144.06, 142.04, 141.95, 140.99, 138.45, 137.05, 130.16, 127.67, 127.61, 122.44, 100.88, 97.47. **HRMS (ESI):** Calcd. for $C_{12}H_8Br_2N_4[M+H]^+$ *m/z* 368.9168, Found *m/z* 368.9168.

5. References

[1] Hiroto, Y. Miyake and H. Shinokubo, *Chemical Reviews*, 2016, **117**, 2910–3043.

[2] M. O. Senge, N. N. Sergeeva and K. J. Hale, *Chemical Society Reviews*, 2021, **50**, 4730–4789.

[3] M. Chen, H. Li, C. Liu, J. Liu, Y. Feng, A. G. H. Wee and B. Zhang, *Coordination Chemistry Reviews*, 2021, **435**, 213778.

[4] L. Xie, X. Zhang, B. Zhao, P. Li, J. Qi, X. Guo, B. Wang, H. Lei, W. Zhang, U. Apfel and R. Cao, *Angew Chem Int Ed*, 2021, 7576–7581.

[5] J. Zhou, Z.-Y. Ma, C. Shonhe, S.-H. Ji and Y.-R. Cai, *Green Chem.*, 2021, **23**, 8566–8570.

[6] D. Sharma, N. Kotwal and P. Chauhan, *Org. Lett.*, 2023, **25**, 3772–3777.

6. ^{1}H and ^{13}C NMR spectra

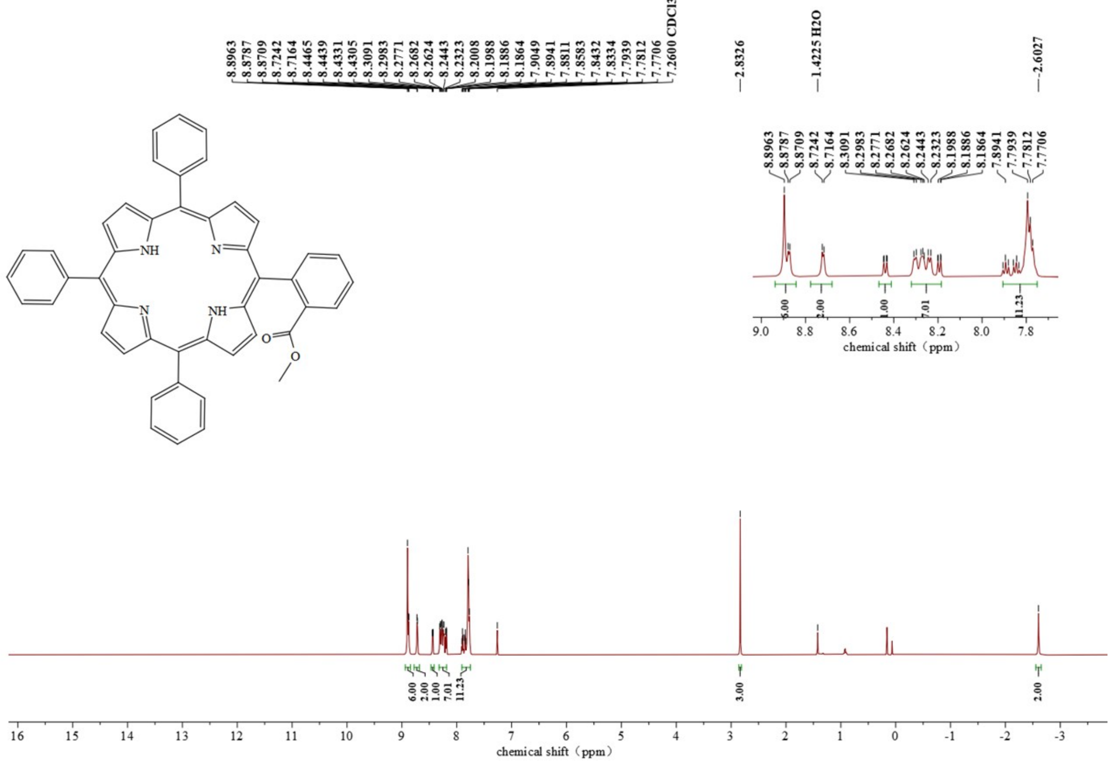
8.8963
8.8787
8.8709
8.7242
8.7164
8.4465
8.4439
8.4331
8.4305
8.3091
8.2983
8.2771
8.2682
8.2624
8.2443
8.2323
8.2008
8.1988
8.1886
8.1864
7.9049
7.8941
7.8811
7.8583
7.8432
7.8334
7.7939
7.7812
7.7706
7.2600 CDCl3
2.8326
1.4225 H2O
-2.6027
NH
N
N
NH
O
O
chemical shift (ppm)

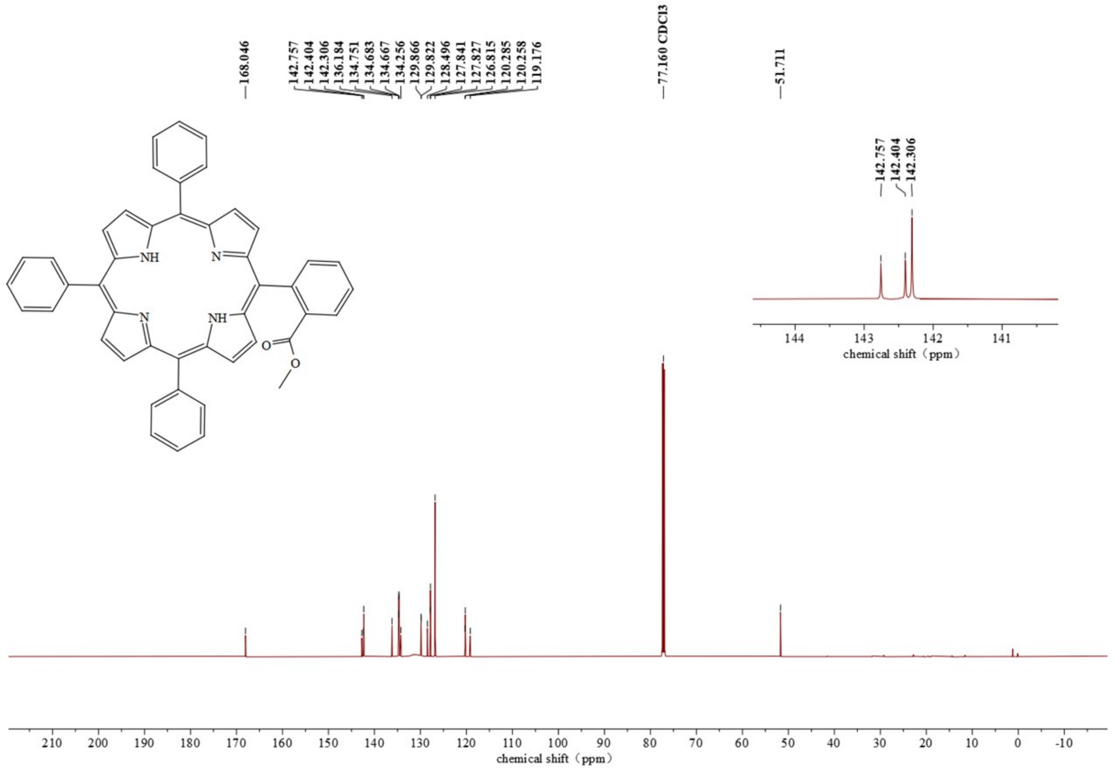
168.046
142.757
142.404
142.306
136.184
134.751
134.683
134.667
134.256
129.866
129.822
128.496
127.841
127.827
126.815
120.285
120.258
119.176
77.160 CDCl3
51.711
NH
N
N
NH
O
O
chemical shift (ppm)

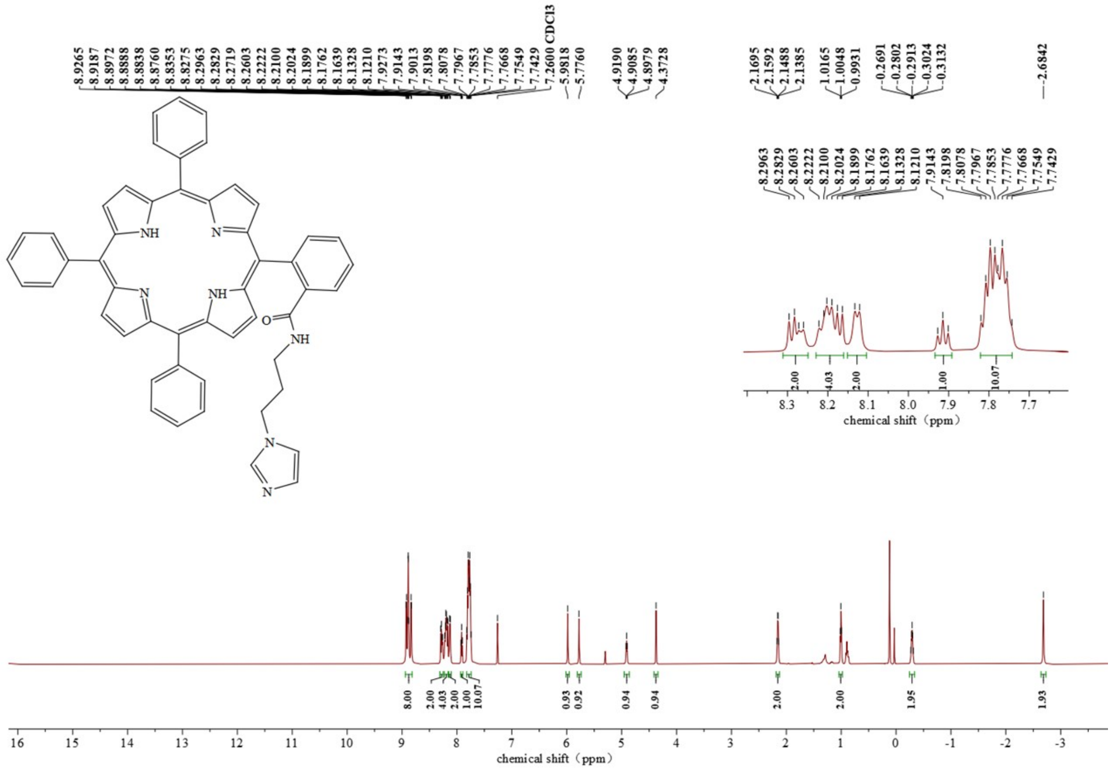
8.9265 8.9187 8.8972 8.8888 8.8838 8.8760 8.8353 8.8275 8.2963 8.2829 8.2719 8.2603 8.2222 8.2100 8.2024 8.1899 8.1762 8.1639 8.1328 8.1210 7.9273 7.9143 7.9013 7.8198 7.8078 7.7967 7.7853 7.7776 7.7668 7.7549 7.7429 7.2600 CDCl3 5.9818 5.7760
4.9190 4.9085 4.8979 4.3728
2.1695 2.1592 2.1488 2.1385 1.0165 1.0048 0.9931 -0.2691 -0.2802 -0.2913 -0.3024 -0.3132
-2.6842
NH
N
NH
N
O
NH
N
N
8.2963 8.2829 8.2603 8.2222 8.2100 8.2024 8.1899 8.1762 8.1639 8.1328 8.1210 7.9143 7.8198 7.8078 7.7967 7.7853 7.7776 7.7668 7.7549 7.7429
2.00 4.03 2.00 1.00 10.07
8.3 8.2 8.1 8.0 7.9 7.8 7.7
chemical shift (ppm)
8.00 2.00 4.03 2.00 1.00 10.07 0.93 0.92 0.94 0.94 2.00 2.00 1.95 1.93
16 15 14 13 12 11 10 9 8 7 6 5 4 3 2 1 0 -1 -2 -3
chemical shift (ppm)

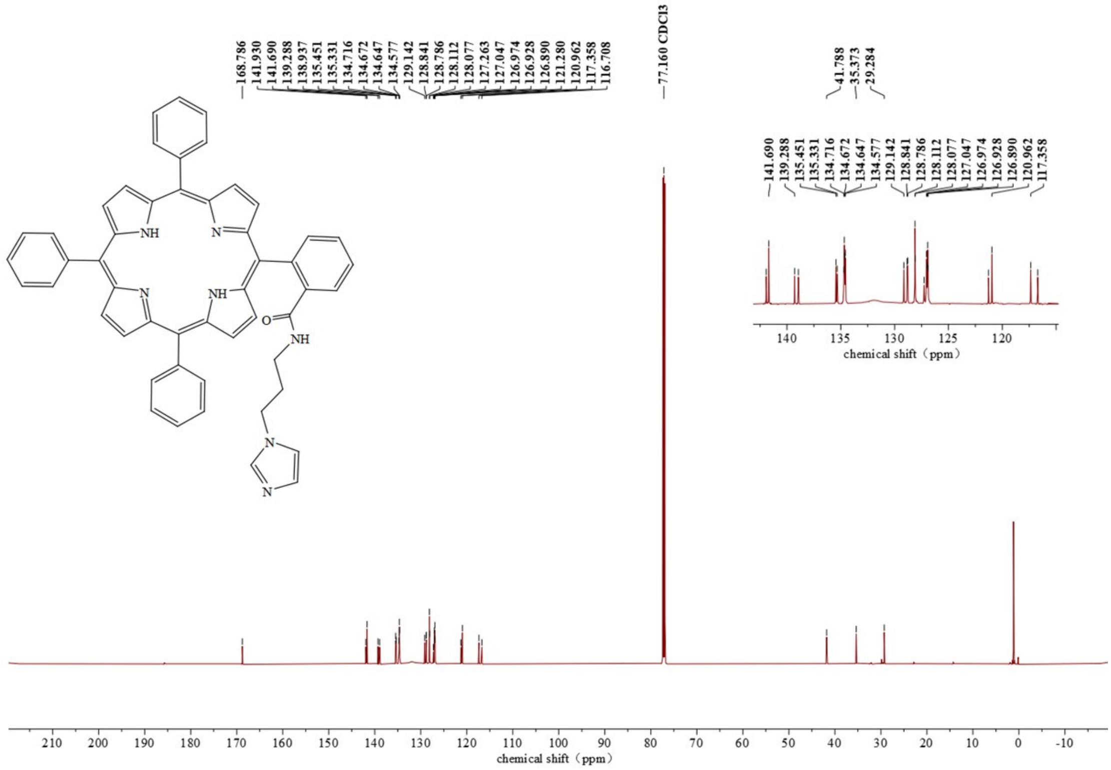
168.786 141.930 141.690 139.288 138.937 135.451 135.331 134.716 134.672 134.647 134.577 129.142 128.841 128.786 128.112 128.077 127.263 127.047 126.974 126.928 126.890 121.280 120.962 117.358 116.708
77.160 CDCl3
41.788 35.373 29.284
NH
N
NH
N
O
NH
N
N
141.690 139.288 135.451 135.331 134.716 134.672 134.647 134.577 129.142 128.841 128.786 128.112 128.077 127.047 126.974 126.928 126.890 120.962 117.358
140 135 130 125 120
chemical shift (ppm)
210 200 190 180 170 160 150 140 130 120 110 100 90 80 70 60 50 40 30 20 10 0 -10
chemical shift (ppm)

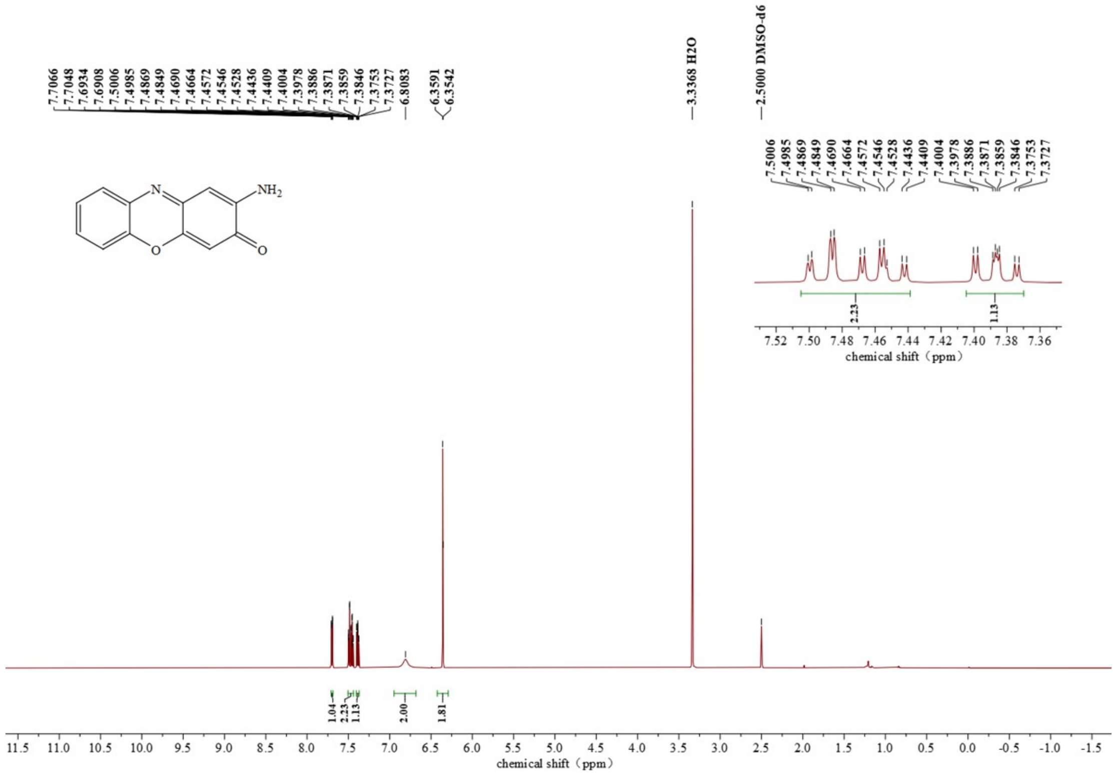
7.7066
7.7048
7.6934
7.6908
7.5006
7.4985
7.4869
7.4849
7.4690
7.4664
7.4572
7.4546
7.4528
7.4436
7.4409
7.4004
7.3978
7.3886
7.3871
7.3859
7.3846
7.3753
7.3727
6.8083
6.3591
6.3542
3.3368 H2O
2.5000 DMSO-d6
NH2
N
O
O
2.23
1.13
7.52 7.50 7.48 7.46 7.44 7.42 7.40 7.38 7.36
chemical shift (ppm)
1.04
2.23
1.13
2.00
1.81
11.5 11.0 10.5 10.0 9.5 9.0 8.5 8.0 7.5 7.0 6.5 6.0 5.5 5.0 4.5 4.0 3.5 3.0 2.5 2.0 1.5 1.0 0.5 0.0 -0.5 -1.0 -1.5
chemical shift (ppm)

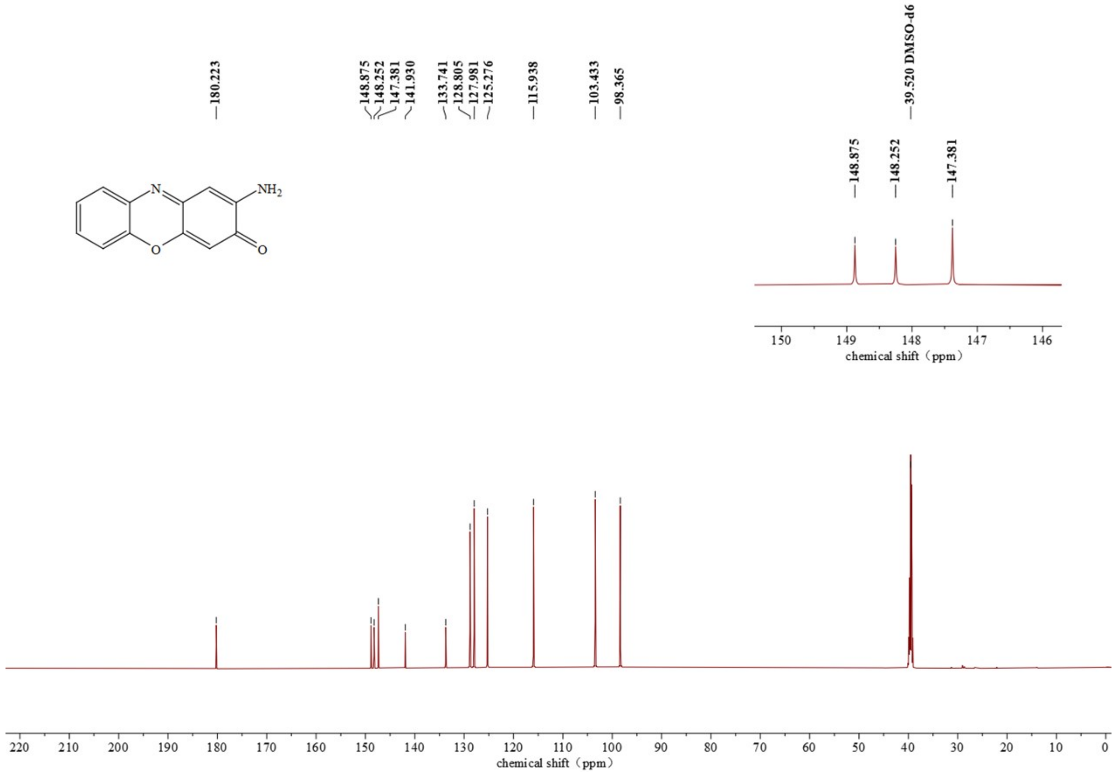
180.223
148.875
148.252
147.381
141.930
133.741
128.805
127.981
125.276
115.938
103.433
98.365
39.520 DMSO-d6
NH2
N
O
O
148.875
148.252
147.381
150 149 148 147 146
chemical shift (ppm)
220 210 200 190 180 170 160 150 140 130 120 110 100 90 80 70 60 50 40 30 20 10 0
chemical shift (ppm)

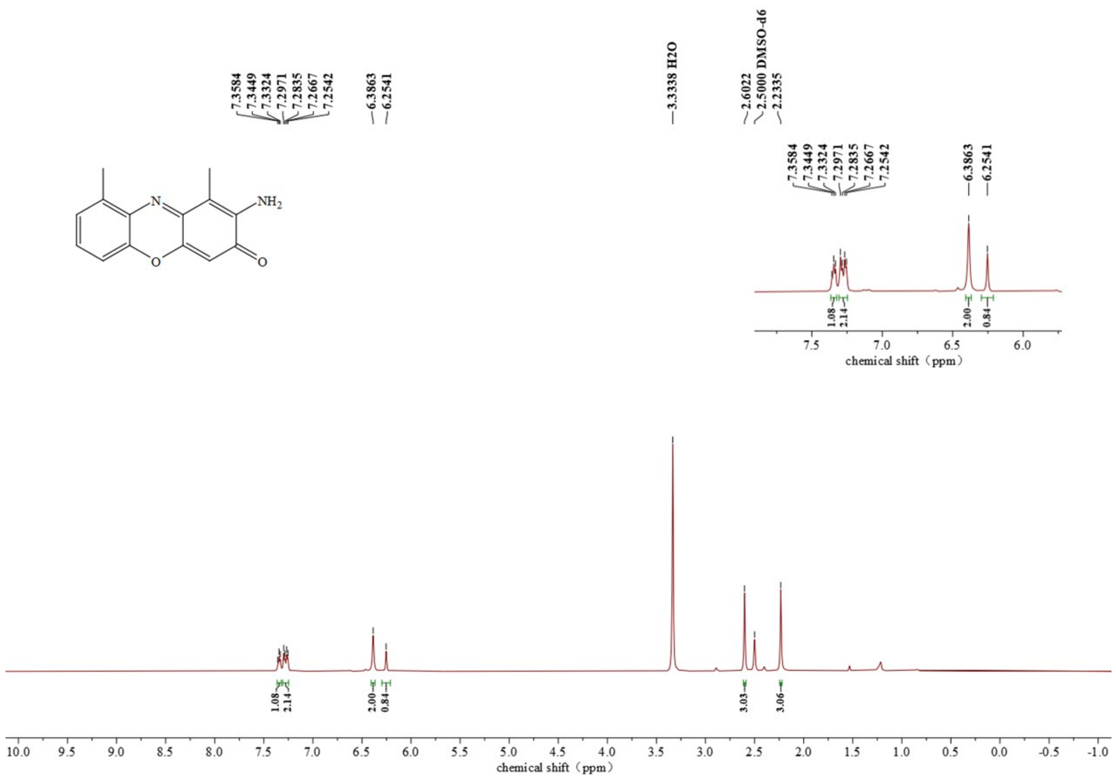
7.3584
7.3449
7.3324
7.2971
7.2835
7.2667
7.2542
6.3863
6.2541
3.3338 H2O
2.6022
2.5000 DMSO-d6
2.2335
NH2
N
O
O
1.08
2.14
2.00
0.84
3.03
3.06
chemical shift（ppm）

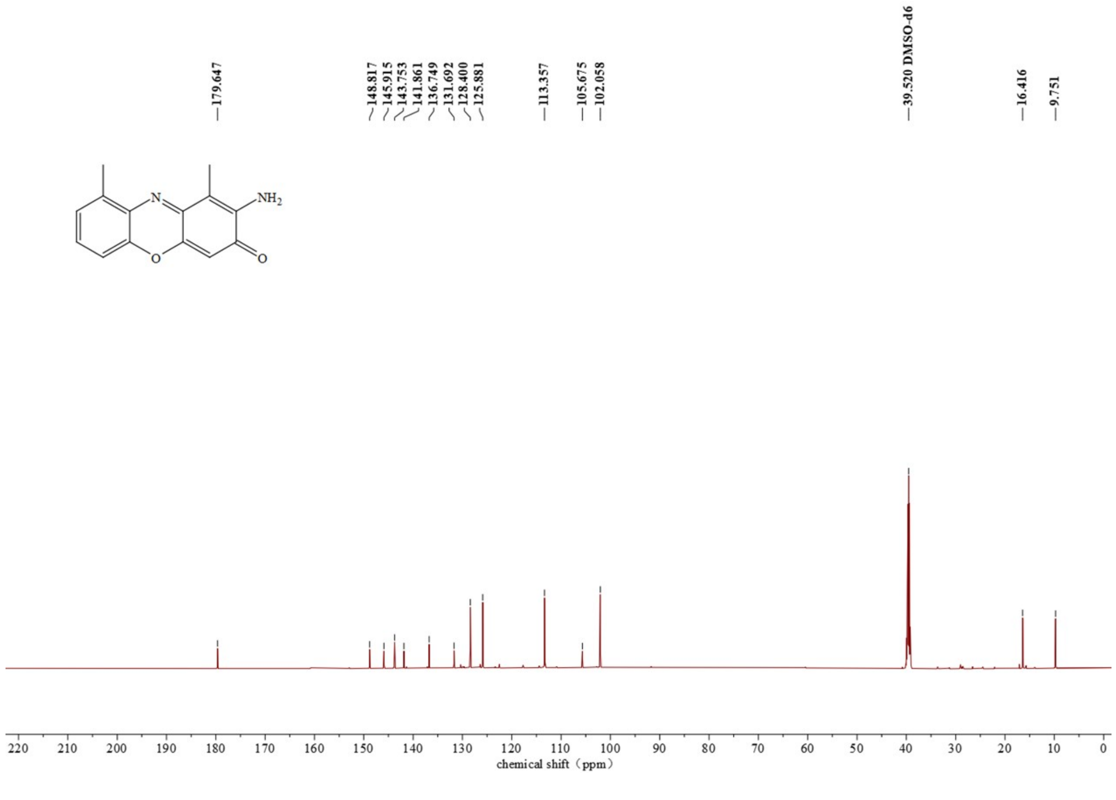
179.647
148.817
145.915
143.753
141.861
136.749
131.692
128.400
125.881
113.357
105.675
102.058
39.520 DMSO-d6
16.416
9.751
NH2
N
O
O
chemical shift（ppm）

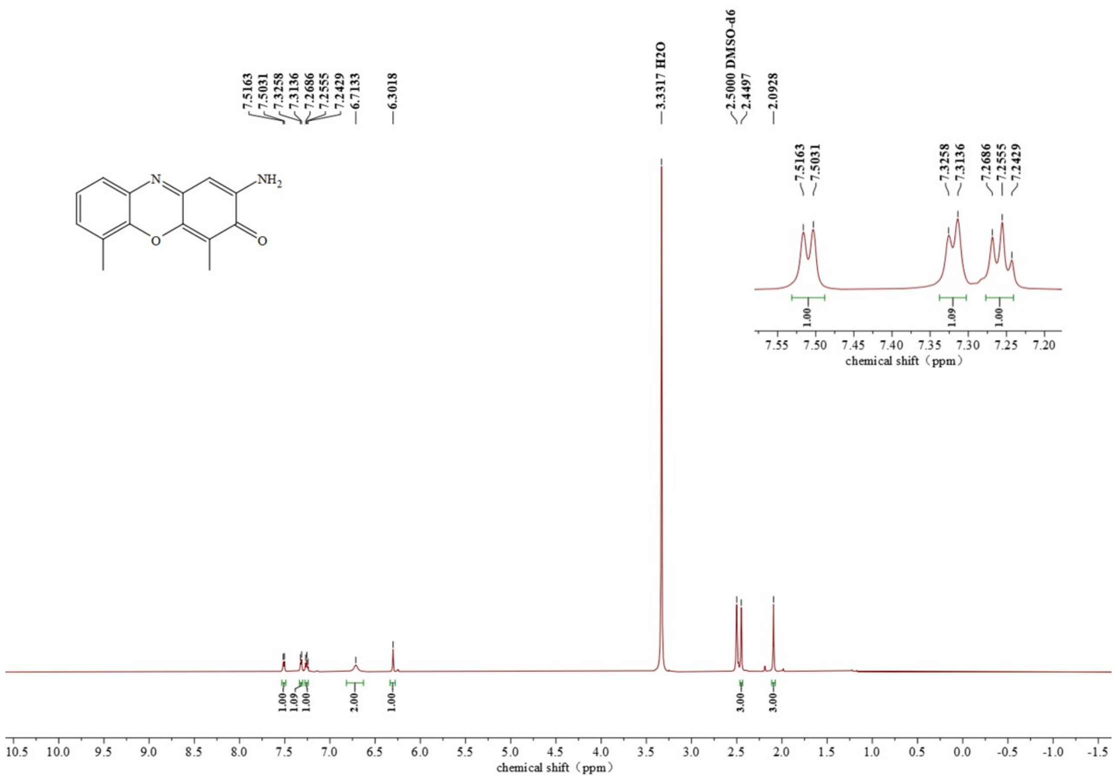
7.5163
7.5031
7.3258
7.3136
7.2686
7.2555
7.2429
6.7133
6.3018
3.3317 H2O
2.5000 DMSO-d6
2.4497
2.0928
chemical shift（ppm）

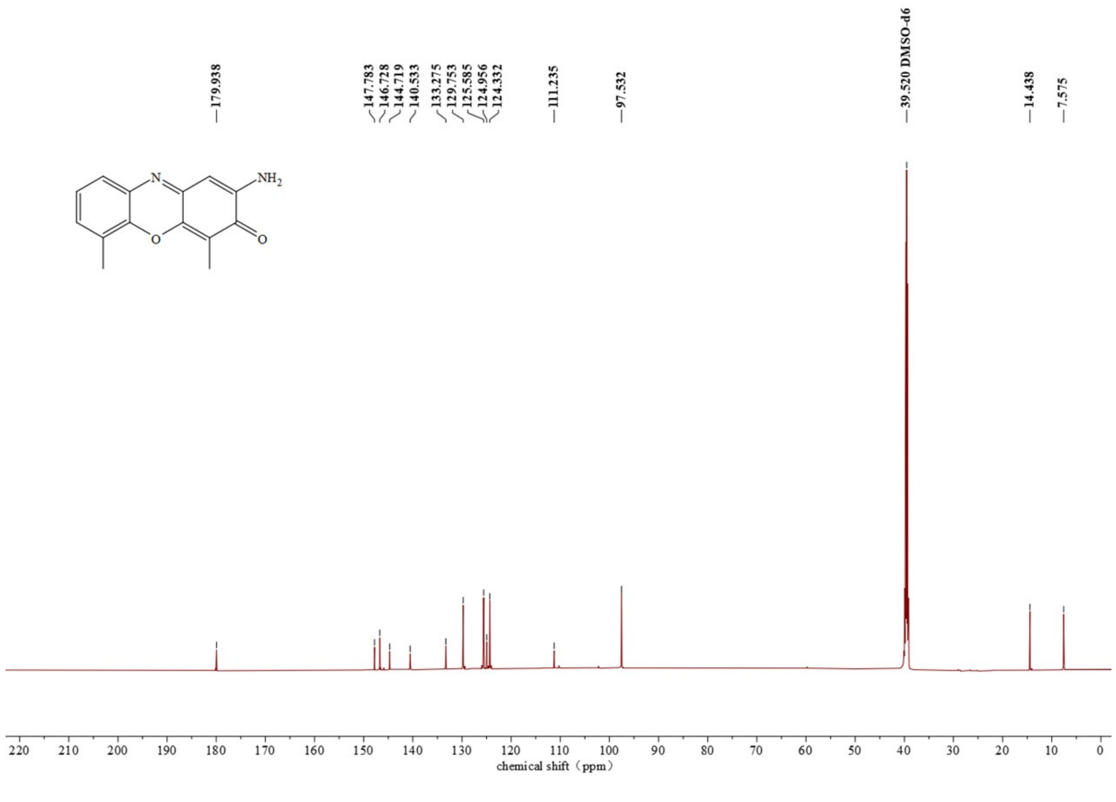
179.938
147.783
146.728
144.719
140.533
133.275
129.753
125.585
124.956
124.332
111.235
97.532
39.520 DMSO-d6
14.438
7.575
chemical shift（ppm）

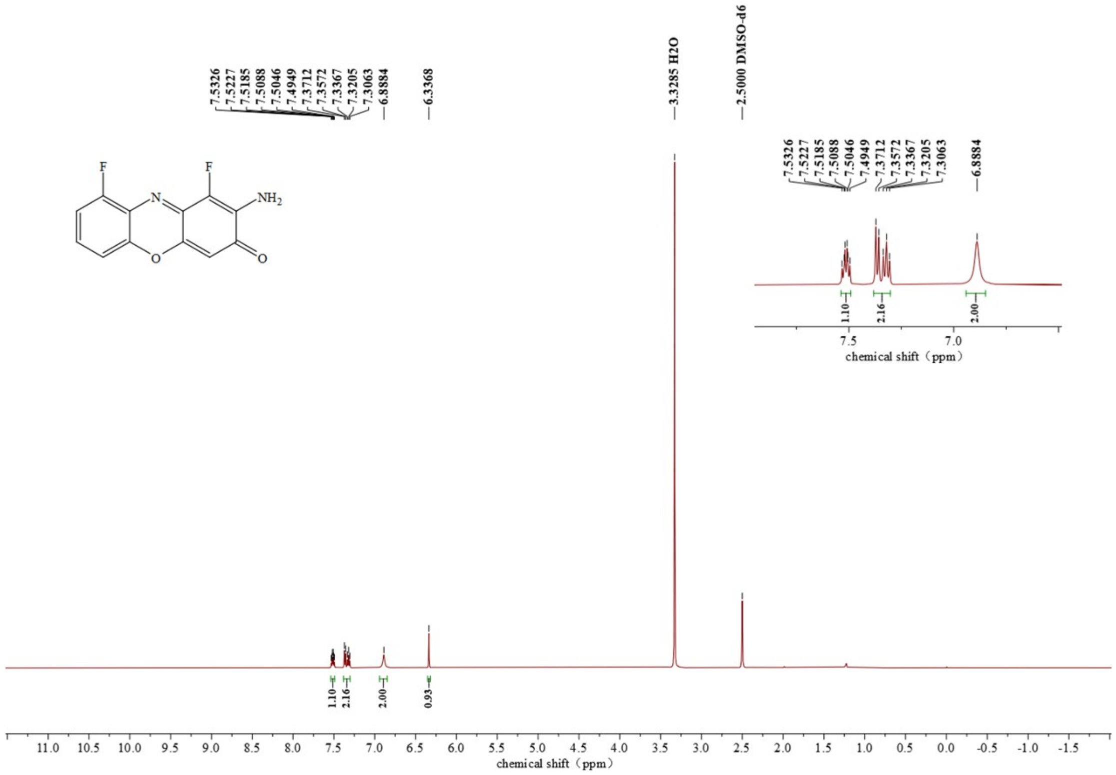
7.5326
7.5227
7.5185
7.5088
7.5046
7.4949
7.3712
7.3572
7.3367
7.3205
7.3063
6.8884
6.3368
3.3285 H2O
2.5000 DMSO-d6
F
F
N
NH2
O
O
1.10
2.16
2.00
0.93
7.5
7.0
chemical shift（ppm）
11.0 10.5 10.0 9.5 9.0 8.5 8.0 7.5 7.0 6.5 6.0 5.5 5.0 4.5 4.0 3.5 3.0 2.5 2.0 1.5 1.0 0.5 0.0 -0.5 -1.0 -1.5
chemical shift（ppm）

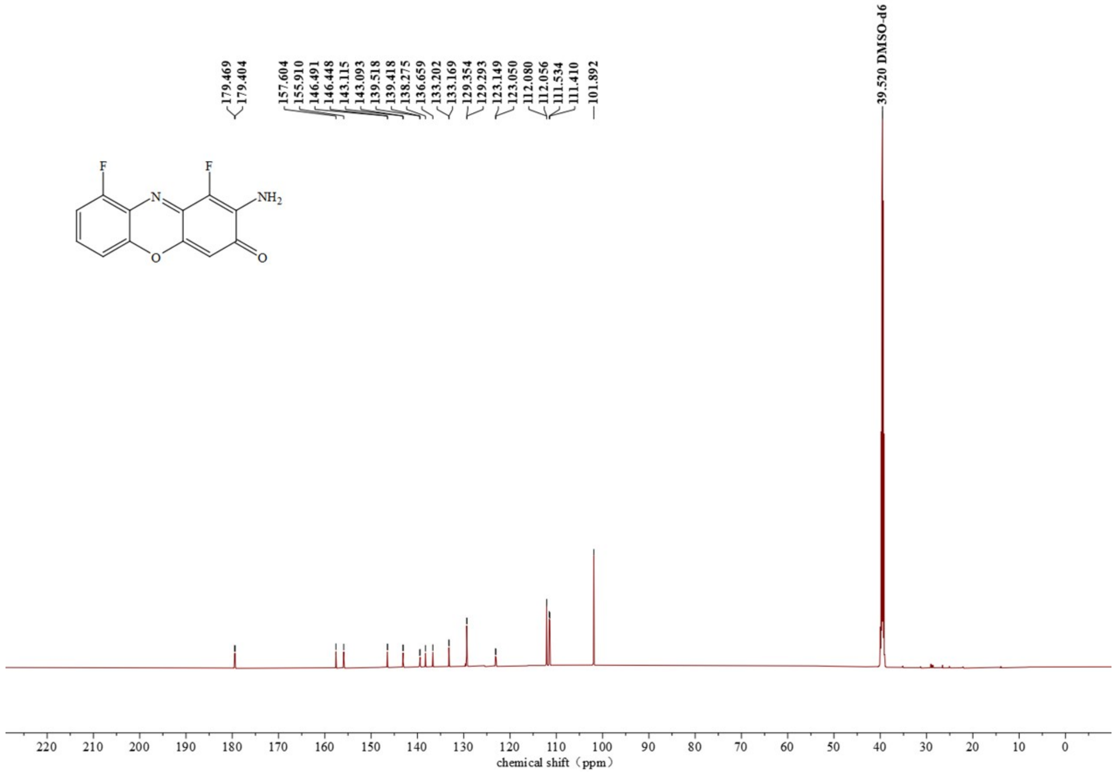
179.469
179.404
157.604
155.910
146.491
146.448
143.115
143.093
139.518
139.418
138.275
136.659
133.202
133.169
129.354
129.293
123.149
123.050
112.080
112.056
111.534
111.410
101.892
39.520 DMSO-d6
F
F
N
NH2
O
O
220 210 200 190 180 170 160 150 140 130 120 110 100 90 80 70 60 50 40 30 20 10 0
chemical shift（ppm）

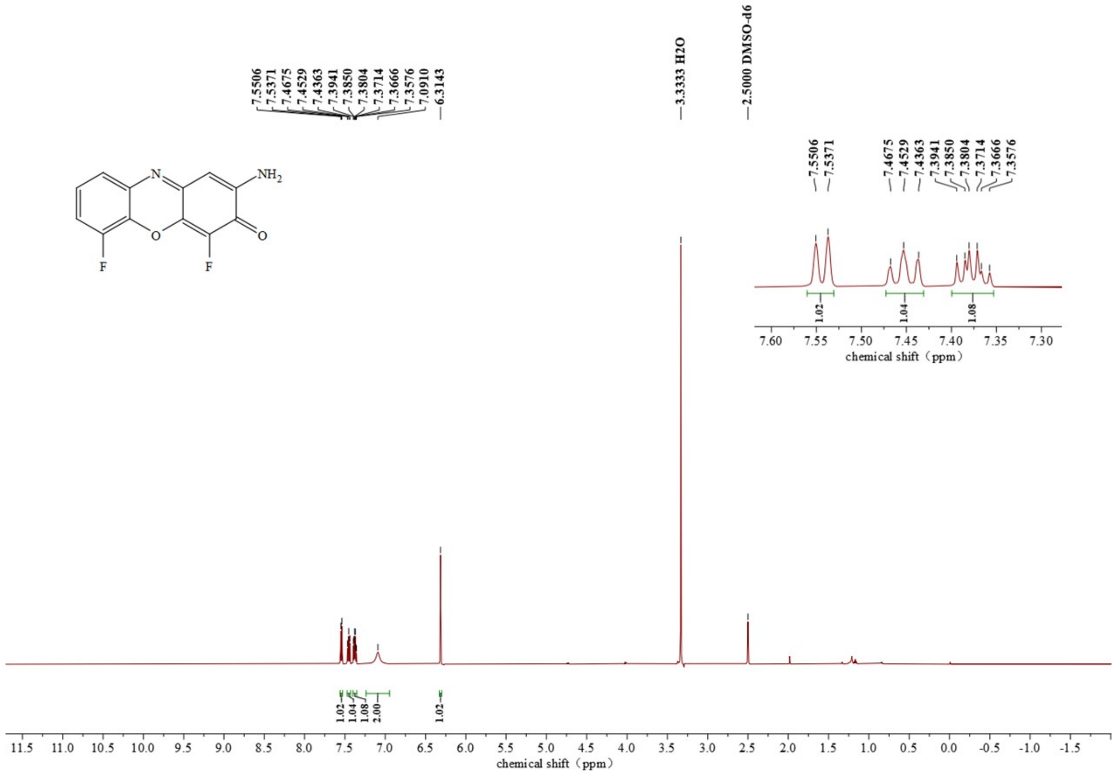
7.5506
7.5371
7.4675
7.4529
7.4363
7.3941
7.3850
7.3804
7.3714
7.3666
7.3576
7.0910
6.3143
3.3333 H2O
2.5000 DMSO-d6
NH2
O
N
F
F
1.02
1.04
1.08
2.00
1.02
chemical shift（ppm）

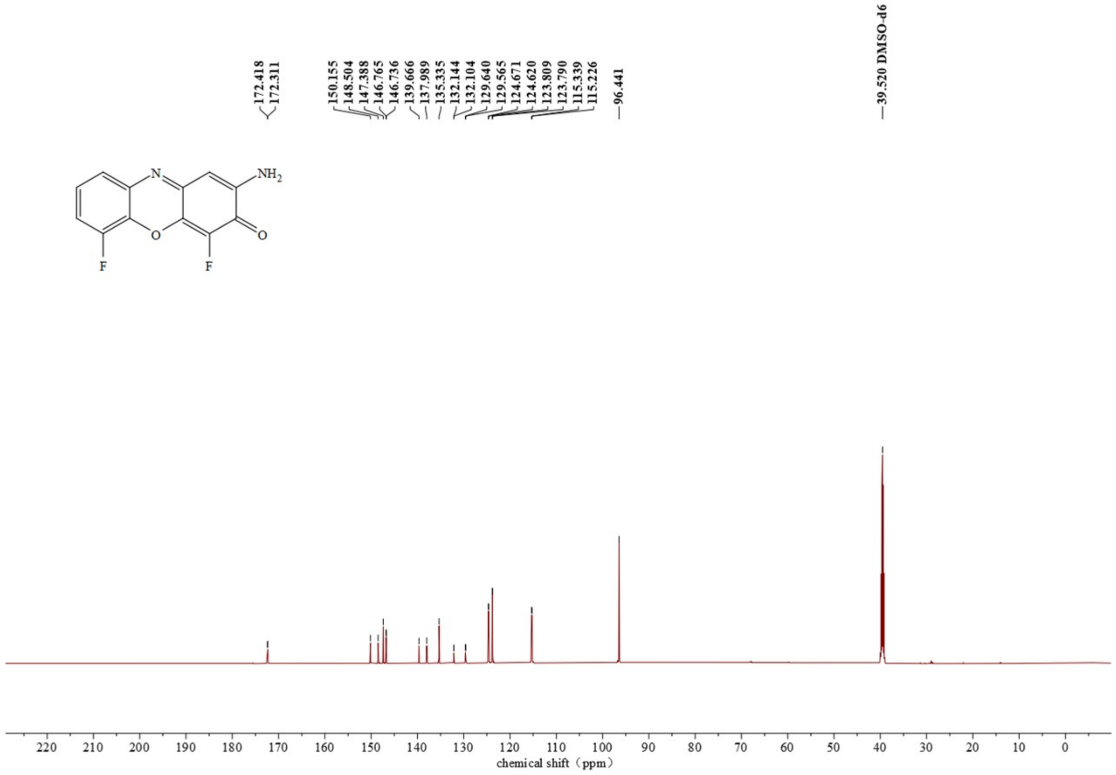
172.418
172.311
150.155
148.504
147.388
146.765
146.736
139.666
137.989
135.335
132.144
132.104
129.640
129.565
124.671
124.620
123.809
123.790
115.339
115.226
96.441
39.520 DMSO-d6
NH2
O
N
F
F
chemical shift（ppm）

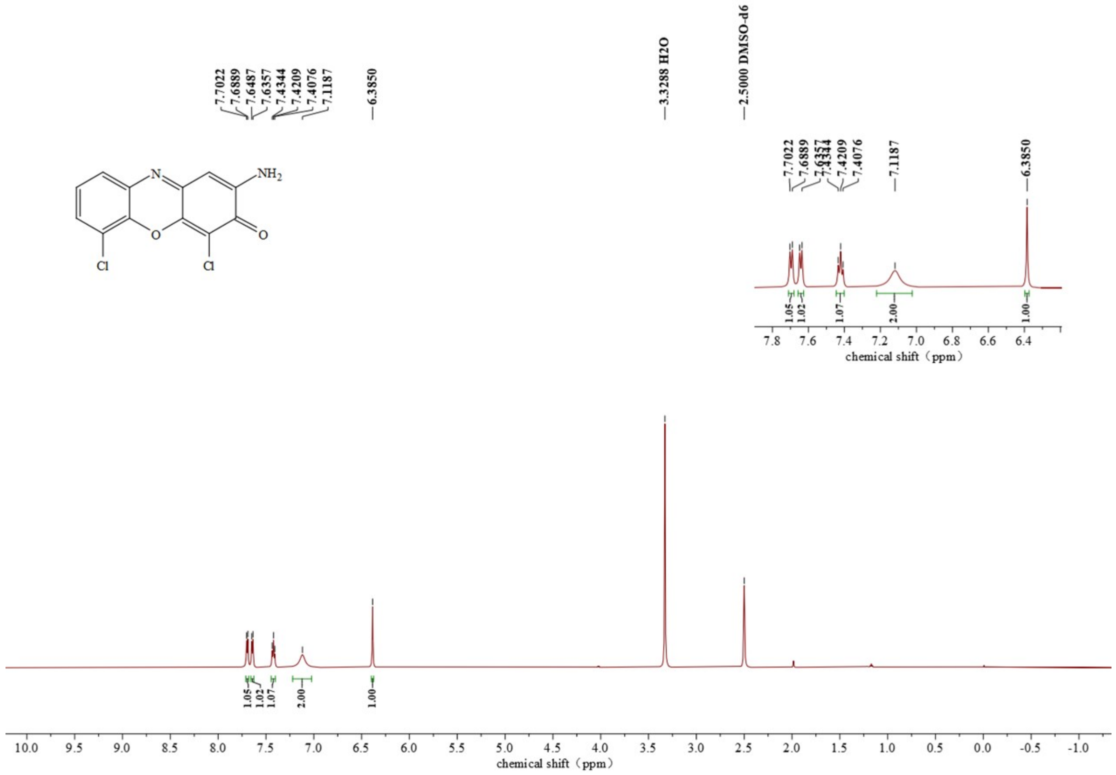
7.7022
7.6889
7.6487
7.6357
7.4344
7.4209
7.4076
7.1187
6.3850
3.3288 H2O
2.5000 DMSO-d6
NH2
O
Cl
Cl
N
1.05
1.02
1.07
2.00
1.00
chemical shift（ppm）

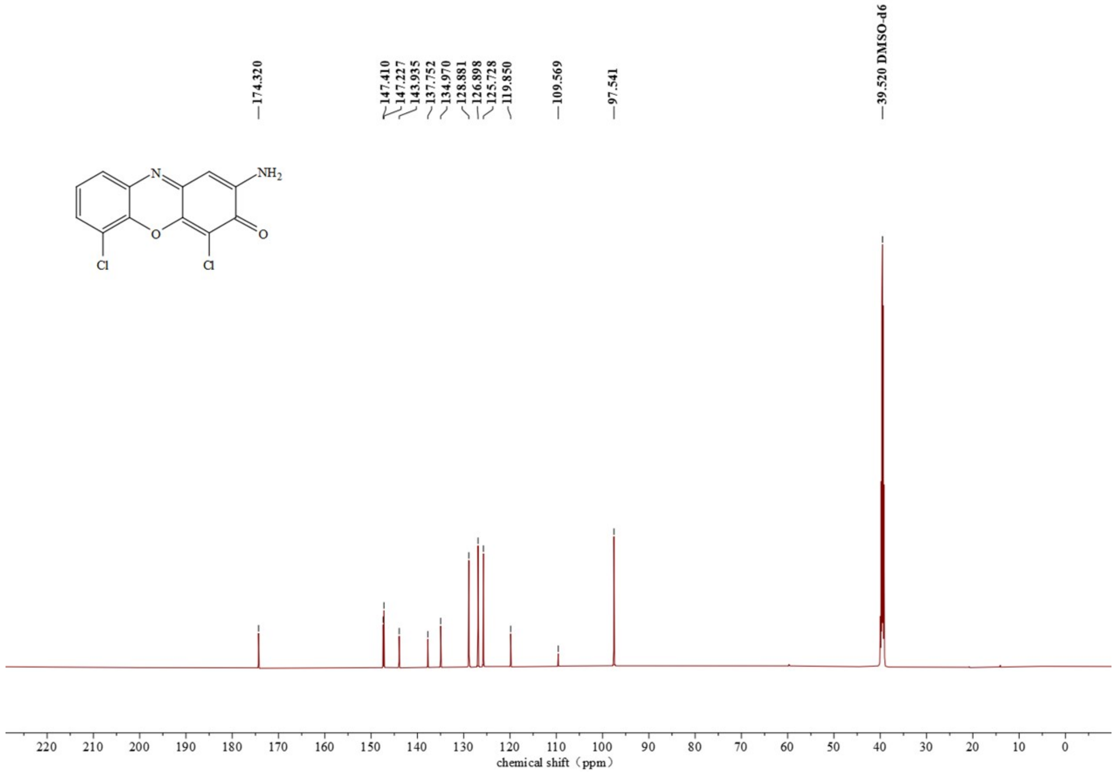
174.320
147.410
147.227
143.935
137.752
134.970
128.881
126.898
125.728
119.850
109.569
97.541
39.520 DMSO-d6
NH2
O
Cl
Cl
N
chemical shift（ppm）

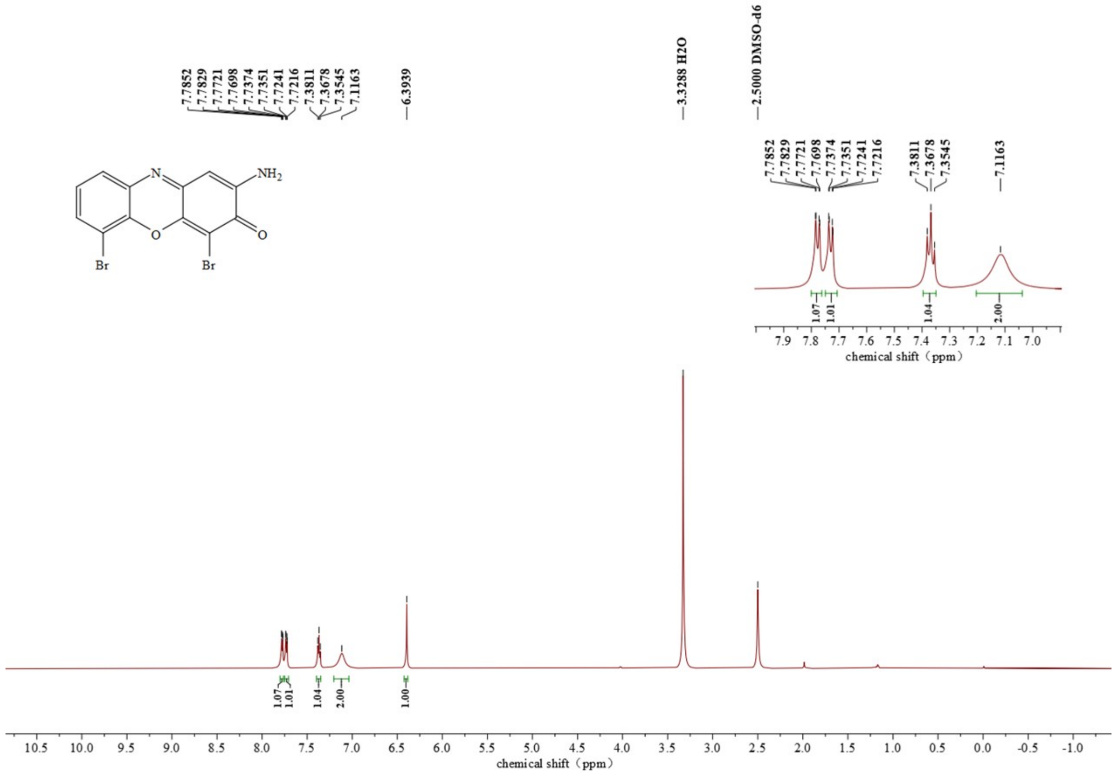
7.7852
7.7829
7.7721
7.7698
7.7374
7.7351
7.7241
7.7216
7.3811
7.3678
7.3545
7.1163
6.3939
3.3288 H2O
2.5000 DMSO-d6
NH2
O
N
Br
Br
1.07
1.01
1.04
2.00
1.00
chemical shift（ppm）

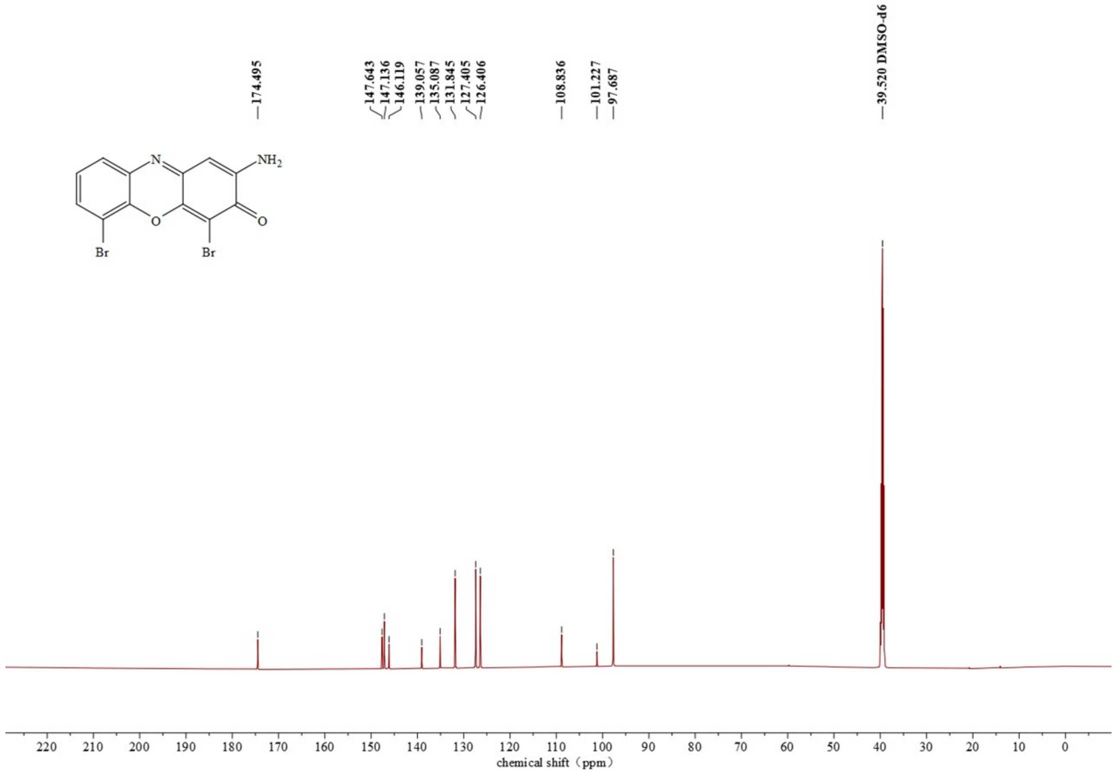
174.495
147.643
147.136
146.119
139.057
135.087
131.845
127.405
126.406
108.836
101.227
97.687
39.520 DMSO-d6
NH2
O
N
Br
Br
chemical shift（ppm）

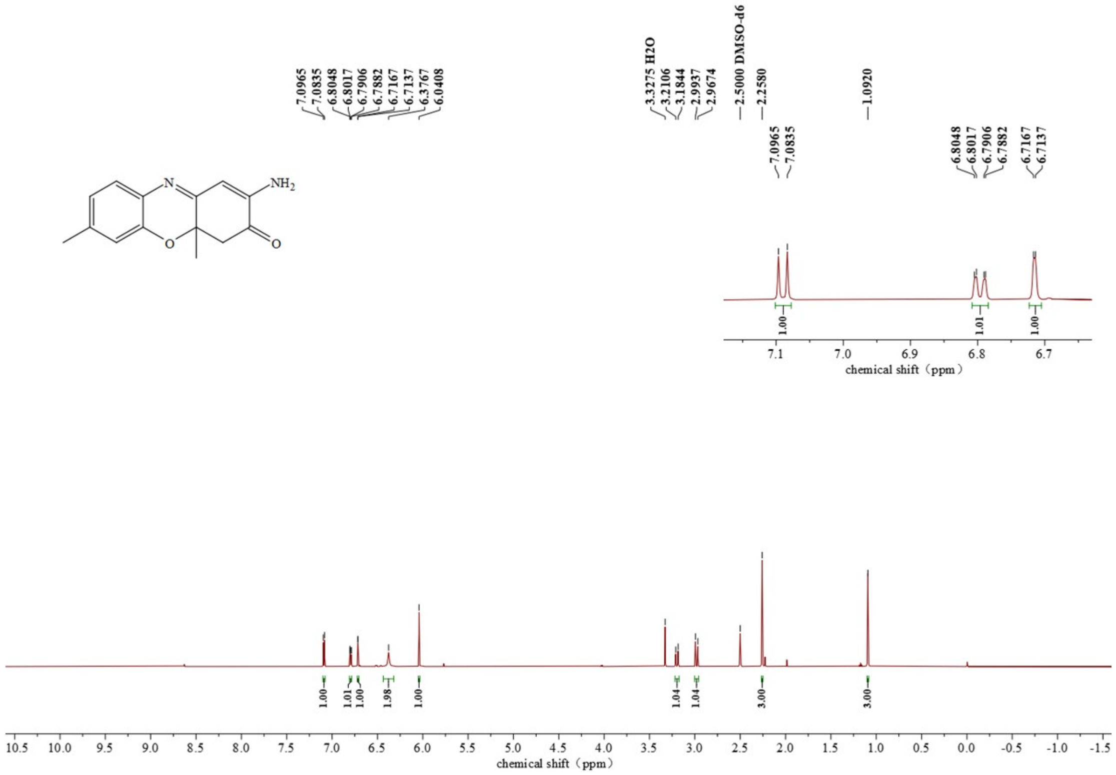
7.0965
7.0835
6.8048
6.8017
6.7906
6.7882
6.7167
6.7137
6.3767
6.0408
3.3275 H2O
3.2106
3.1844
2.9937
2.9674
2.5000 DMSO-d6
2.2580
1.0920
NH2
O
N
chemical shift（ppm）

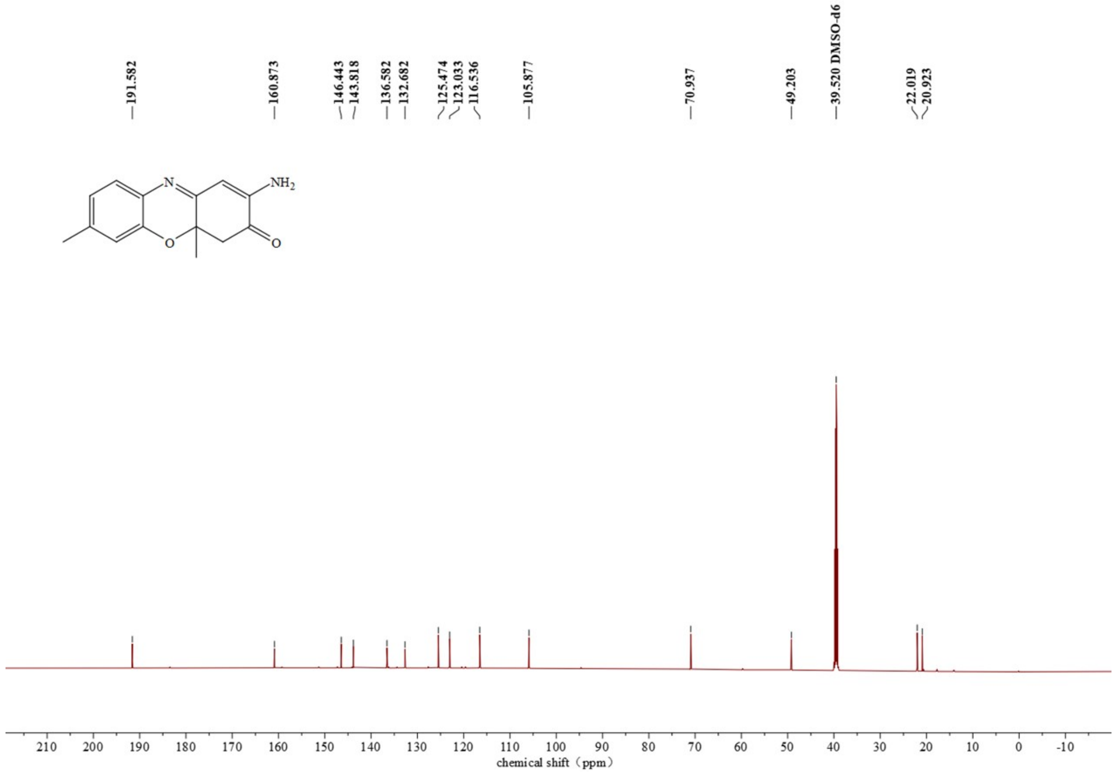
191.582
160.873
146.443
143.818
136.582
132.682
125.474
123.033
116.536
105.877
70.937
49.203
39.520 DMSO-d6
22.019
20.923
NH2
O
N
chemical shift（ppm）

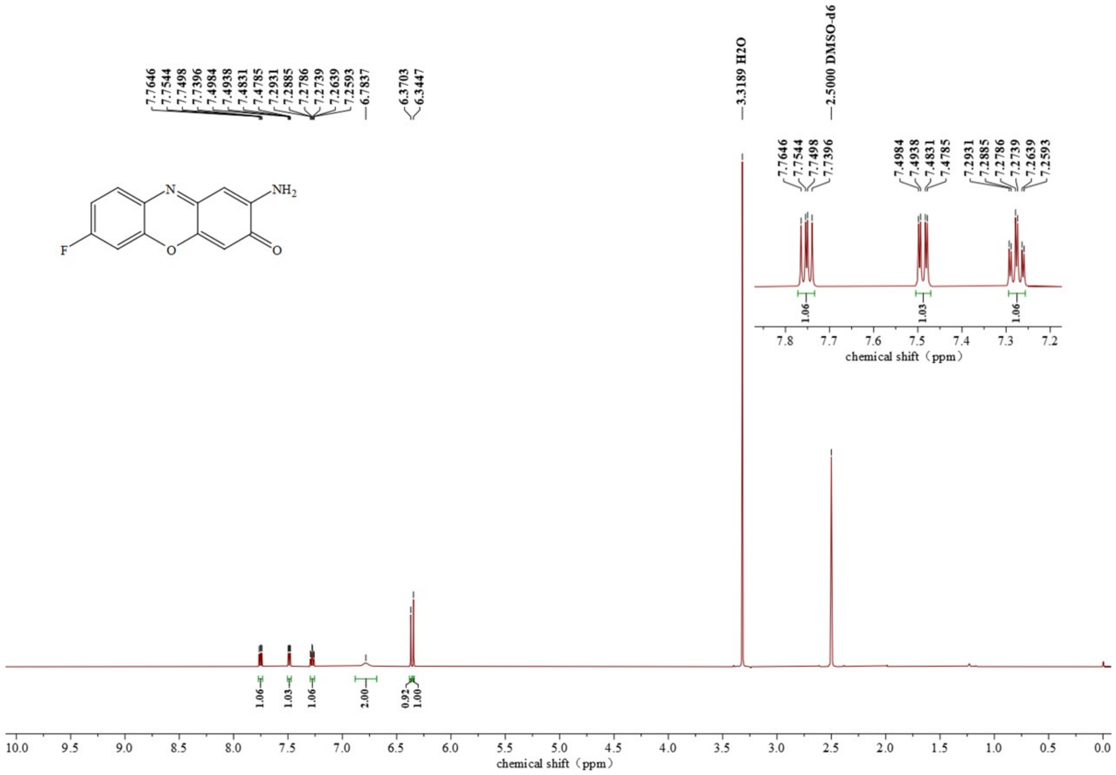
7.7646
7.7544
7.7498
7.7396
7.4984
7.4938
7.4831
7.4785
7.2931
7.2885
7.2786
7.2739
7.2639
7.2593
6.7837
6.3703
6.3447
3.3189 H2O
2.5000 DMSO-d6
F
N
O
NH2
O
1.06
1.03
1.06
2.00
0.92
1.00
chemical shift（ppm）

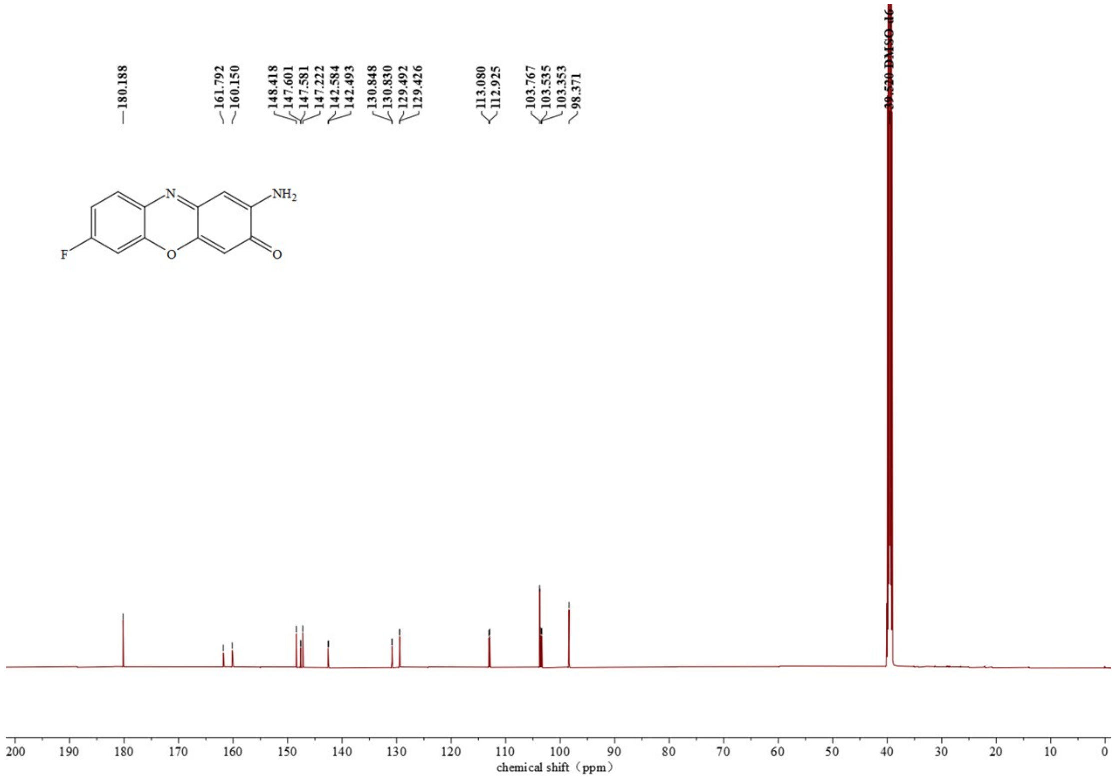
180.188
161.792
160.150
148.418
147.601
147.581
147.222
142.584
142.493
130.848
130.830
129.492
129.426
113.080
112.925
103.767
103.535
103.353
98.371
F
N
O
NH2
O
chemical shift（ppm）

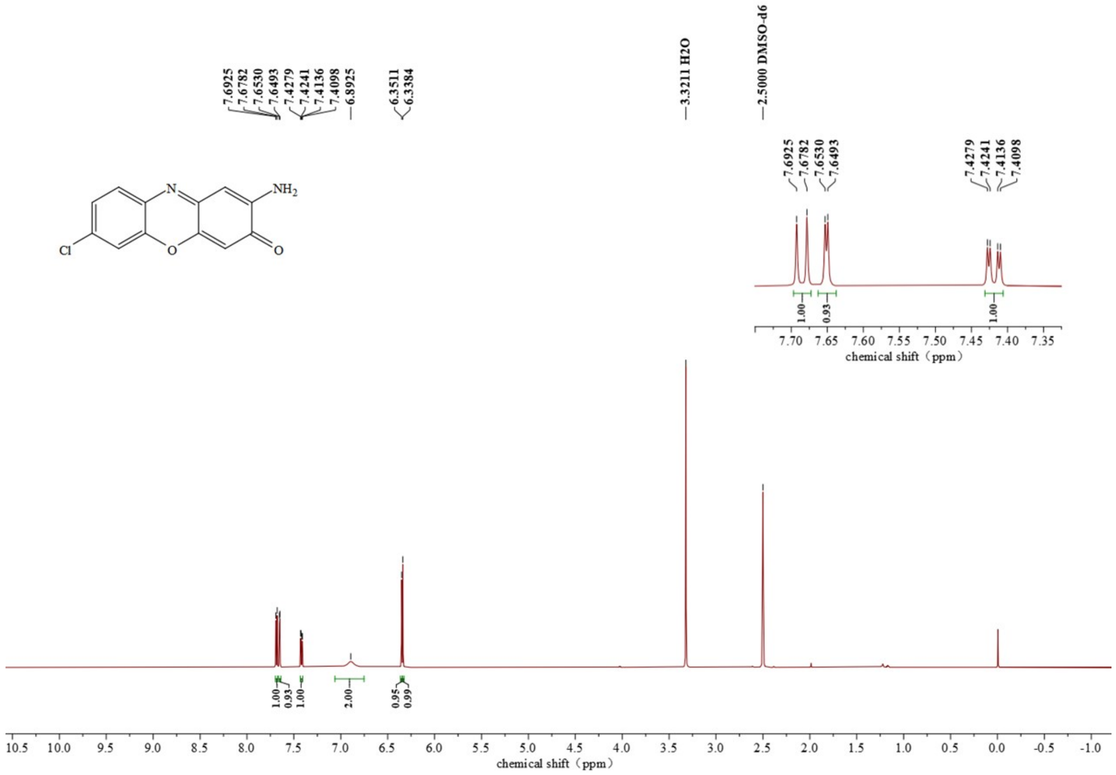
7.6925
7.6782
7.6530
7.6493
7.4279
7.4241
7.4136
7.4098
6.8925
6.3511
6.3384
3.3211 H2O
2.5000 DMSO-d6
NH2
Cl
N
O
1.00
0.93
1.00
2.00
0.95
0.99
chemical shift（ppm）

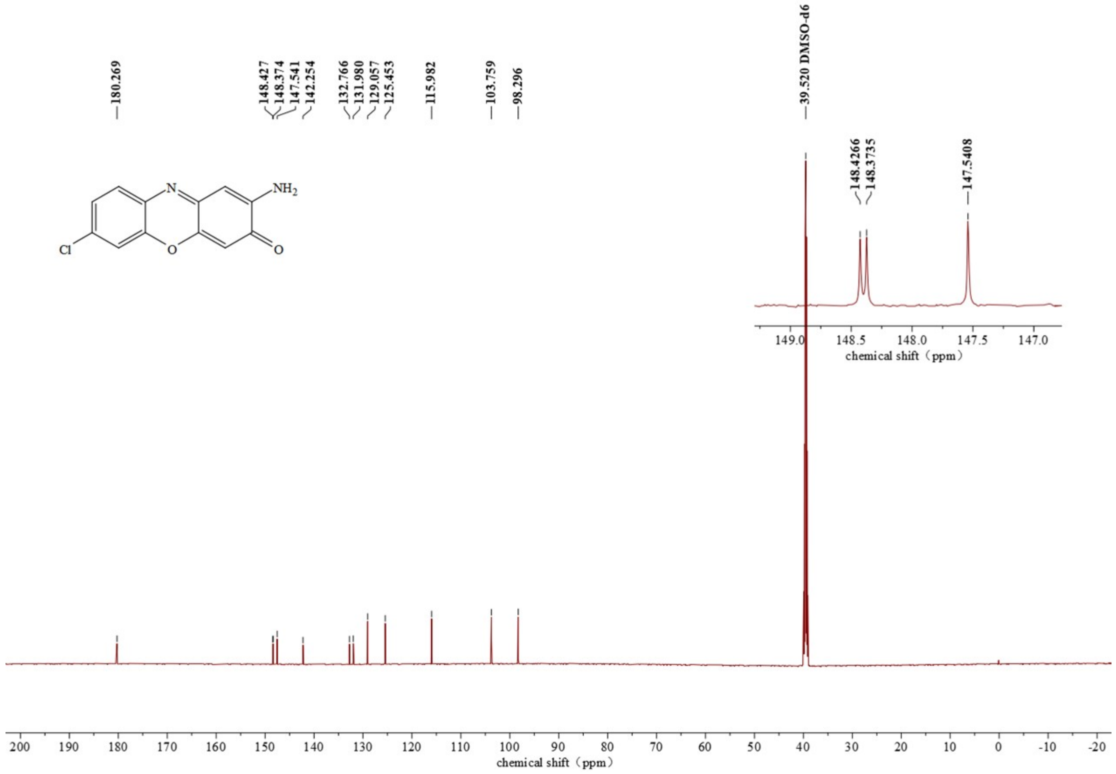
180.269
148.427
148.374
147.541
142.254
132.766
131.980
129.057
125.453
115.982
103.759
98.296
39.520 DMSO-d6
148.4266
148.3735
147.5408
NH2
Cl
N
O
chemical shift（ppm）

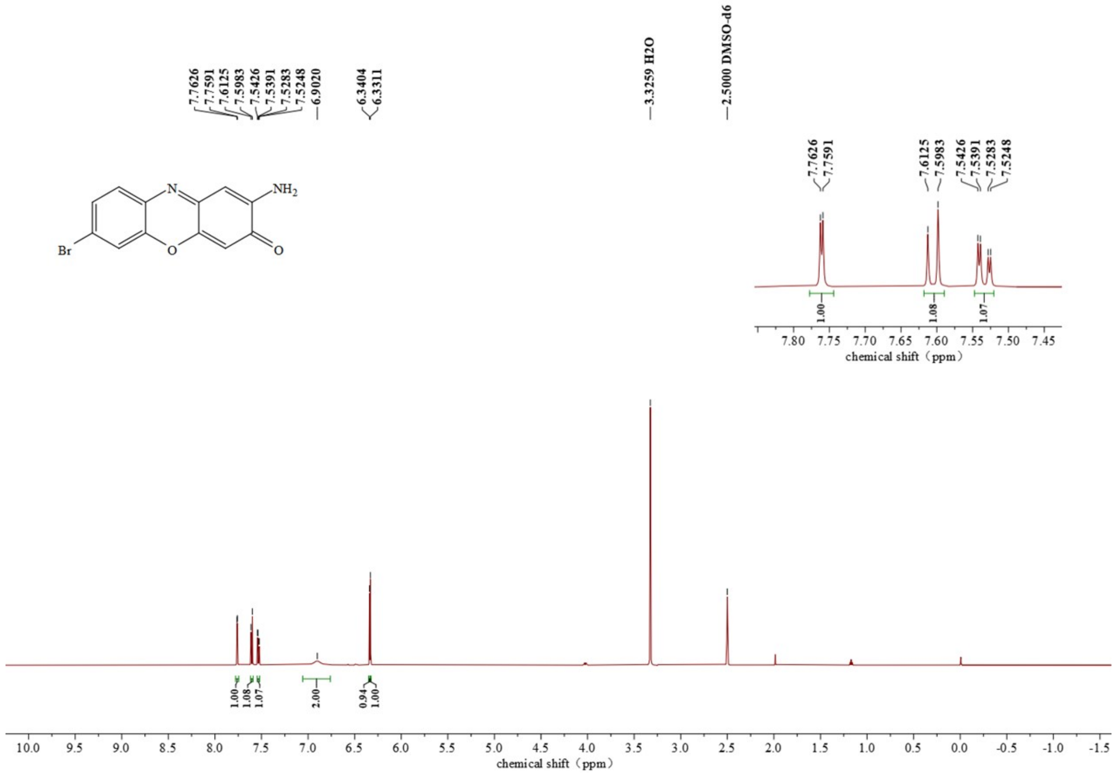
7.7626
7.7591
7.6125
7.5983
7.5426
7.5391
7.5283
7.5248
6.9020
6.3404
6.3311
3.3259 H2O
2.5000 DMSO-d6
NH_2
O
N
Br
1.00
1.08
1.07
2.00
0.94
1.00
chemical shift（ppm）

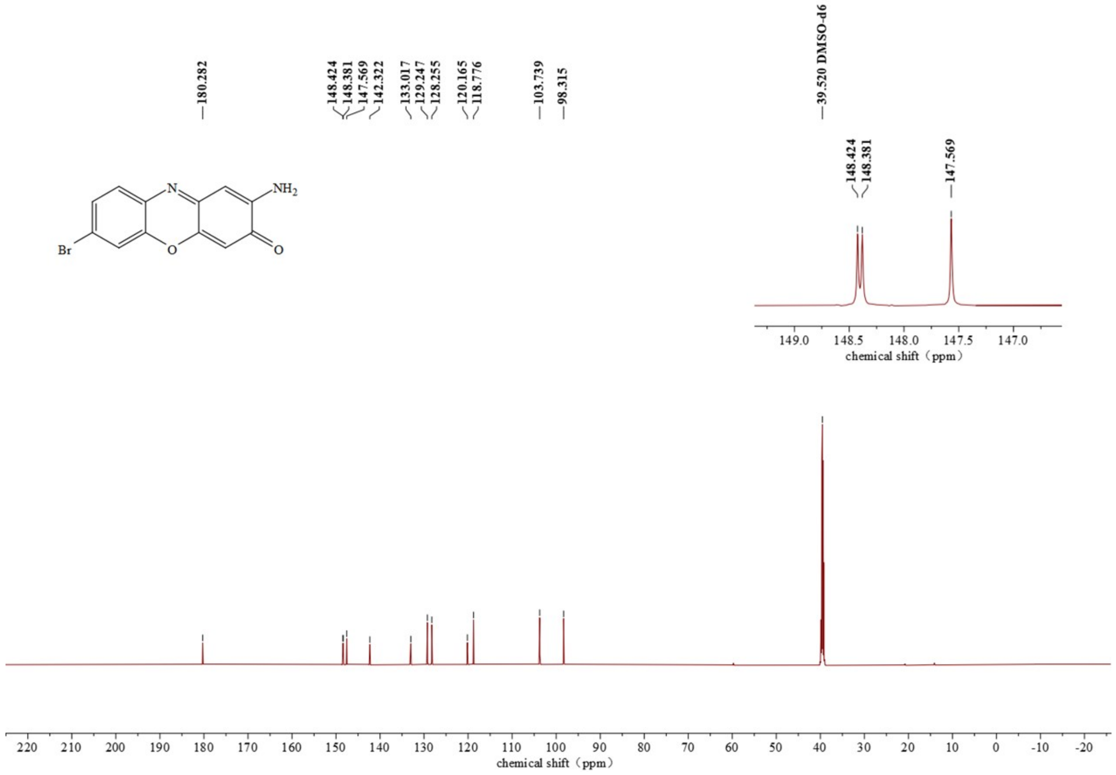
180.282
148.424
148.381
147.569
142.322
133.017
129.247
128.255
120.165
118.776
103.739
98.315
39.520 DMSO-d6
NH_2
O
N
Br
chemical shift（ppm）

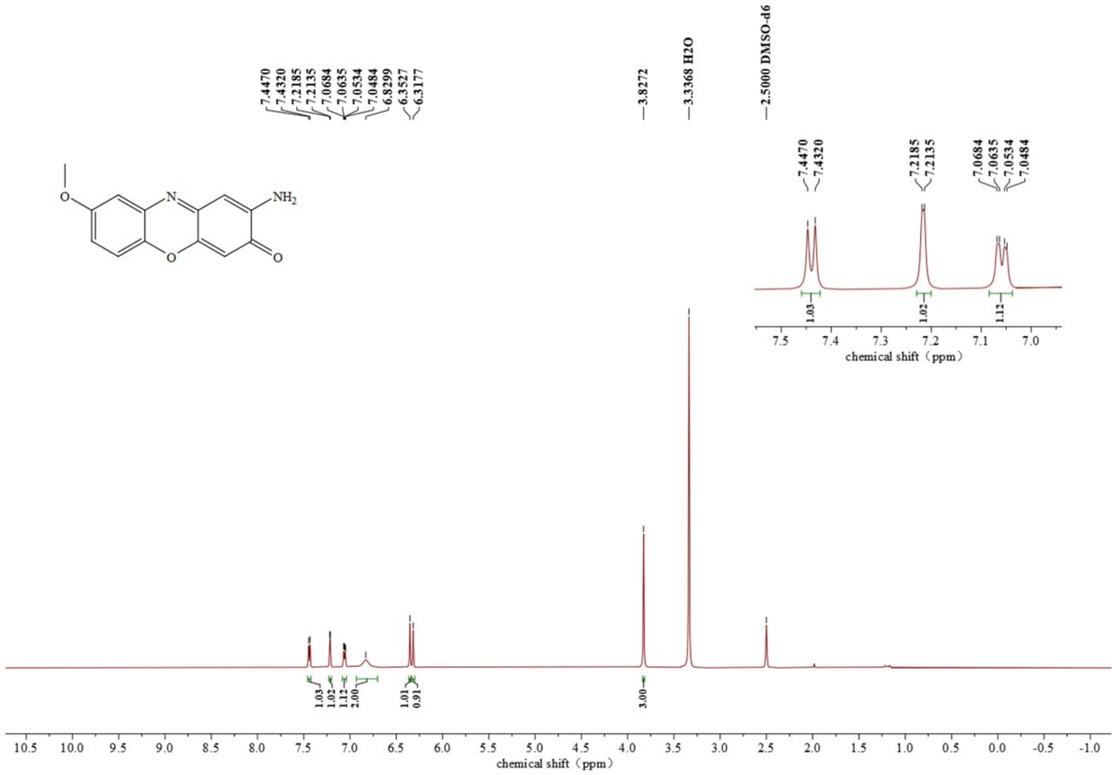
7.4470
7.4320
7.2185
7.2135
7.0684
7.0635
7.0534
7.0484
6.8299
6.3527
6.3177
3.8272
3.3368 H2O
2.5000 DMSO-d6
NH2
O
N
1.03
1.02
1.12
2.00
1.01
0.91
3.00
chemical shift（ppm）

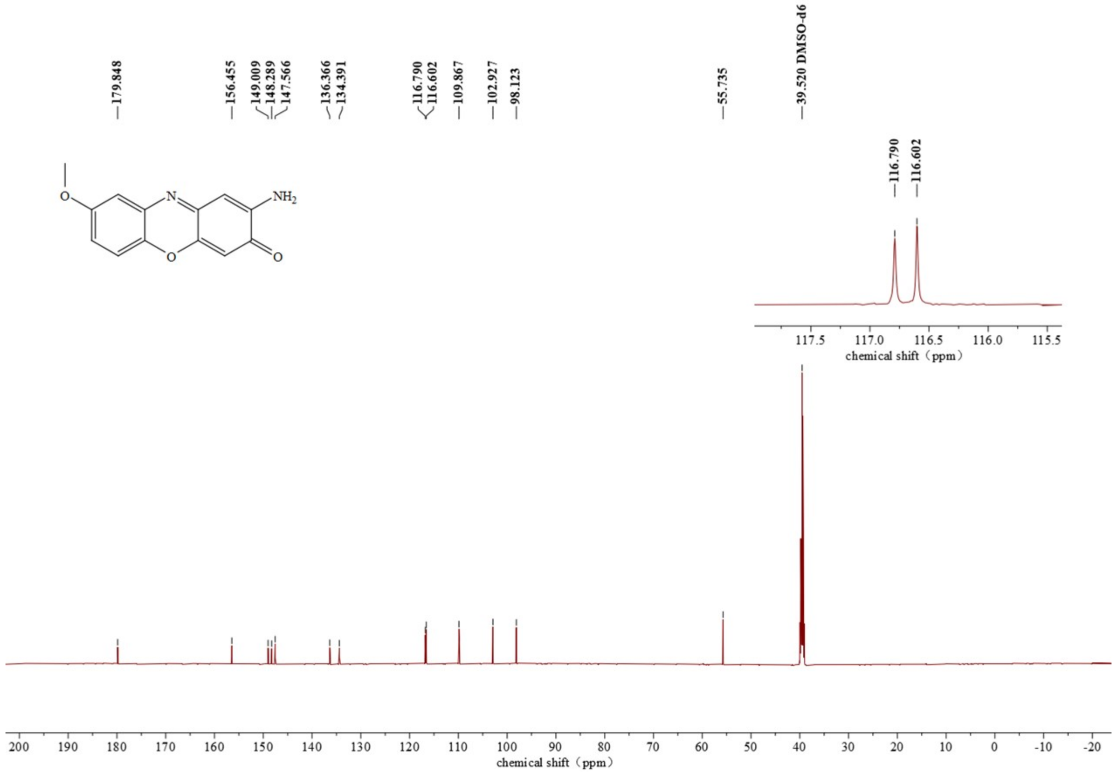
179.848
156.455
149.009
148.289
147.566
136.366
134.391
116.790
116.602
109.867
102.927
98.123
55.735
39.520 DMSO-d6
NH2
O
N
chemical shift（ppm）

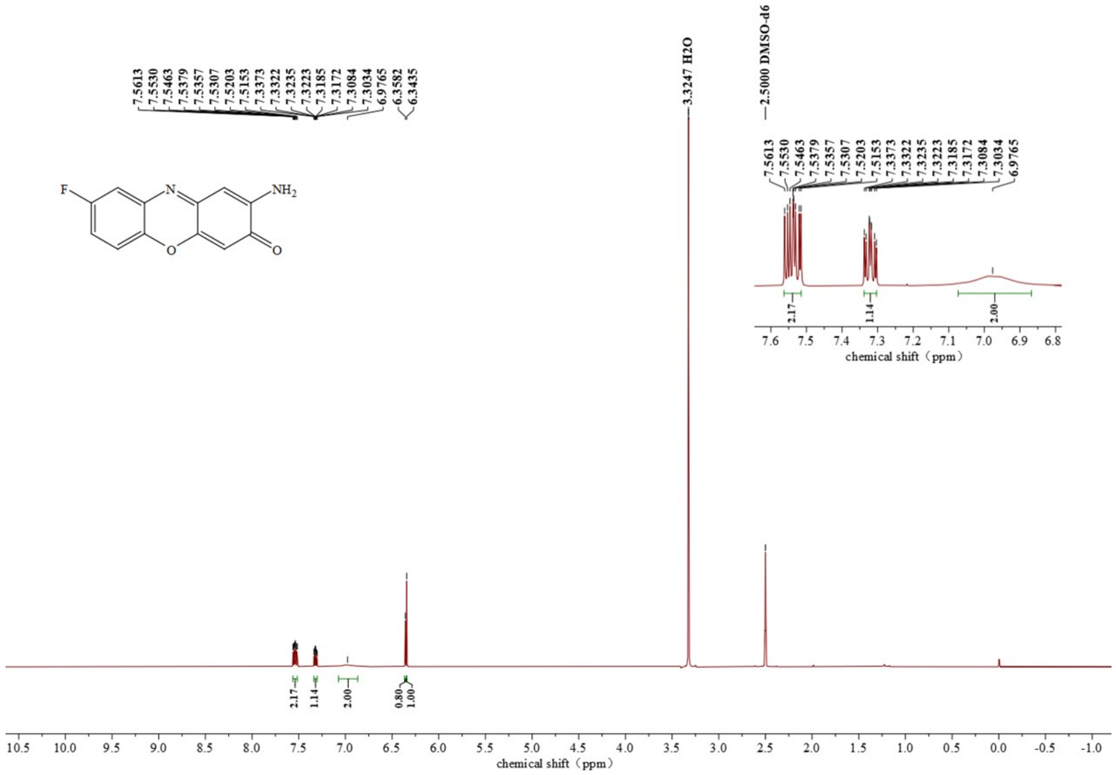

7.5613
7.5530
7.5463
7.5379
7.5357
7.5307
7.5203
7.5153
7.3373
7.3322
7.3235
7.3223
7.3185
7.3172
7.3084
7.3034
6.9765
6.3582
6.3435
3.3247 H2O
2.5000 DMSO-d6
F
NH2
O
2.17
1.14
2.00
0.80
1.00
chemical shift（ppm）

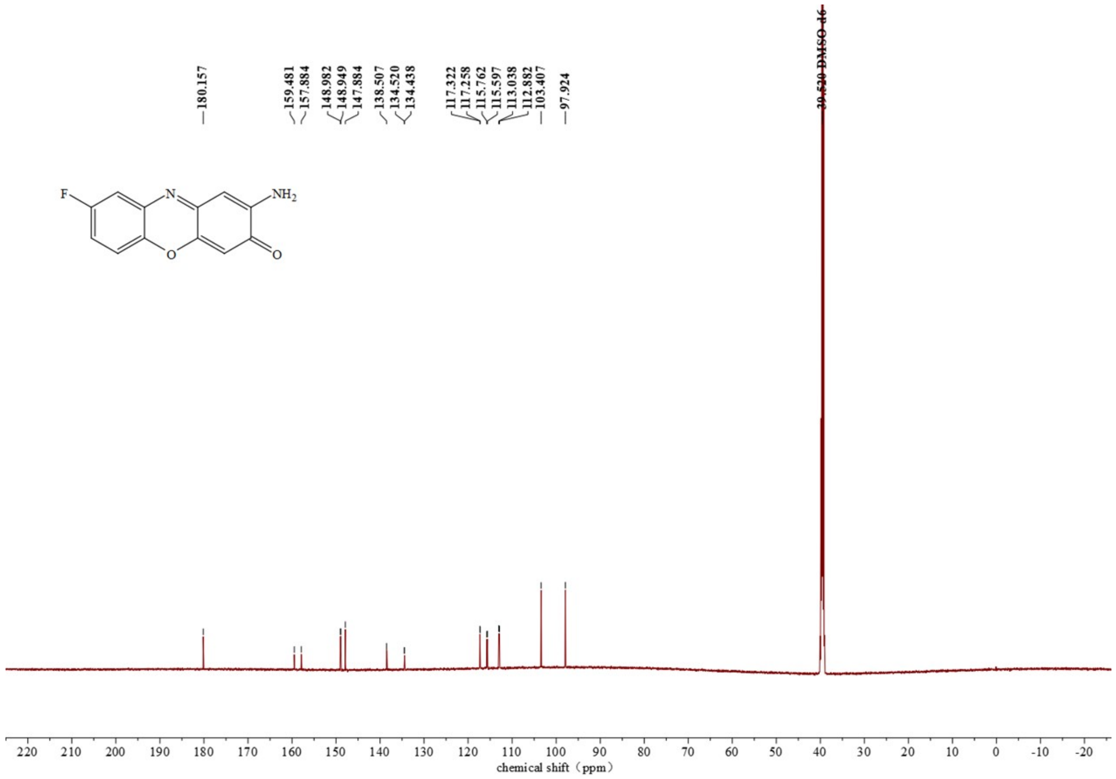

180.157
159.481
157.884
148.982
148.949
147.884
138.507
134.520
134.438
117.322
117.258
115.762
115.597
113.038
112.882
103.407
97.924
F
NH2
O
chemical shift（ppm）

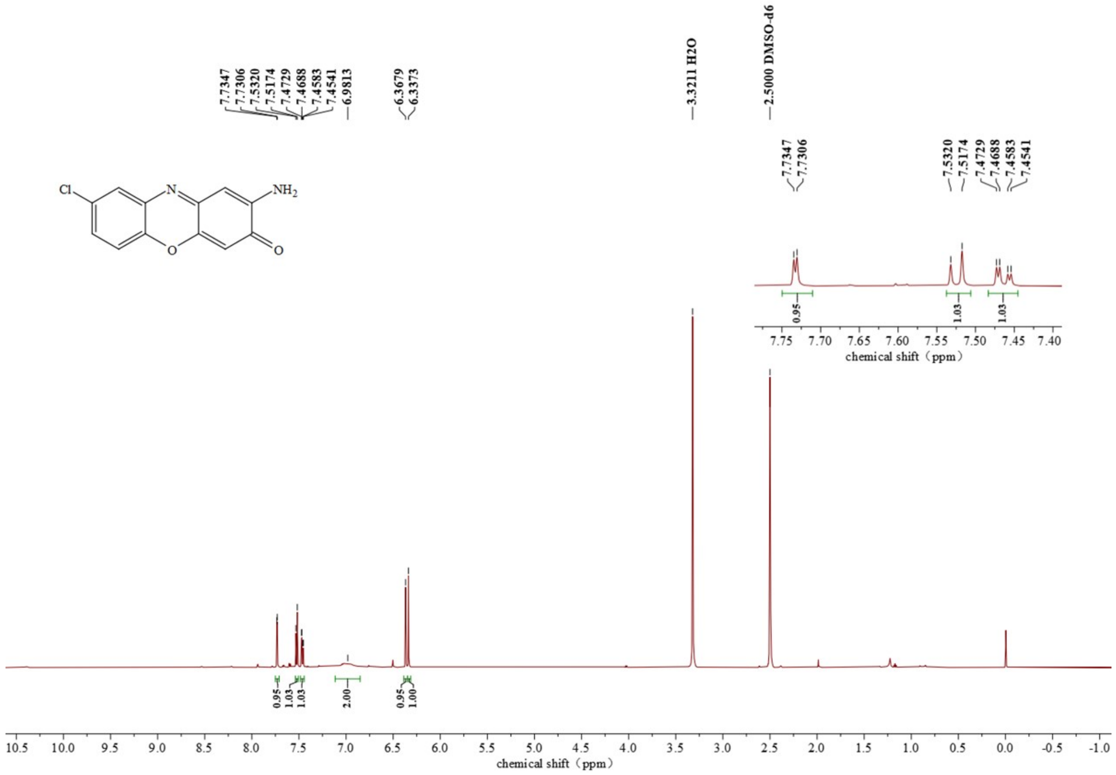
7.7347
7.7306
7.5320
7.5174
7.4729
7.4688
7.4583
7.4541
6.9813
6.3679
6.3373
3.3211 H2O
2.5000 DMSO-d6
Cl
N
NH2
O
O
0.95
1.03
1.03
2.00
0.95
1.00
chemical shift（ppm）

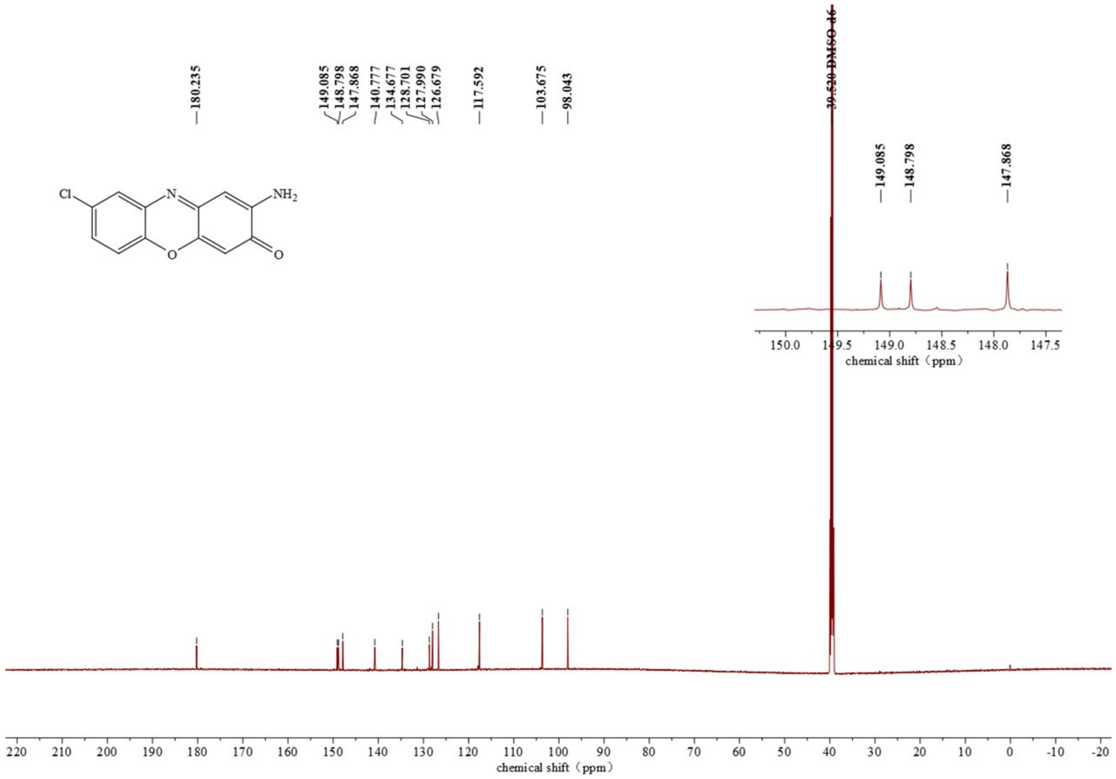
180.235
149.085
148.798
147.868
140.777
134.677
128.701
127.990
126.679
117.592
103.675
98.043
39.520 DMSO-d6
Cl
N
NH2
O
O
chemical shift（ppm）

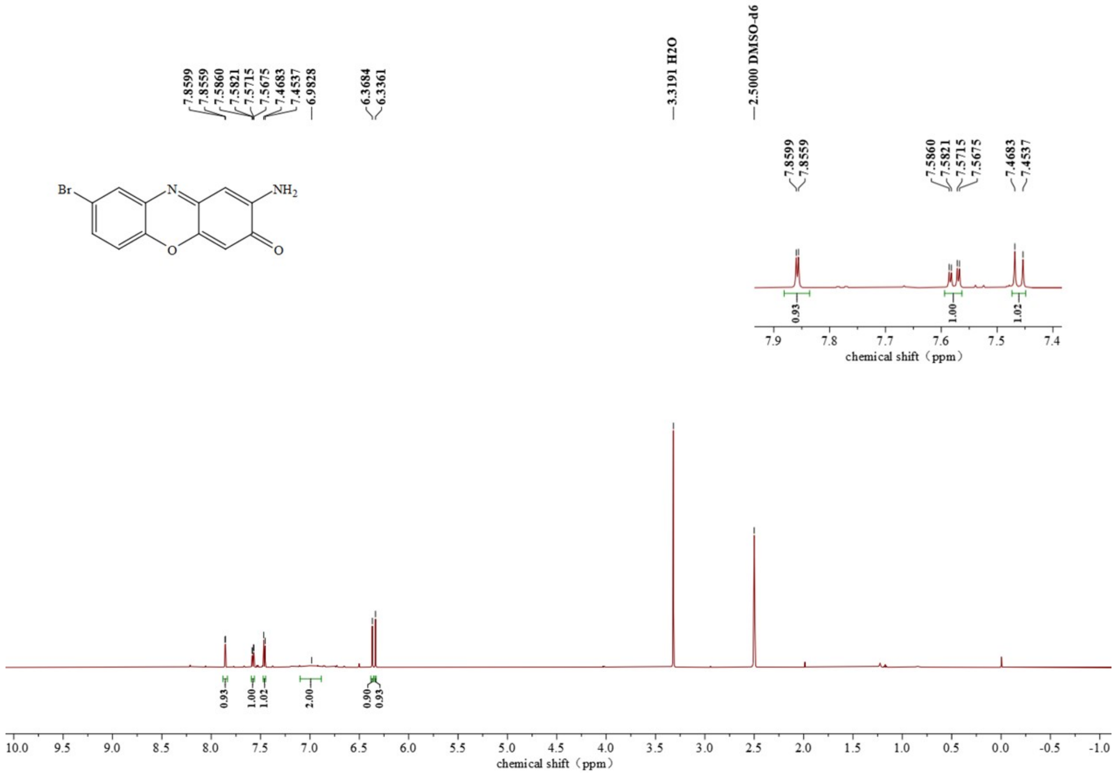

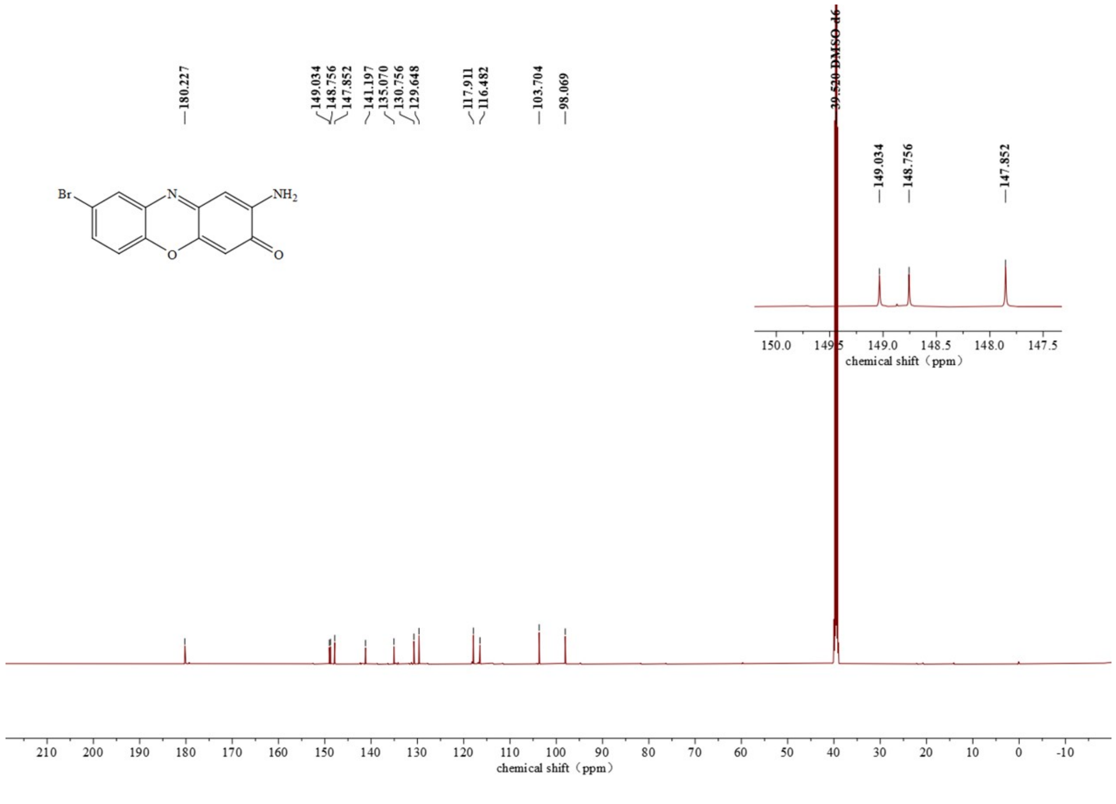

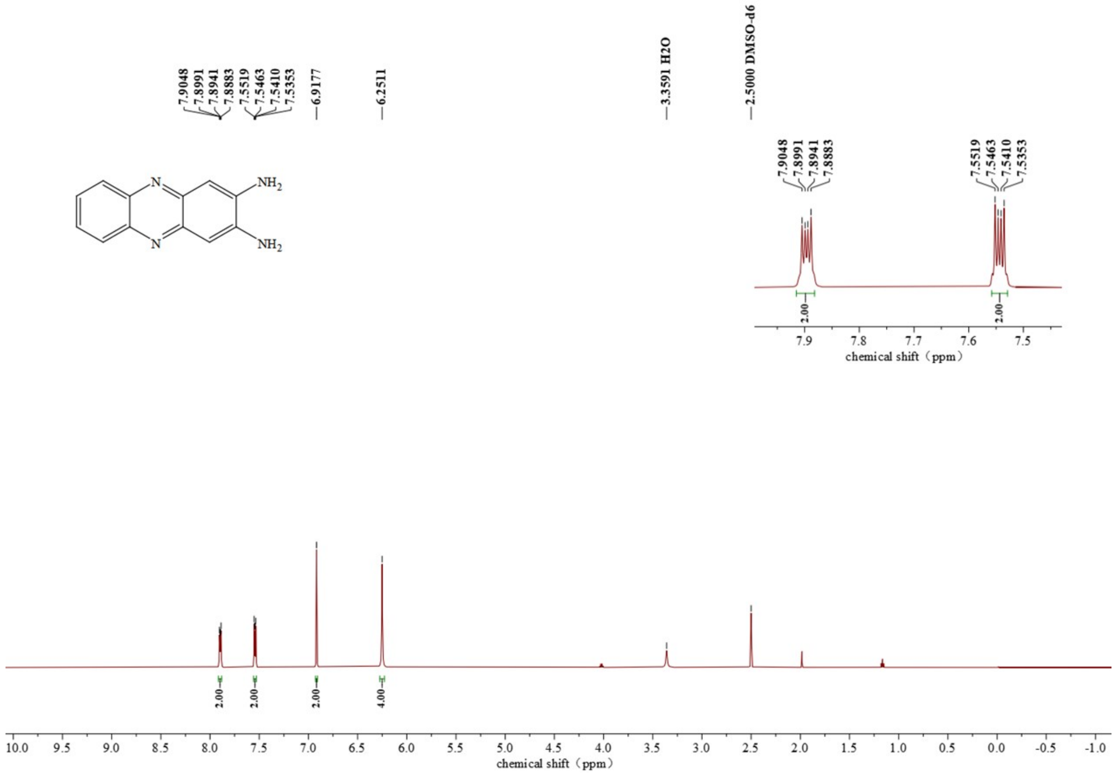
7.9048
7.8991
7.8941
7.8883
7.5519
7.5463
7.5410
7.5353
6.9177
6.2511
3.3591 H2O
2.5000 DMSO-d6
NH2
NH2
N
N
2.00
2.00
2.00
4.00
chemical shift（ppm）

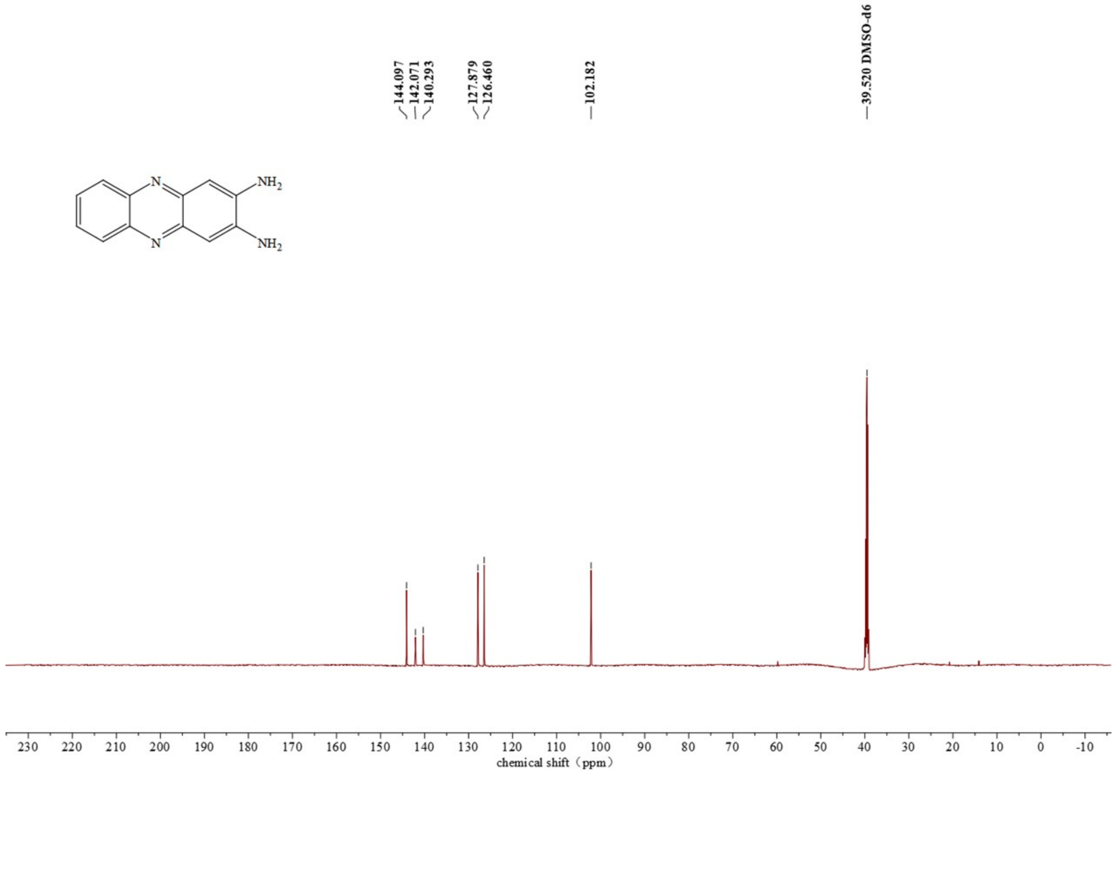
144.097
142.071
140.293
127.879
126.460
102.182
39.520 DMSO-d6
NH2
NH2
N
N
chemical shift（ppm）

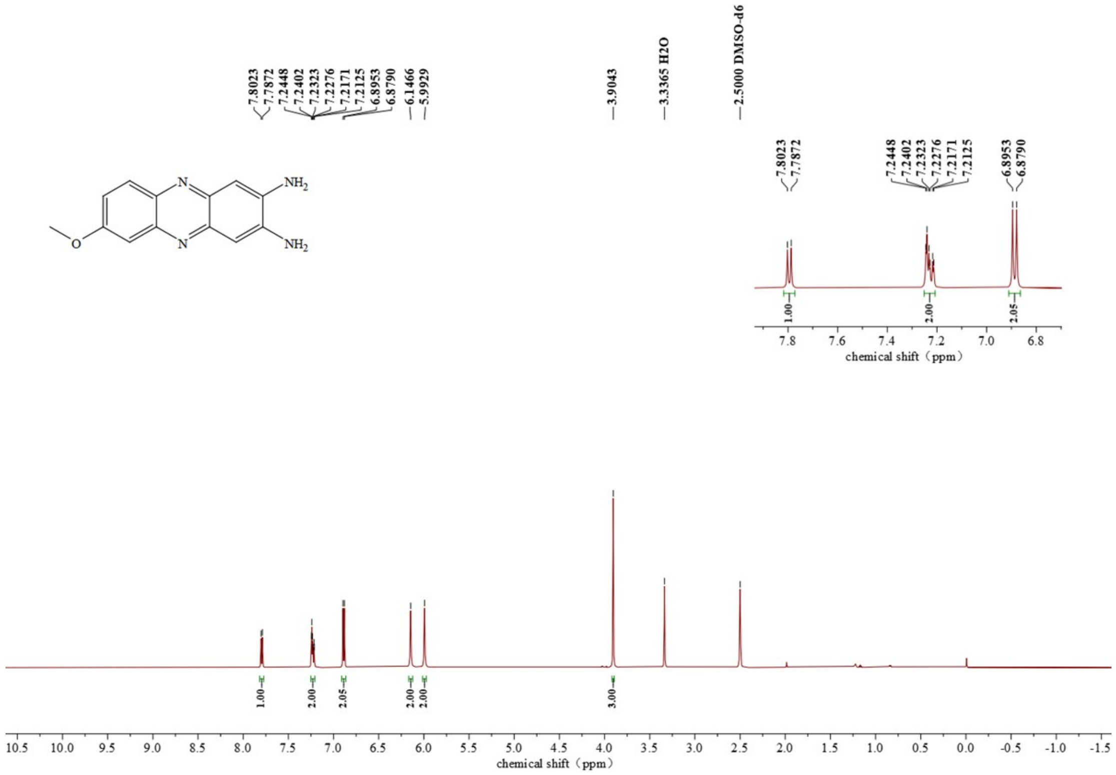
7.8023
7.7872
7.2448
7.2402
7.2323
7.2276
7.2171
7.2125
6.8953
6.8790
6.1466
5.9929
3.9043
3.3365 H2O
2.5000 DMSO-d6
1.00
2.00
2.05
2.00
2.00
3.00
chemical shift（ppm）

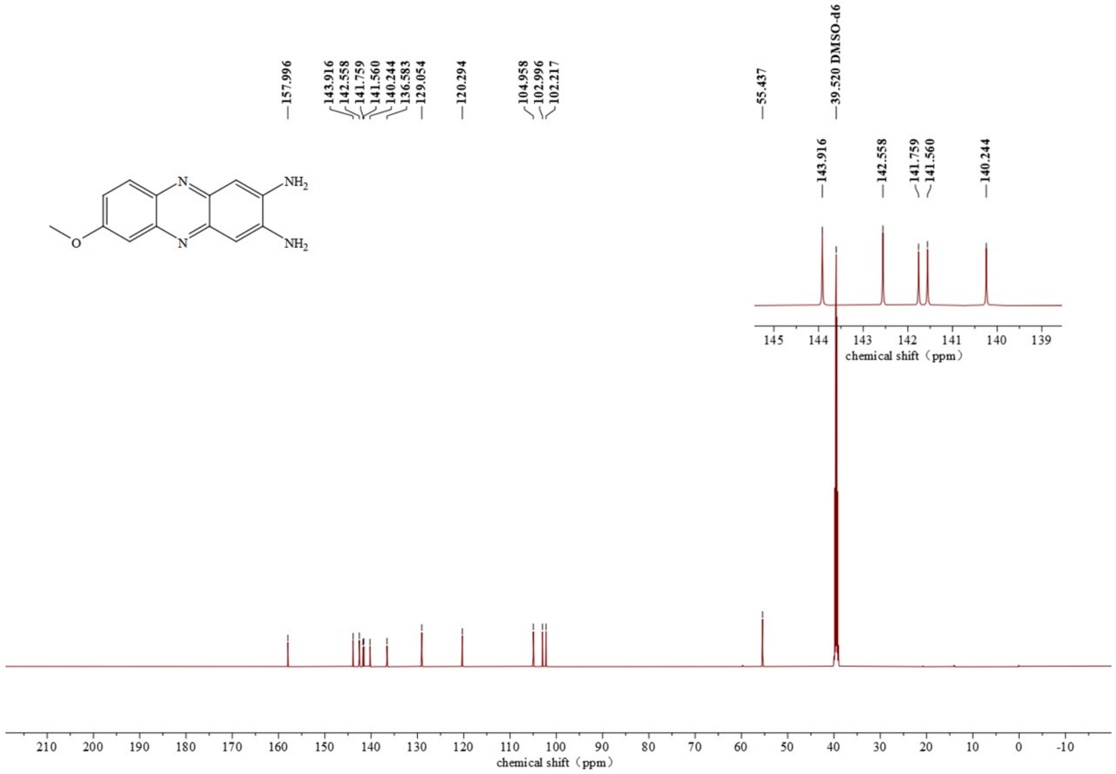
157.996
143.916
142.558
141.759
141.560
140.244
136.583
129.054
120.294
104.958
102.996
102.217
55.437
39.520 DMSO-d6
chemical shift（ppm）

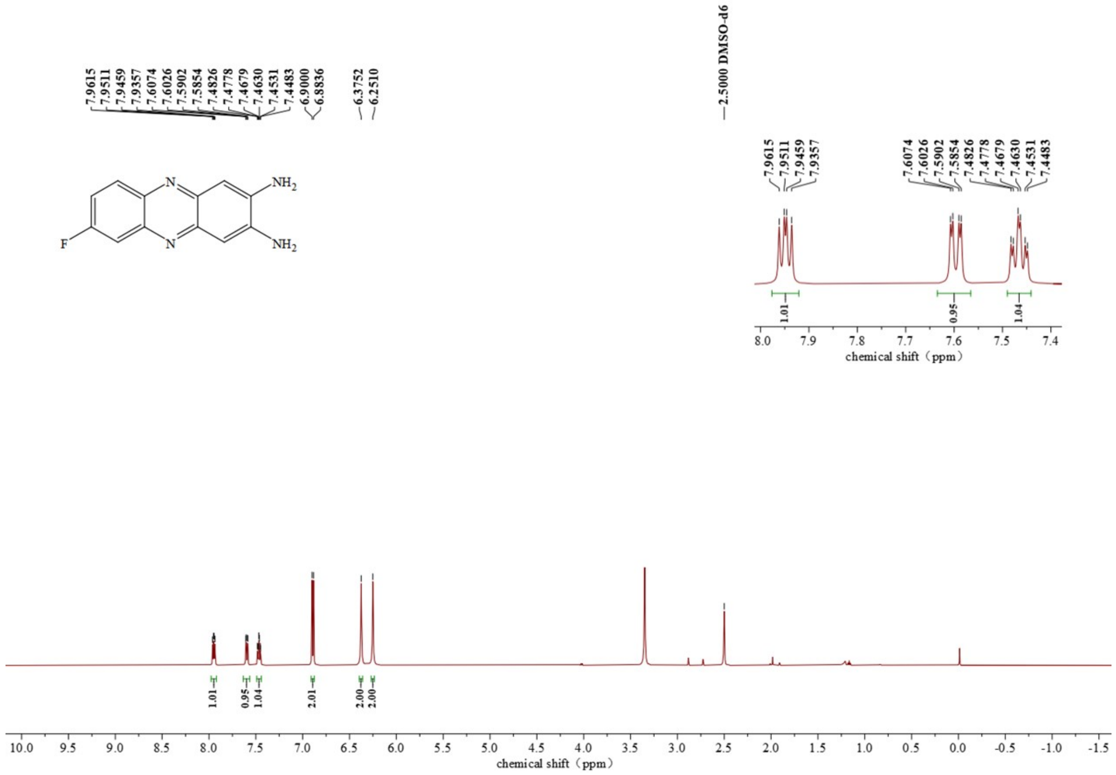
7.9615
7.9511
7.9459
7.9357
7.6074
7.6026
7.5902
7.5854
7.4826
7.4778
7.4679
7.4630
7.4531
7.4483
6.9000
6.8836
6.3752
6.2510
2.5000 DMSO-d6
NH_2
NH_2
N
N
F
1.01
0.95
1.04
2.01
2.00
2.00
chemical shift (ppm)

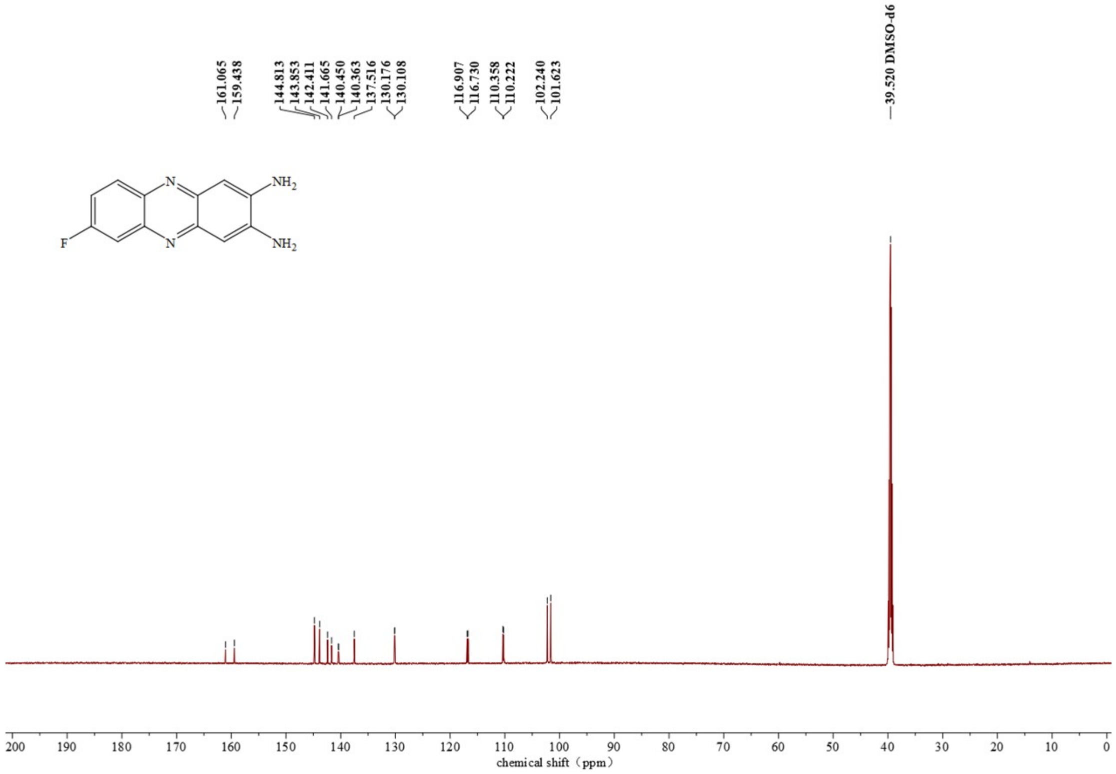
161.065
159.438
144.813
143.853
142.411
141.665
140.450
140.363
137.516
130.176
130.108
116.907
116.730
110.358
110.222
102.240
101.623
39.520 DMSO-d6
NH_2
NH_2
N
N
F
chemical shift (ppm)

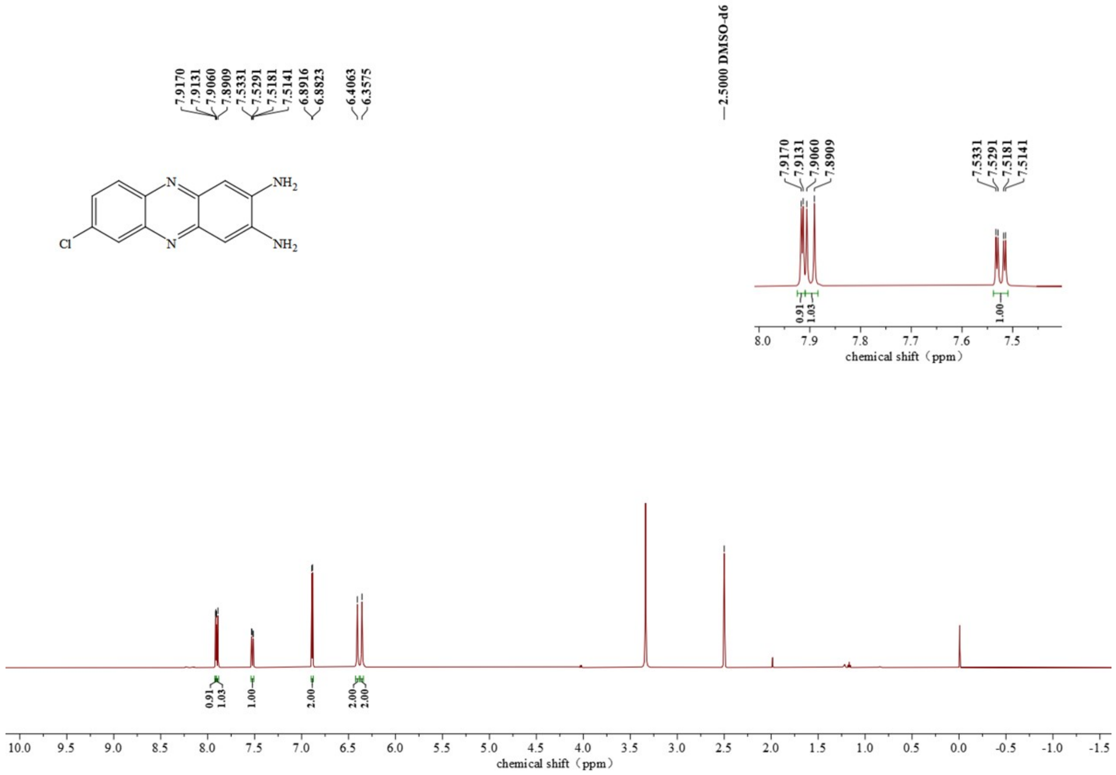
7.9170
7.9131
7.9060
7.8909
7.5331
7.5291
7.5181
7.5141
6.8916
6.8823
6.4063
6.3575
2.5000 DMSO-d6
Cl
N
NH2
0.91
1.03
1.00
2.00
chemical shift（ppm）

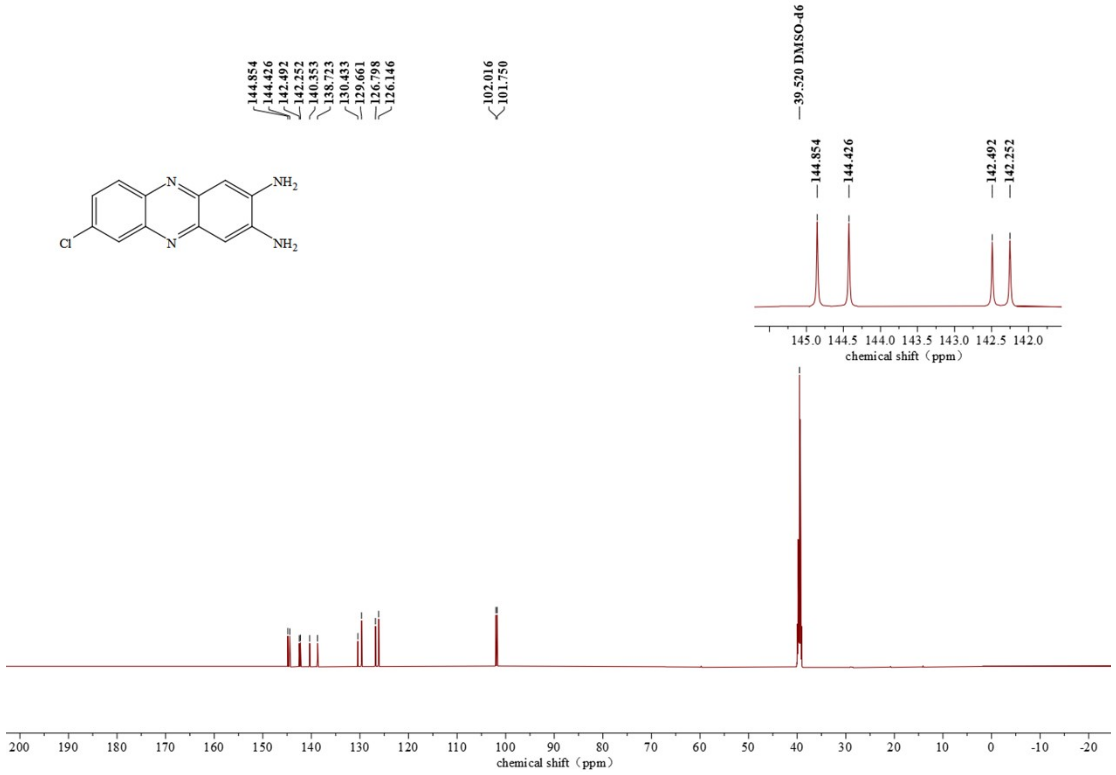
144.854
144.426
142.492
142.252
140.353
138.723
130.433
129.661
126.798
126.146
102.016
101.750
39.520 DMSO-d6
Cl
N
NH2
chemical shift（ppm）

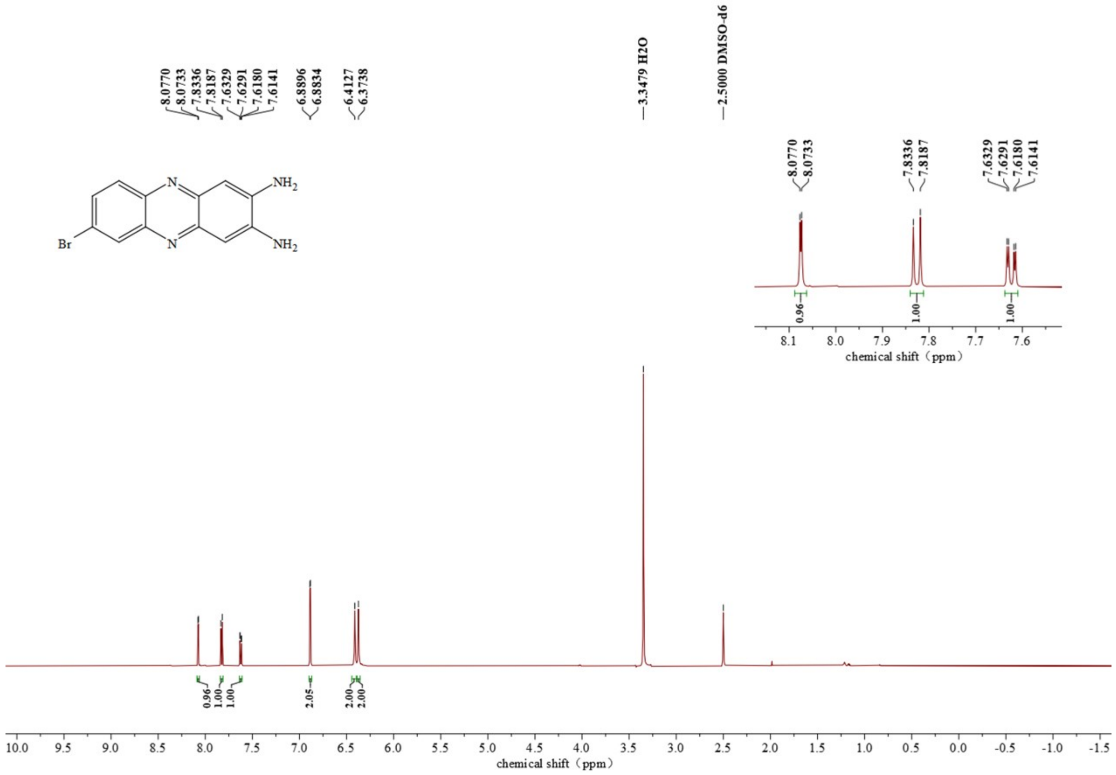
8.0770
8.0733
7.8336
7.8187
7.6329
7.6291
7.6180
7.6141
6.8896
6.8834
6.4127
6.3738
3.3479 H2O
2.5000 DMSO-d6
NH$_2$
NH$_2$
N
N
Br
0.96
1.00
1.00
2.05
2.00
2.00
chemical shift (ppm)

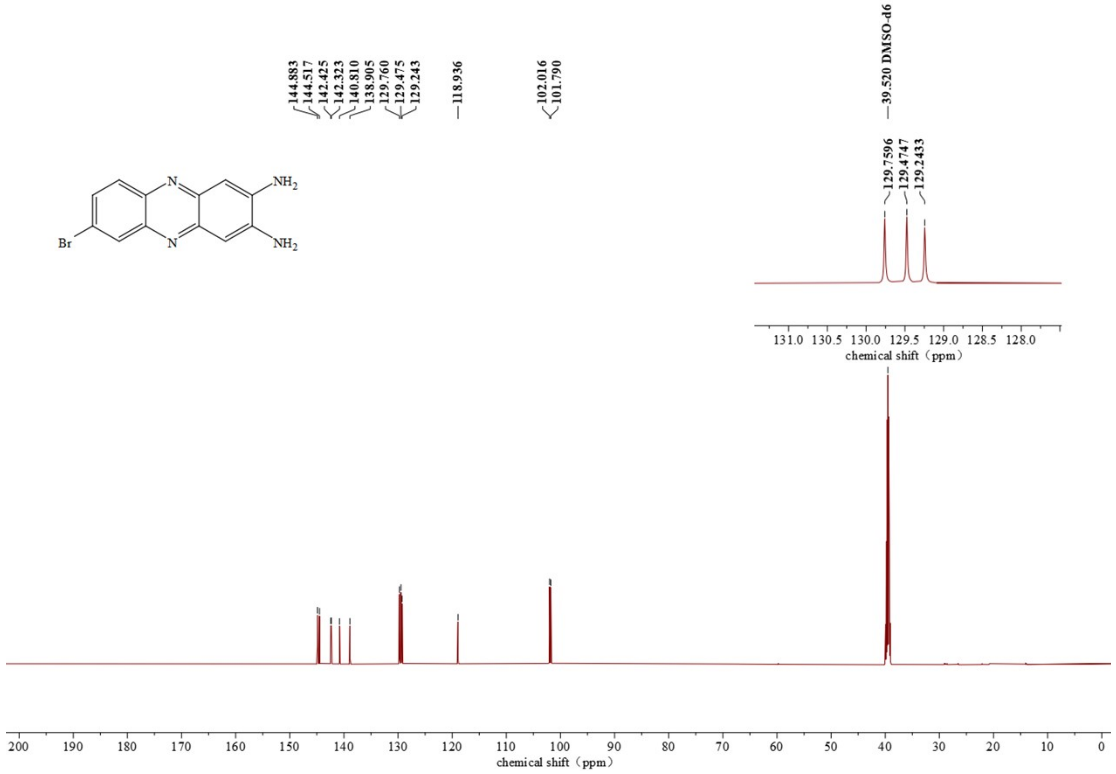
144.883
144.517
142.425
142.323
140.810
138.905
129.760
129.475
129.243
118.936
102.016
101.790
39.520 DMSO-d6
129.7596
129.4747
129.2433
NH$_2$
NH$_2$
N
N
Br
chemical shift (ppm)

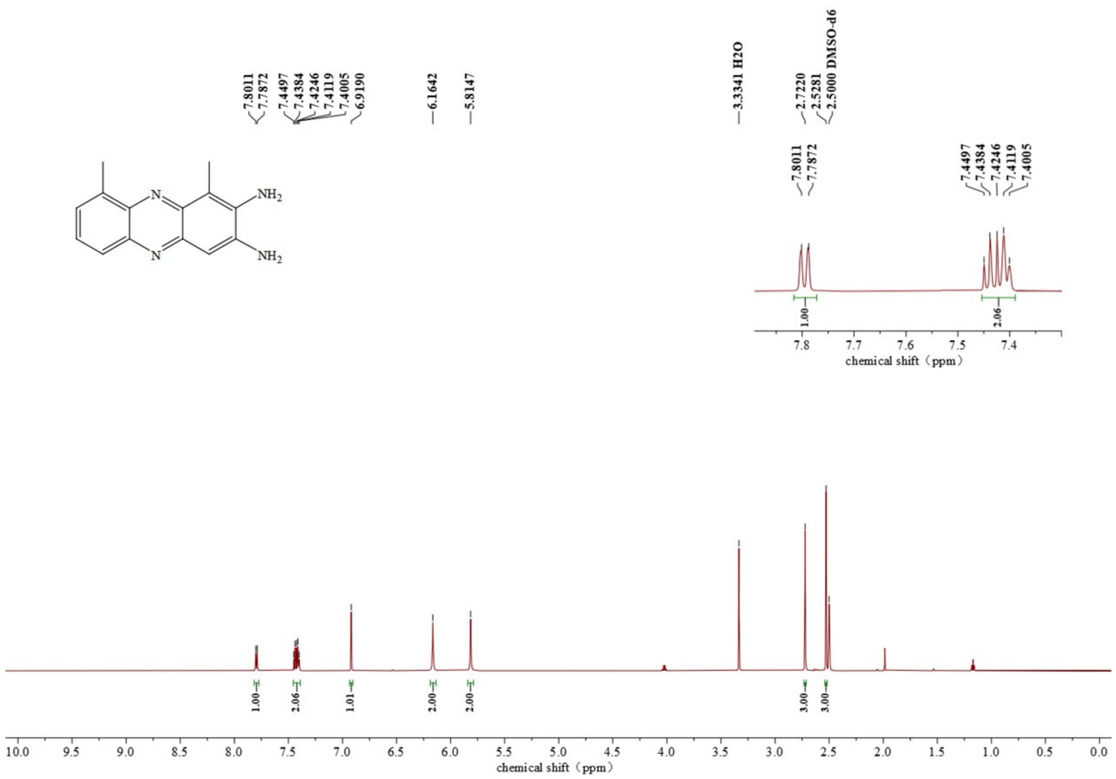
7.8011
7.7872
7.4497
7.4384
7.4246
7.4119
7.4005
6.9190
6.1642
5.8147
3.3341 H2O
2.7220
2.5281
2.5000 DMSO-d6
NH2
NH2
N
N
1.00
2.06
1.01
2.00
2.00
3.00
3.00
chemical shift（ppm）

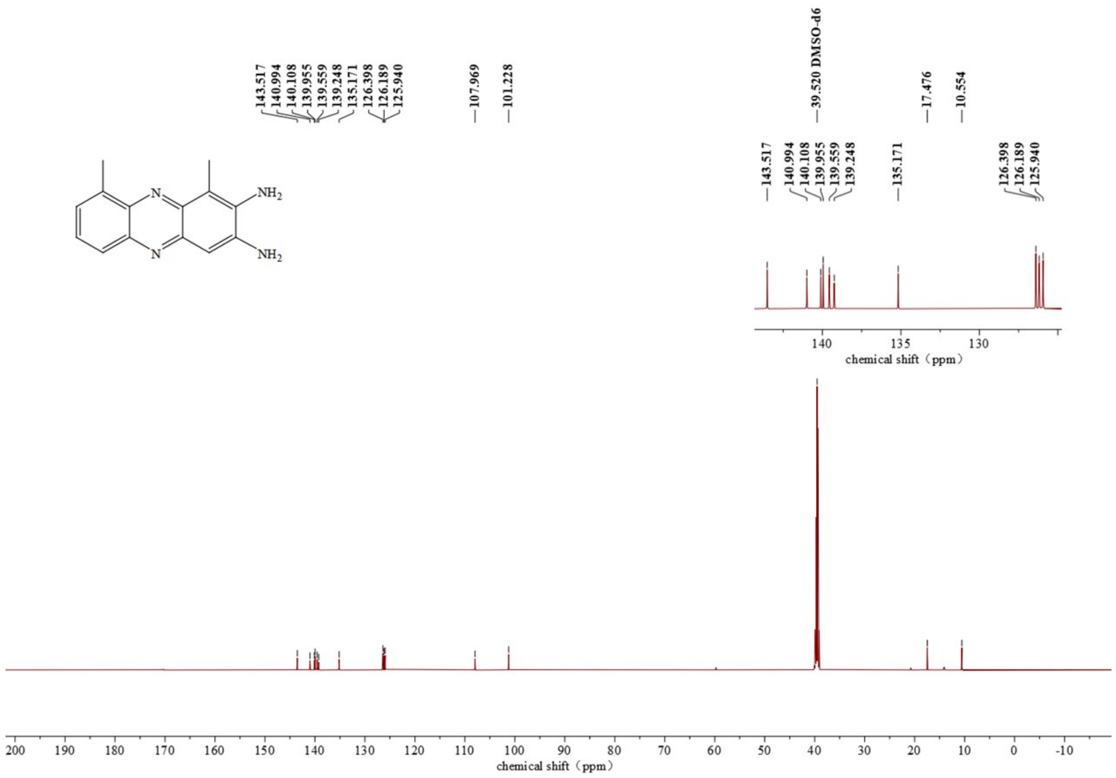
143.517
140.994
140.108
139.955
139.559
139.248
135.171
126.398
126.189
125.940
107.969
101.228
39.520 DMSO-d6
17.476
10.554
NH2
NH2
N
N
chemical shift（ppm）

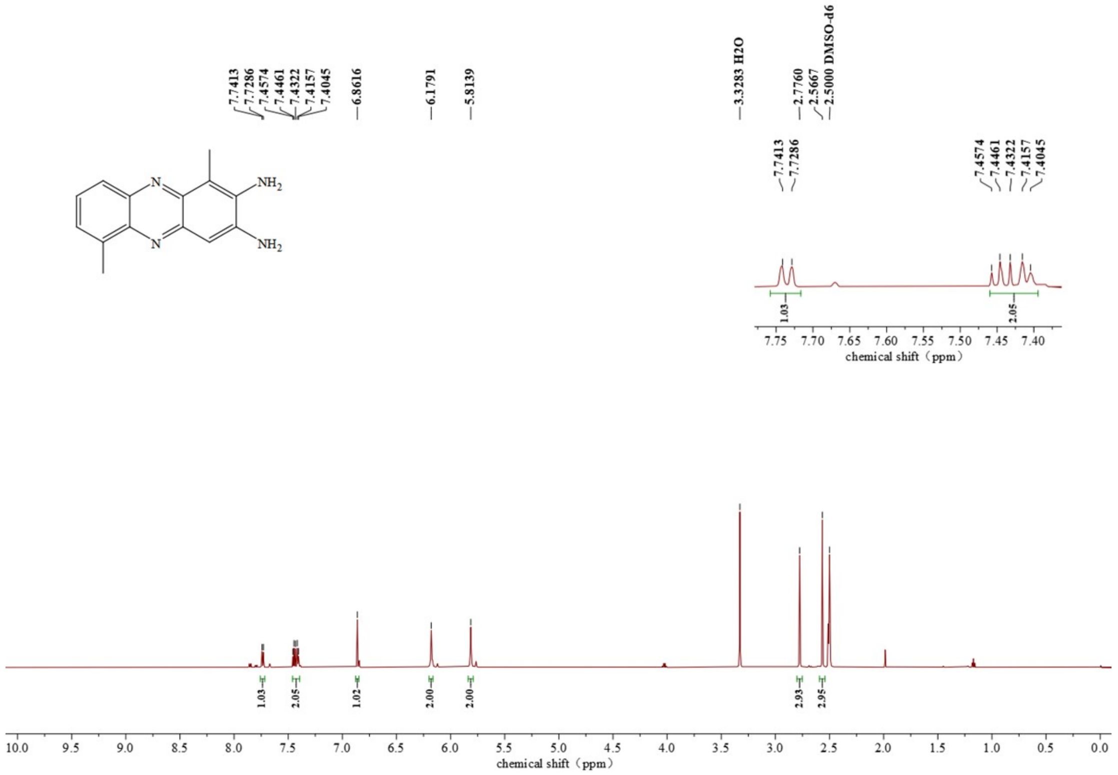
7.7413
7.7286
7.4574
7.4461
7.4322
7.4157
7.4045
6.8616
6.1791
5.8139
3.3283 H2O
2.7760
2.5667
2.5000 DMSO-d6
NH2
NH2
N
N
1.03
2.05
1.02
2.00
2.00
2.93
2.95
chemical shift (ppm)

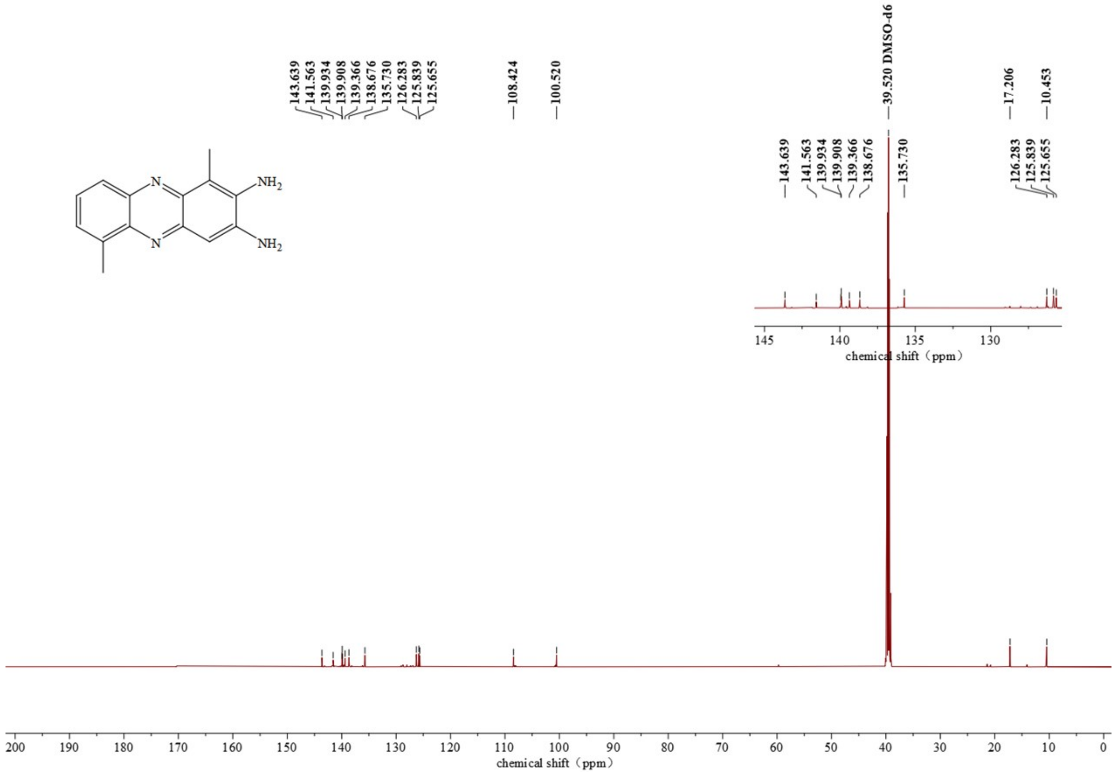
143.639
141.563
139.934
139.908
139.366
138.676
135.730
126.283
125.839
125.655
108.424
100.520
39.520 DMSO-d6
17.206
10.453
NH2
NH2
N
N
chemical shift (ppm)

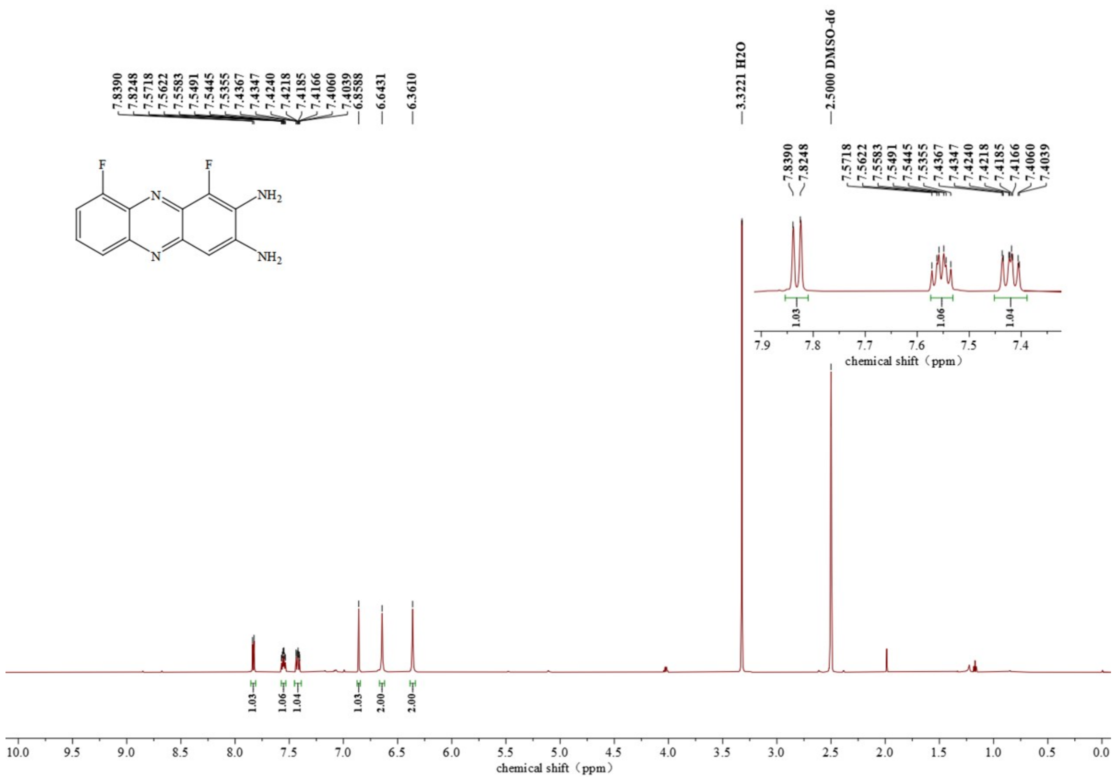

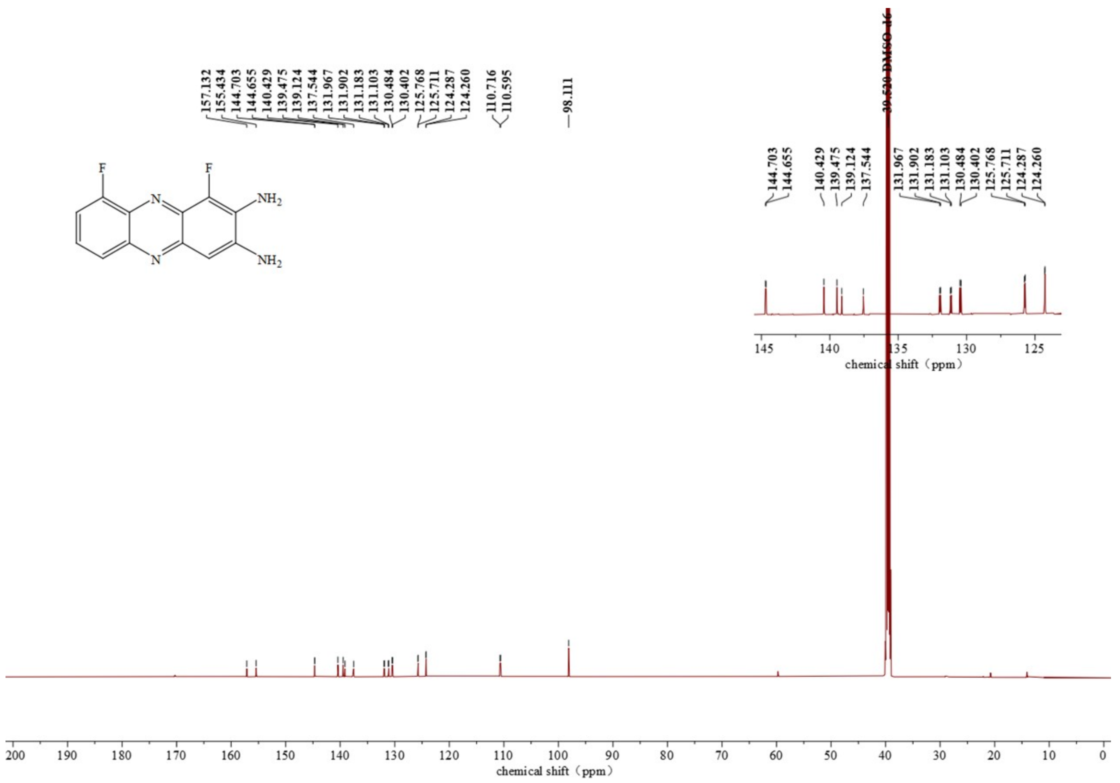

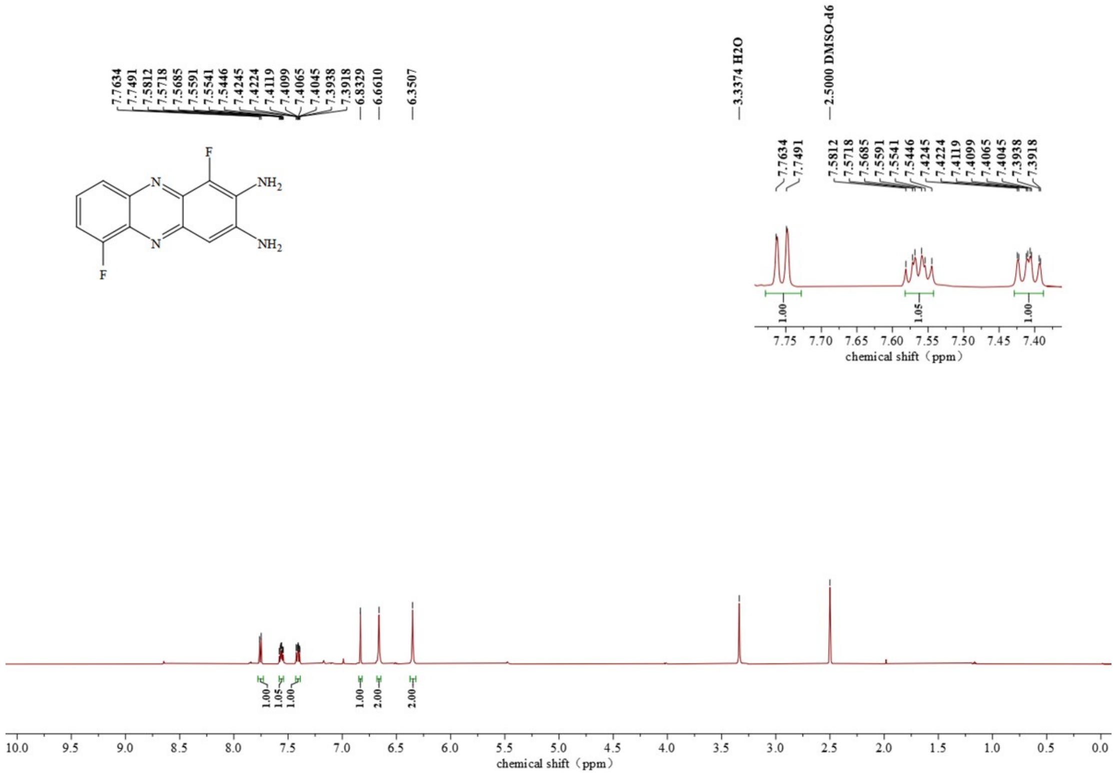
7.7634
7.7491
7.5812
7.5718
7.5685
7.5591
7.5541
7.5446
7.4245
7.4224
7.4119
7.4099
7.4065
7.4045
7.3938
7.3918
6.8329
6.6610
6.3507
3.3374 H2O
2.5000 DMSO-d6
F
N
N
NH2
NH2
F
1.00
1.05
1.00
1.00
2.00
2.00
chemical shift（ppm）

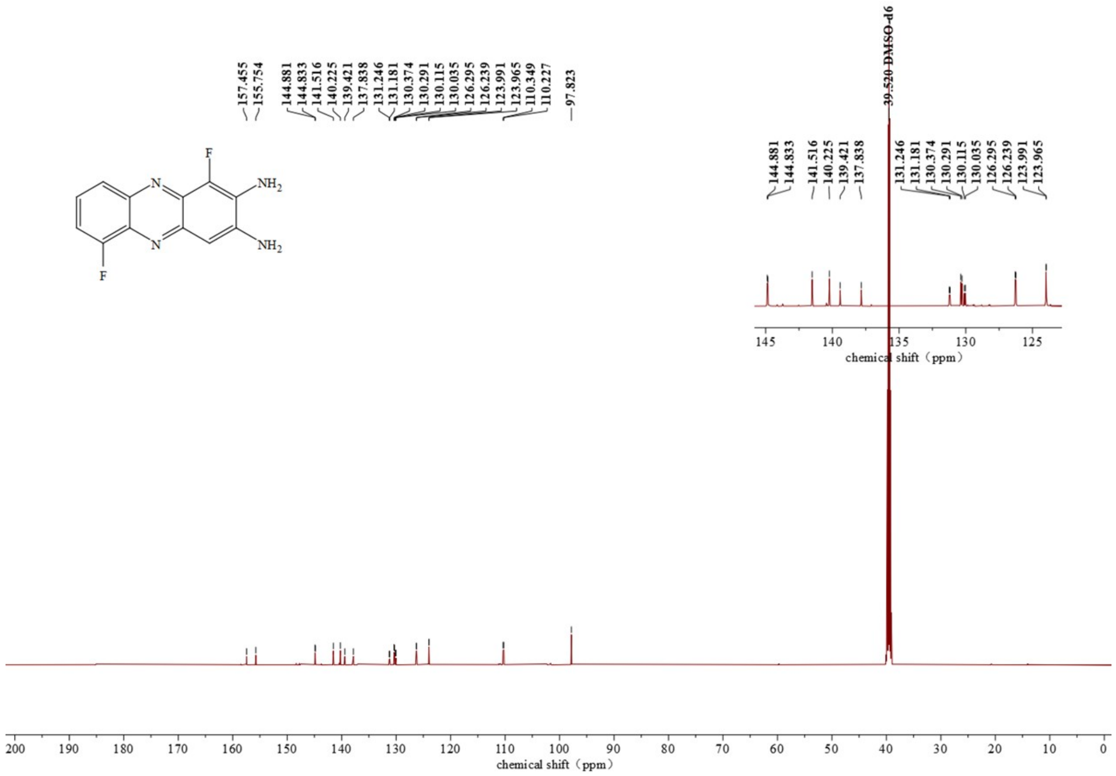
157.455
155.754
144.881
144.833
141.516
140.225
139.421
137.838
131.246
131.181
130.374
130.291
130.115
130.035
126.295
126.239
123.991
123.965
110.349
110.227
97.823
39.520 DMSO-d6
F
N
N
NH2
NH2
F
chemical shift（ppm）

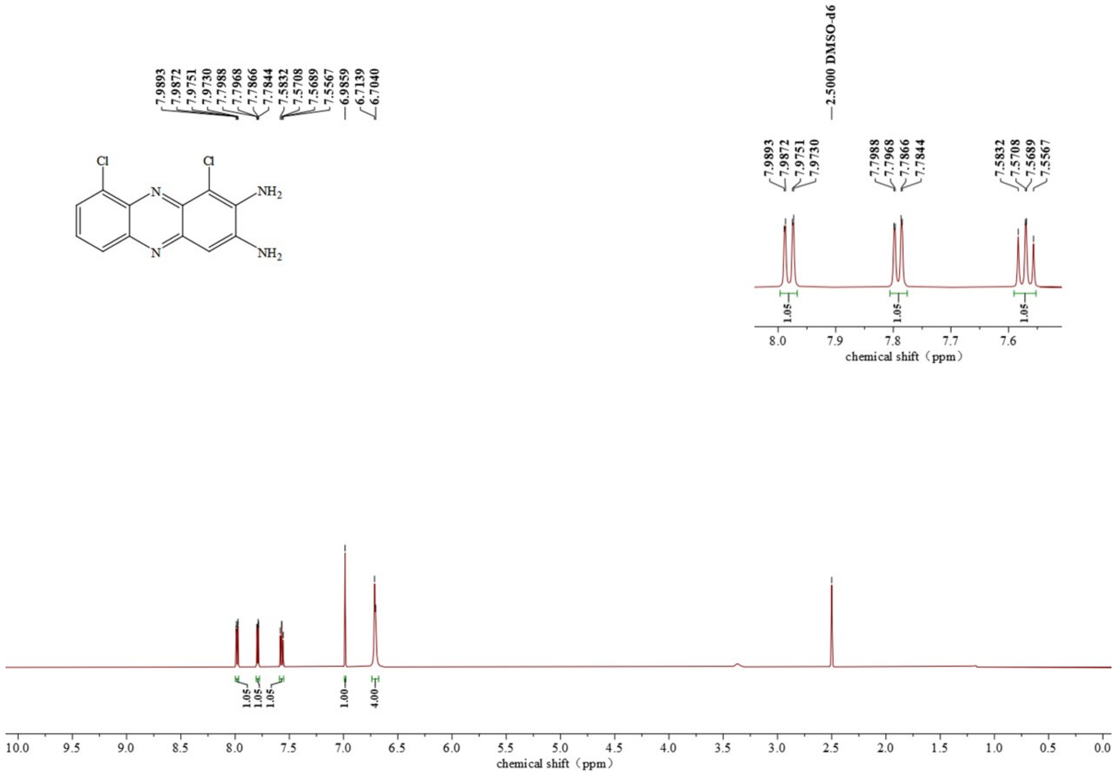
7.9893
7.9872
7.9751
7.9730
7.7988
7.7968
7.7866
7.7844
7.5832
7.5708
7.5689
7.5567
6.9859
6.7139
6.7040
2.5000 DMSO-d6
Cl
Cl
N
N
NH2
NH2
1.05
1.05
1.05
1.00
4.00
chemical shift（ppm）

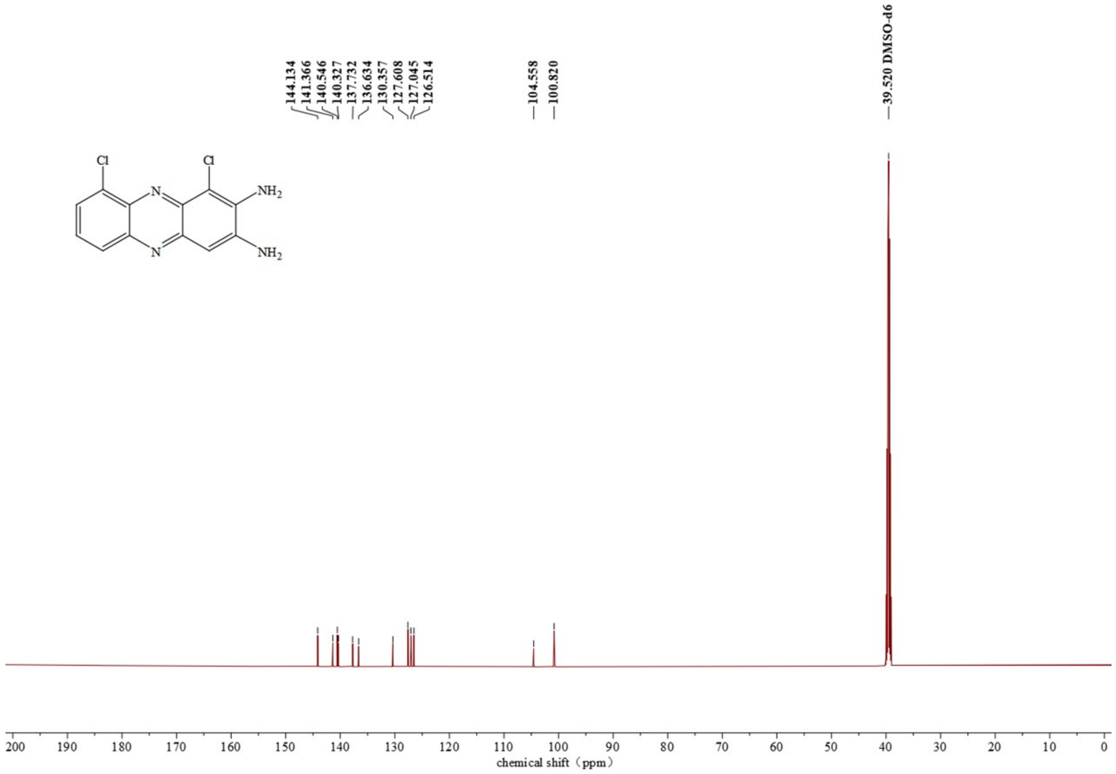
144.134
141.366
140.546
140.327
137.732
136.634
130.357
127.608
127.045
126.514
104.558
100.820
39.520 DMSO-d6
Cl
Cl
N
N
NH2
NH2
chemical shift（ppm）

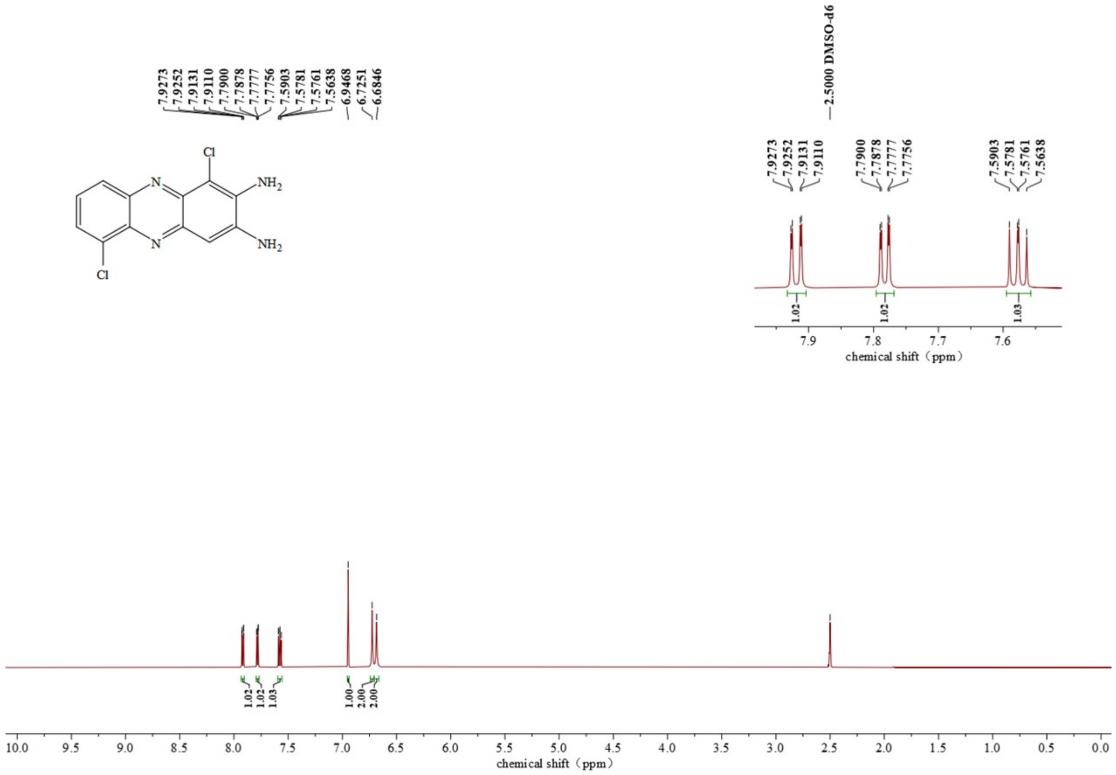

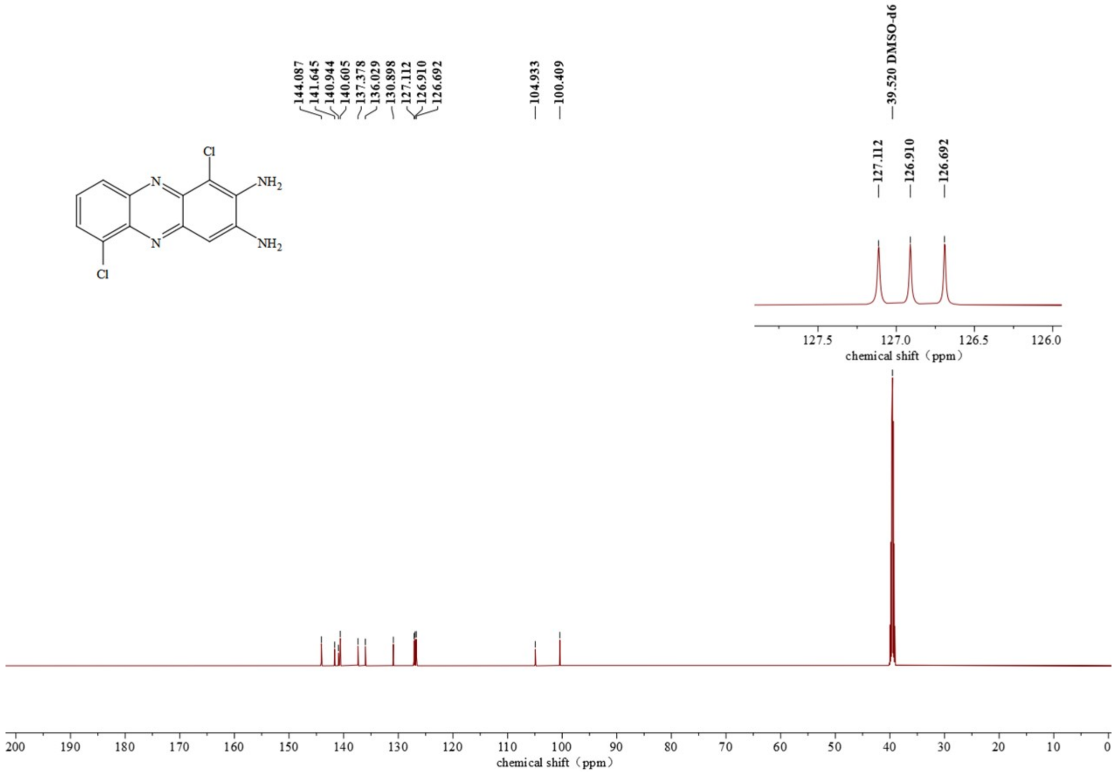

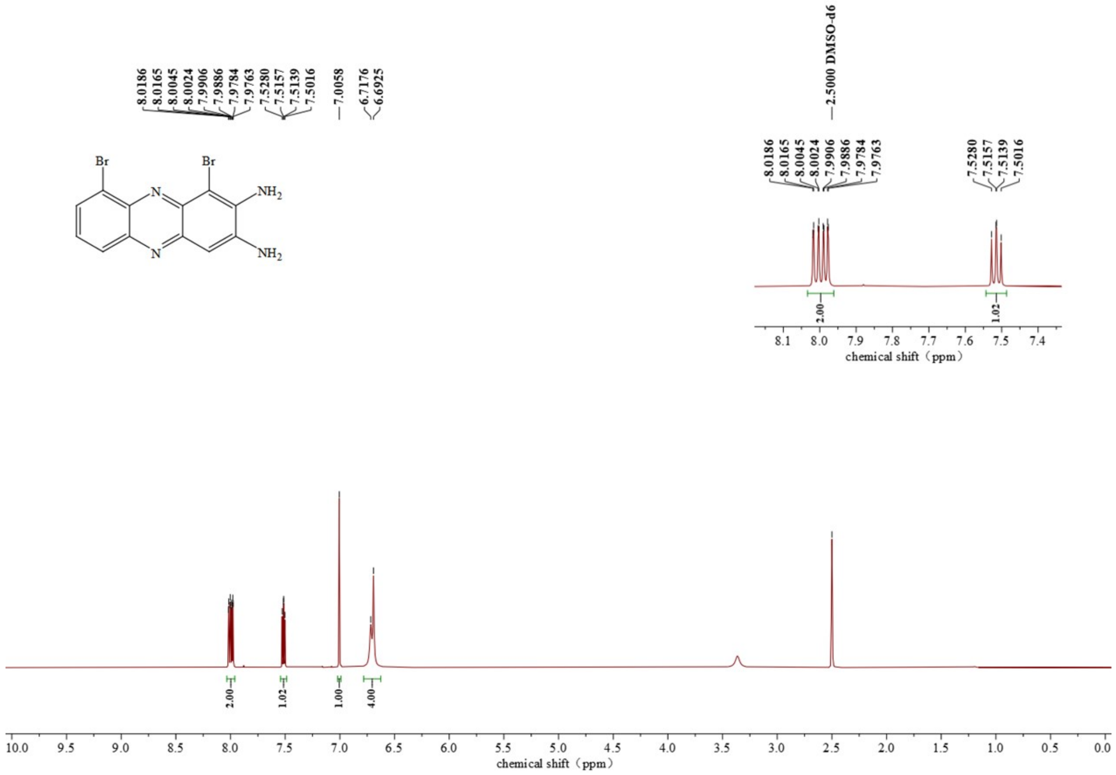
8.0186
8.0165
8.0045
8.0024
7.9906
7.9886
7.9784
7.9763
7.5280
7.5157
7.5139
7.5016
7.0058
6.7176
6.6925
2.5000 DMSO-d6
Br
Br
N
N
NH2
NH2
2.00
1.02
1.00
4.00
chemical shift（ppm）

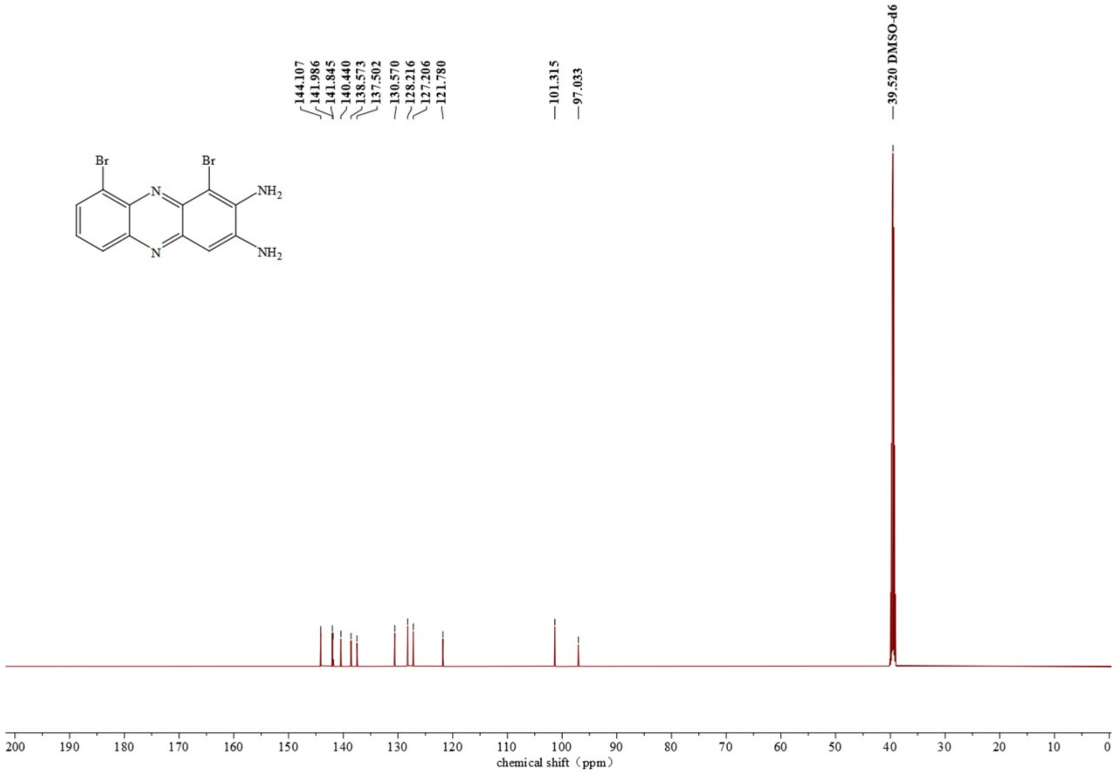
144.107
141.986
141.845
140.440
138.573
137.502
130.570
128.216
127.206
121.780
101.315
97.033
39.520 DMSO-d6
Br
Br
N
N
NH2
NH2
chemical shift（ppm）

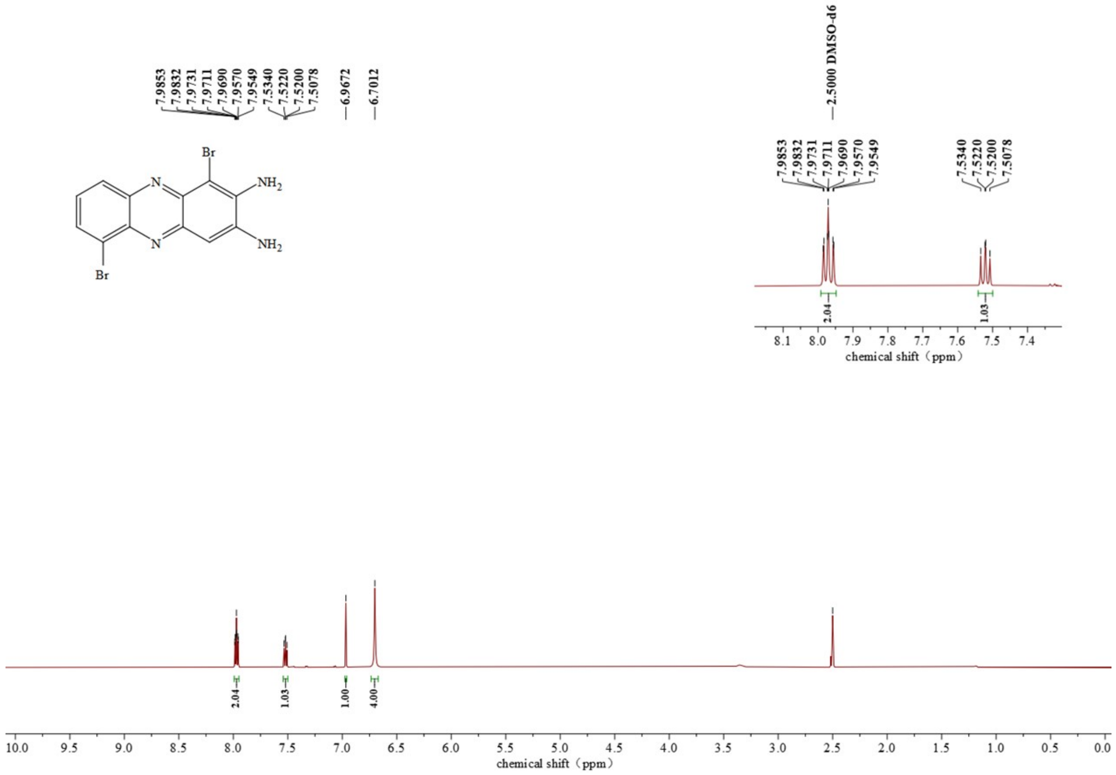
7.9853
7.9832
7.9731
7.9711
7.9690
7.9570
7.9549
7.5340
7.5220
7.5200
7.5078
6.9672
6.7012
2.5000 DMSO-d6
Br
N
N
NH2
NH2
Br
2.04
1.03
1.00
4.00
chemical shift（ppm）

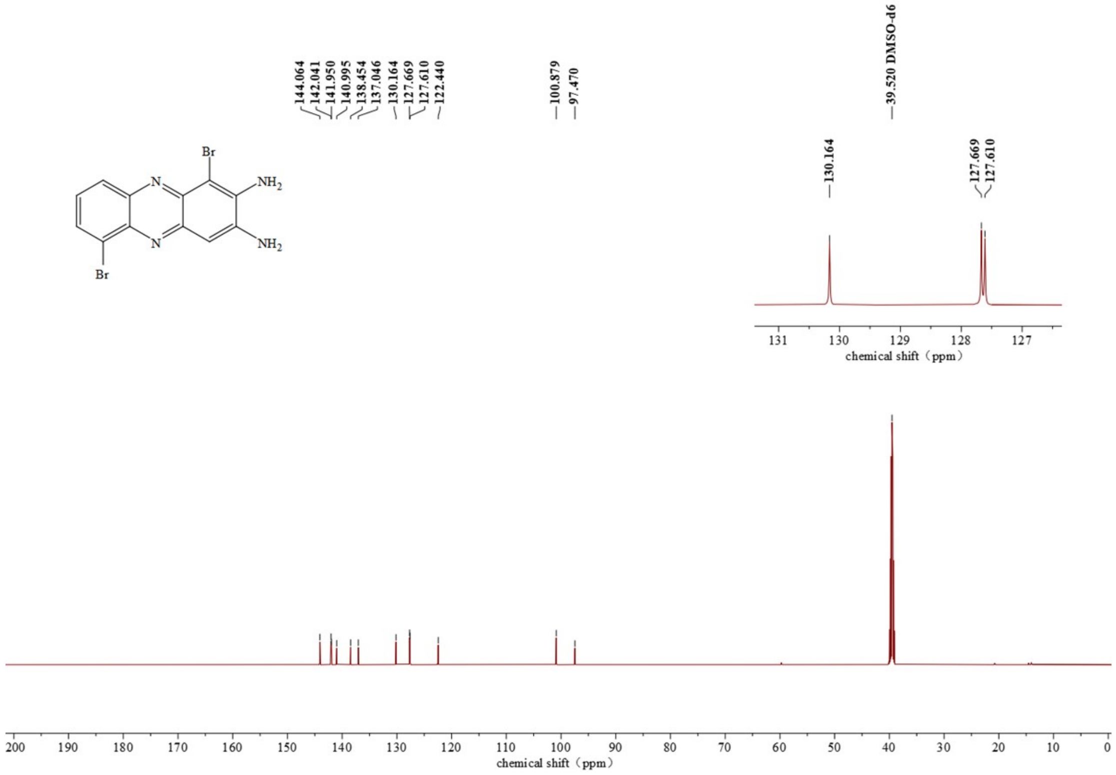
144.064
142.041
141.950
140.995
138.454
137.046
130.164
127.669
127.610
122.440
100.879
97.470
39.520 DMSO-d6
Br
N
N
NH2
NH2
Br
130.164
127.669
127.610
chemical shift（ppm）
